# Solvent-programmable organocatalyzed group transfer copolymerization (O-GTcP) of muconate esters: from statistical control to copolymerization-induced self-assembly

**DOI:** 10.1039/d6sc05973b

**Published:** 2026-10-01

**Authors:** José David Estrada Sotomayor, Thomas Dardé, Émilie Diomar, Xavier Schultze, Guillaume Fleury, Daniel Taton

**Affiliations:** a Univ. Bordeaux, CNRS, Bordeaux INP, LCPO, UMR 5629 Pessac F-33600 France taton@enscbp.fr; b L’Oréal Research & Innovation 1 Avenue Eugène Schueller Aulnay Sous-Bois 93600 France

## Abstract

Developing sustainable polymer platforms requires simultaneous control over micro- and macromolecular structures, material properties, and end-of-life fate. Herein, we report the one-pot organocatalyzed group transfer copolymerization (O-GTcP) of dioctyl and diethyl muconates (DOM and DEM), achieving statistical copolymuconates with tunable properties and composition-dependent self-assembly. In homogeneous media (THF and toluene), phosphazene-mediated O-GTcP proceeds very rapidly at room temperature (<15 min), yielding statistical copolymers with controlled composition and tunable glass-transition temperatures. These materials undergo efficient thermal depolymerization, regenerating the parent muconate monomers, which can be directly repolymerized without monomer purification to close the polymer–monomer–polymer cycle. Remarkably, conducting the same copolymerization in isododecane leads to a distinct assembly regime. While poly(DOM) remains soluble in this nonpolar medium, increasing DEM incorporation progressively reduces copolymer solubility and ultimately triggers *in situ* nanoparticle formation during copolymer chain growth. Stable dispersions are thus generated directly through one-pot copolymerization-induced self-assembly under organocatalytic conditions (O-GTcPISA), without block copolymer design or sequential polymerization steps. Together, these results establish O-GTcP as a solvent-programmable platform integrating rapid metal-free copolymerization, statistical sequence control, recyclable backbone chemistry, and nanoscale organization within a single synthetic framework.

## Abstract 2

Organocatalyzed group transfer copolymerization of muconate esters enables a solvent-programmed transition, from molecularly dissolved statistical copolymers in homogeneous solution, to nanoparticles formed during copolymer growth in nonpolar media.

## Graphical abstract

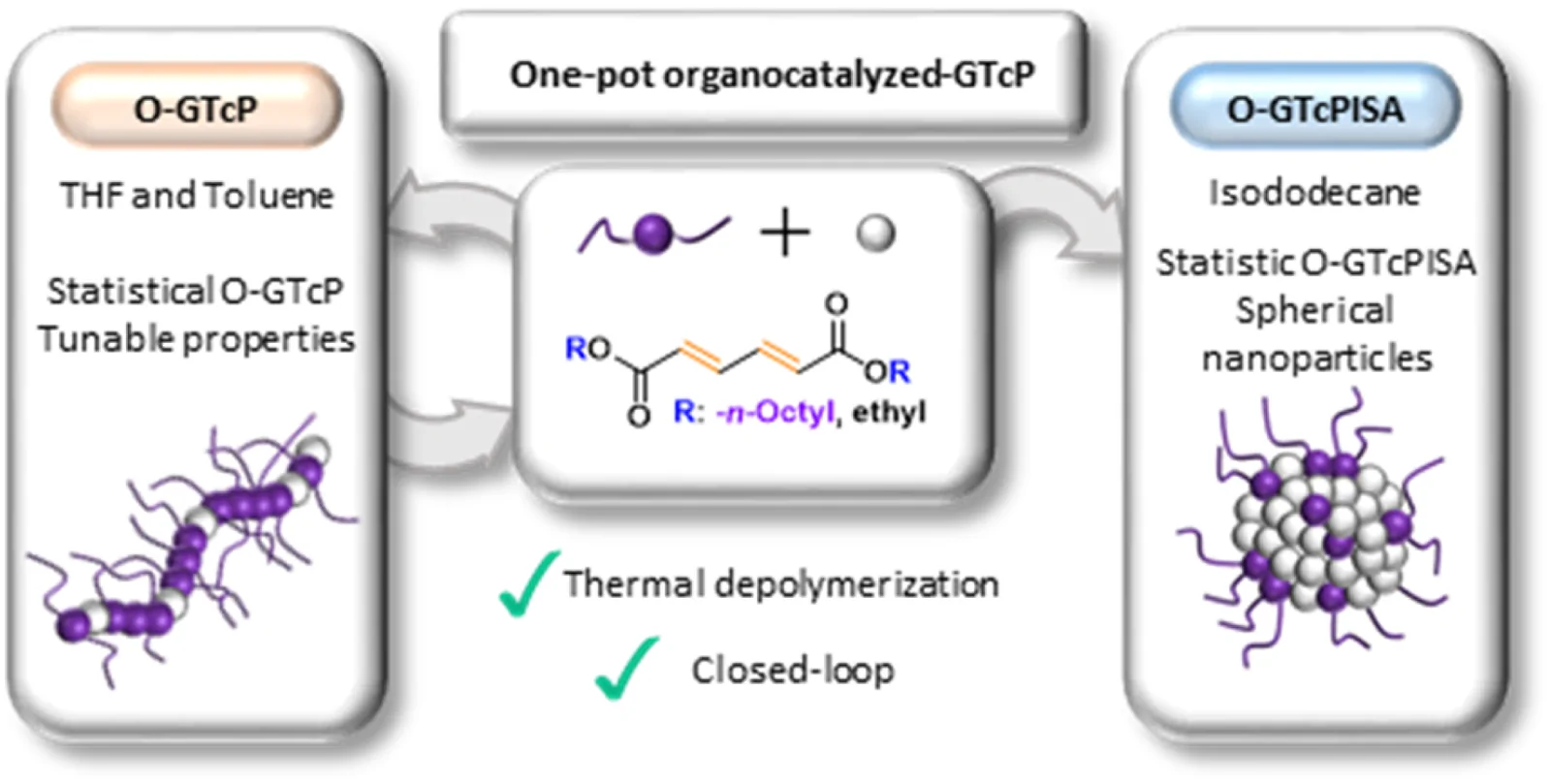

## 1 Introduction

The transition toward sustainable polymeric materials requires strategies that integrate renewable feedstocks, precise macromolecular engineering, and end-of-life design within unified synthetic platforms.^1,2^ While monomer selection is a primary determinant of performance, the sequence distribution of comonomer units along copolymeric chains, *i.e.*, copolymer microstructure, often exerts a profound influence on thermomechanical behaviour, solubility, and self-assembly. In this regard, statistical copolymerization represents a straightforward and powerful strategy enabling the continuous tuning of material properties through composition.^3,4^ Bio-based monomers provide an important opportunity to align such precision synthesis with sustainability goals.^5–9^

Among emerging bio-based building blocks, muconic acid and its dialkyl muconate derivatives, which are accessible from lignocellulosic feedstocks *via* fermentation routes, have emerged as promising renewable alternatives to petroleum-derived acrylic monomers.^6,10^ More generally, muconic acid has attracted increased attention as a platform molecule capable of bridging bio-based carbon sources with performance polymers.^10–16^ Its conjugated diene structure grants access to polymuconates, while introducing inherent chemical handles that can be leveraged for post-polymerization modification and programmed depolymerization.^13–15^ Muconate derivatives thus offer a unique opportunity to combine renewable origin with intrinsic chemical degradability in a single macromolecular framework.^11–13,15^ Precision control over such unsaturated monomers, however, requires a polymerization method capable of delivering well-defined architectures under scalable conditions. In this regard, dialkyl muconates are particularly challenging substrates, as their radical polymerization whether controlled or conventional, in bulk or in solution-typically proceeds sluggishly, often requiring several hours to reach incomplete conversion.^11,17^ Group transfer polymerization (GTP) effectively overcomes this kinetic limitation, enabling quantitative muconate conversion within minutes at room temperature under metal-free conditions, while affording polymuconates with well-defined structures.^14,15^ Historically, GTP was established as an important platform for precise methacrylic polymer synthesis.^18,19^ Over the past two decades, renewed interest in GTP has emerged through the development of organocatalytic variants, broadening the monomer scope beyond conventional methacrylates to include more challenging and structurally diverse renewable monomers, such as itaconates,^20,21^ sorbates,^22,23^ crotonates,^24,25^ and muconates.^14,15^

In parallel, polymerization-induced self-assembly (PISA) has transformed the preparation of polymer nanoparticles and latexes by coupling polymer synthesis with *in situ* self-organization.^26–30^ In conventional PISA formulations, self-assembly is typically achieved through sequential living polymerization, whereby a solvophilic precursor block is first synthesized and subsequently chain-extended with a progressively solvophobic segment. The resulting amphiphilic block copolymers undergo *in situ* organization into concentrated, surfactant-free nanostructures with tunable particle sizes and morphologies. Owing to its robustness, PISA has become predominantly associated with controlled radical polymerization techniques. Beyond these canonical block-copolymer strategies, much fewer studies have explored one-pot copolymerization-induced self-assembly processes. In such cases, one-step formulations rely on strongly differentiated monomer reactivities that generate gradient or pseudo-block microstructures during copolymerization, thereby preserving an amphiphilic segmented structure responsible for self-assembly.^31–33^ By contrast, examples in which self-assembly arises directly from the growth of genuinely statistical copolymers remain exceedingly rare.^34–37^ To the best of our knowledge, only two related studies have demonstrated such behaviour, without the need of solvent-switch, both based on reversible addition–fragmentation chain-transfer (RAFT) mediated statistical copolymerization in aqueous media.^34,35^ In these systems, the progressive incorporation of solvophobic repeating units during random copolymer growth induced nanoparticle formation without requiring sequential synthesis. These precedents highlight the possibility that statistical copolymer growth can gradually alter polymer solubility and trigger self-assembly once a critical composition and chain length are reached.

Herein, we combine bio-based muconate chemistry, organocatalyzed GTP, and PISA within a single synthetic platform. We first report the organocatalyzed group transfer copolymerization (O-GTcP) of dioctyl and diethyl muconates (DOM and DEM), yielding predominantly statistical copolymers with tunable composition and thermomechanical properties in homogeneous media (THF and toluene). The resulting copolymers further undergo efficient thermal depolymerization to regenerate the parent muconate monomers, which can be directly repolymerized without monomer purification, thereby demonstrating a closed-loop polymer–monomer–polymer cycle. Remarkably, translating the same copolymerization to isododecane, a selective solvent for poly(DOM), induces a transition from homogeneous polymerization to *in situ* nanoparticle formation. Increasing DEM incorporation progressively reduces copolymer solubility, ultimately triggering copolymerization-induced self-assembly directly during statistical copolymer growth, *i.e.* without block copolymer design, surfactants, or sequential polymerization steps. To the best of our knowledge, this constitutes the first example of organocatalyzed group transfer copolymerization-induced self-assembly (O-GTcPISA) and one of the very few examples in which self-assembly emerges from statistical copolymerization rather than from deliberately segmented microstructures. By thus coupling renewable monomers, statistical copolymerization, closed-loop recyclability, and nanoscale organization within a rapid metal-free polymerization process, this work establishes O-GTcP as a versatile platform for sustainable precision polymer synthesis.

## 2 Results and discussion

### 2.1 One-pot O-GTcP of DEM and DOM in homogeneous THF or toluene media

GTP has previously been demonstrated as a viable route for copolymerizing monomers of distinct structures.^38–40^ For instance, Chen *et al.* reported the GTcP of α-methylene-γ-butyrolactone and γ-methyl-α-methylene-γ-butyrolactone using trityl tetrakis(pentafluorophenyl)borate as catalyst, affording copolymers with intermediate glass transition temperatures.^41^ In the present work, we sought to determine whether O-GTcP could achieve true statistical copolymers from two distinct dialkyl muconates, namely, diethyl muconate (DEM) and dioctyl muconate (DOM). Simultaneous one-pot O-GTcP experiments were thus conducted by introducing mixtures of DEM and DOM into solutions containing the silyl ketene acetal initiator (1-ethoxy-1-(trimethylsiloxy)-1,3-butadiene, ETSB) and the phosphazene base P_4_-*t-*Bu in toluene at room temperature. The initial feed composition was varied, targeting a total degree of polymerization of 100. Scheme 1 shows the general synthesis method, while Table 1 summarizes the main results obtained. A first series of copolymerization experiments employed the following conditions: [DEM + DOM]_0_/[ETSB]_0_/[P_4_-*t-*Bu]_0_ of 100/1/0.01 (Table 1, runs 1–4), leading to near-complete conversion of both monomers in just a few minutes. Size exclusion chromatography (SEC) revealed unimodal molar mass distributions for all copolymers, with no additional major populations attributable to competing polymerization pathways (Fig. S1). However, dispersity values (*Đ*) were around 1.5, indicating only moderate control over the molar mass distribution (Fig. 1A and S1). This behaviour mirrors our previous observations for poly(dialkyl muconate) synthesis by O-GTP using P_4_-*t*-Bu in toluene, where ultrafast polymerization kinetics (complete conversion within minutes; Fig. S2) were accompanied by broader molar-mass distributions.^14^ The very high propagation rates relative to the exchange processes responsible for activation and deactivation can limit equilibration during chain growth (Scheme S1),^15^ an effect that may be further influenced by the resonance stabilization of the conjugated dienolate propagating species. Importantly, the number-average molar mass (*M*_n_) increased linearly with monomer conversion (Fig. S1), consistent with chain-growth under these kinetically accelerated conditions.

**Scheme 1:**
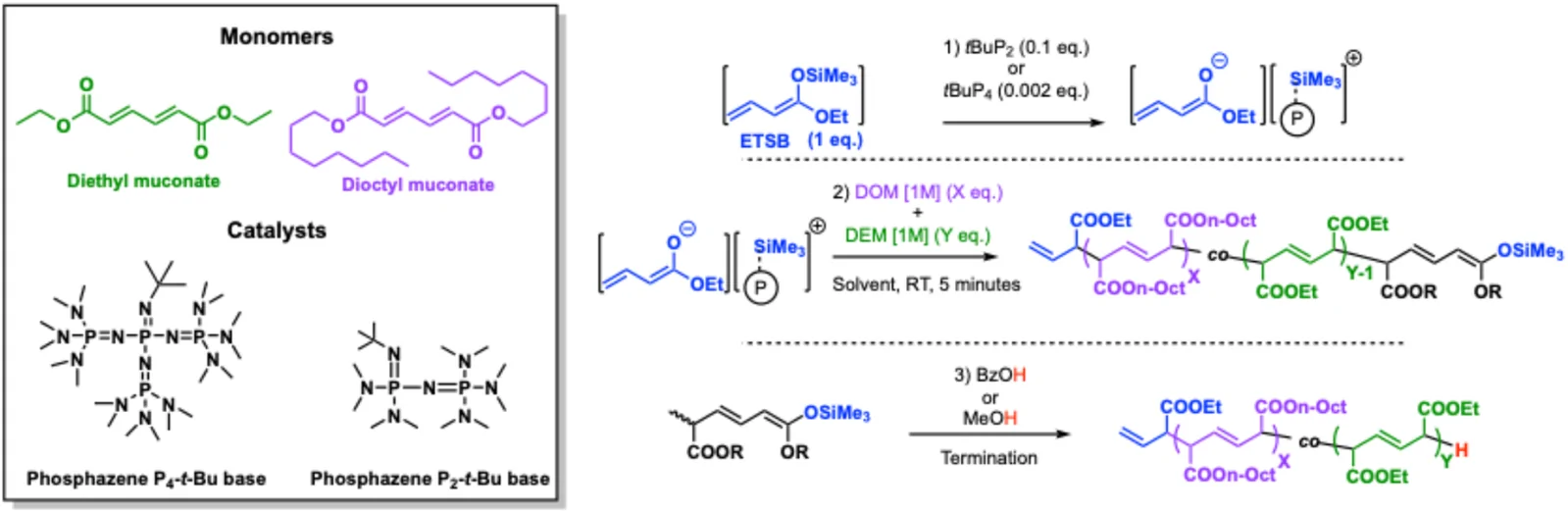
General protocol for the one-pot organocatalyzed group transfer copolymerization (O-GTcP) of diethyl muconate (DEM) and dioctyl muconate (DOM) using phospazene bases, P_4_-*t*-Bu and P_2_-*t*-Bu, as catalysts.

**Table 1:** Phosphazene-mediated O-GTcP of DEM and DOM at 25 °C

Entry	Catalyst	Solvent	Time (min)	[DEM + DOM]_0_/[I]_0_/[C]_0_^a^	Conv. (%) [DEM + DOM]	*M*_n,theo._ b (g mol^−1^)	*M*_n,SEC_ c (g mol^−1^)	*M*_n,MALS_ d (g mol^−1^)	*Đ* e
1	P_4_-*t*-Bu	Toluene	1	20 + 80/1/0.01	>99	33 200	54 000	61 100	1.54
2	P_4_-*t*-Bu	Toluene	1	40 + 60/1/0.01	>99	29 900	52 800	59 300	1.62
3	P_4_-*t*-Bu	Toluene	1	60 + 40/1/0.01	>99	26 500	50 100	52 200	1.48
4	P_4_-*t*-Bu	Toluene	1	80 + 20/1/0.01	>99	23 200	49 900	51 200	1.56
5	P_4_-*t*-Bu	THF	1	20 + 80/1/0.01	>99	33 200	54 900	62 100	1.16
6	P_4_-*t*-Bu	THF	1	40 + 60/1/0.01	>99	29 900	46 600	46 700	1.23
7	P_4_-*t*-Bu	THF	1	60 + 40/1/0.01	>99	26 500	58 600	61 200	1.17
8	P_4_-*t*-Bu	THF	1	80 + 20/1/0.01	>99	23 200	52 300	53 700	1.16
9	P_2_-*t*-Bu	THF	5	20 + 80/1/0.1	>99	33 200	24 000	25 500	1.15
10	P_2_-*t*-Bu	THF	5	40 + 60/1/0.1	>99	29 900	20 300	22 800	1.17
11	P_2_-*t*-Bu	THF	5	60 + 40/1/0.1	>99	26 500	20 700	21 300	1.15
12	P_2_-*t*-Bu	THF	5	80 + 20/1/0.1	>99	23 200	17 500	19 400	1.15
13	P_2_-*t*-Bu	THF	5	50 + 50/1/0.1	>99	28 200	22 200	—	1.16
14^f^	P_2_-*t*-Bu	Toluene	60	50 + 50/1/0.1	0	28 200	—	—	—

a [DEM + DOM]_0_/[I]_0_/[C]_0_ represents the ratio [DEM + DOM]_0_/[ETSB]_0_/[Catalyst]_0_.

b Theoretical number molar mass calculated with ([DEM + DOM]_0_/[ETSB]_0_) × (*M*_W_ of DEM, 198.22 g mol^−1^ and *M*_W_ of DOM, 366.54 g mol^−1^) + chain ends (–C_6_H_9_O_2_ and –H; 114.1 g mol^−1^).

c Average molar mass measured by SEC with PMMA calibration.

d Average molar mass measured by SEC with MALS, using d*n*/d*c* values experimentally determined and presented in Table S2.

e Dispersity value as determined by SEC with PMMA calibration with dRI detector.

f No polymerization was observed.

**Table 2:** O-GTcP of diethyl and dioctyl muconates at 25 °C in isododecane

Entry	Catalyst	Time (min)	[DEM + DOM]_0_/[I]_0_/[C]_0_^a^	*M*_n,theo._ b (g mol^−1^)	*M*_n,SEC_ c (g mol^−1^)	*Đ* d
1	P_4_-*t*-Bu	10	10 + 90/1/0.01	34 900	35 600	1.57
2	P_4_-*t*-Bu	10	20 + 80/1/0.01	33 200	34 800	1.54
3	P_4_-*t*-Bu	10	30 + 70/1/0.01	31 600	33 000	1.52
4	P_4_-*t*-Bu	10	50 + 50/1/0.01	28 200	27 500	1.57
5	P_4_-*t*-Bu	10	60 + 40/1/0.01	26 500	29 300	1.96
6	P_4_-*t*-Bu	10	70 + 30/1/0.01	24 800	23 500	1.93
7	P_4_-*t*-Bu	10	80 + 20/1/0.01	23 200	25 300	2.73
8	P_4_-*t*-Bu	10	90 + 10/1/0.01	21 500	38 900	2.03
9	P_4_-*t*-Bu	10	40 + 160/1/0.1	66 500	76 100	1.51
10	P_4_-*t*-Bu	10	100 + 100/1/0.1	56 400	63 000	1.50
11	P_2_-*t*-Bu	60	50 + 50/1/0.1	28 200	0	0

a [DEM + DOM]_0_/[I]_0_/[C]_0_ represents the ratio [DEM + DOM]_0_/[ETSB]_0_/[Catalyst]_0_.

b Theoretical number-average molar mass calculated with ([DEM + DOM]_0_/[ETSB]_0_) × (*M*_W_ of DEM, 198.22 g mol^−1^ and *M*_W_ of DOM, 366.54 g mol^−1^) + chain ends (–C_6_H_9_O_2_ and –H; 114.1 g mol^−1^).

c Number-average molar mass measured by SEC with PMMA calibration.

d Dispersity value as determined by SEC with PMMA calibration.

**Fig. 1:**
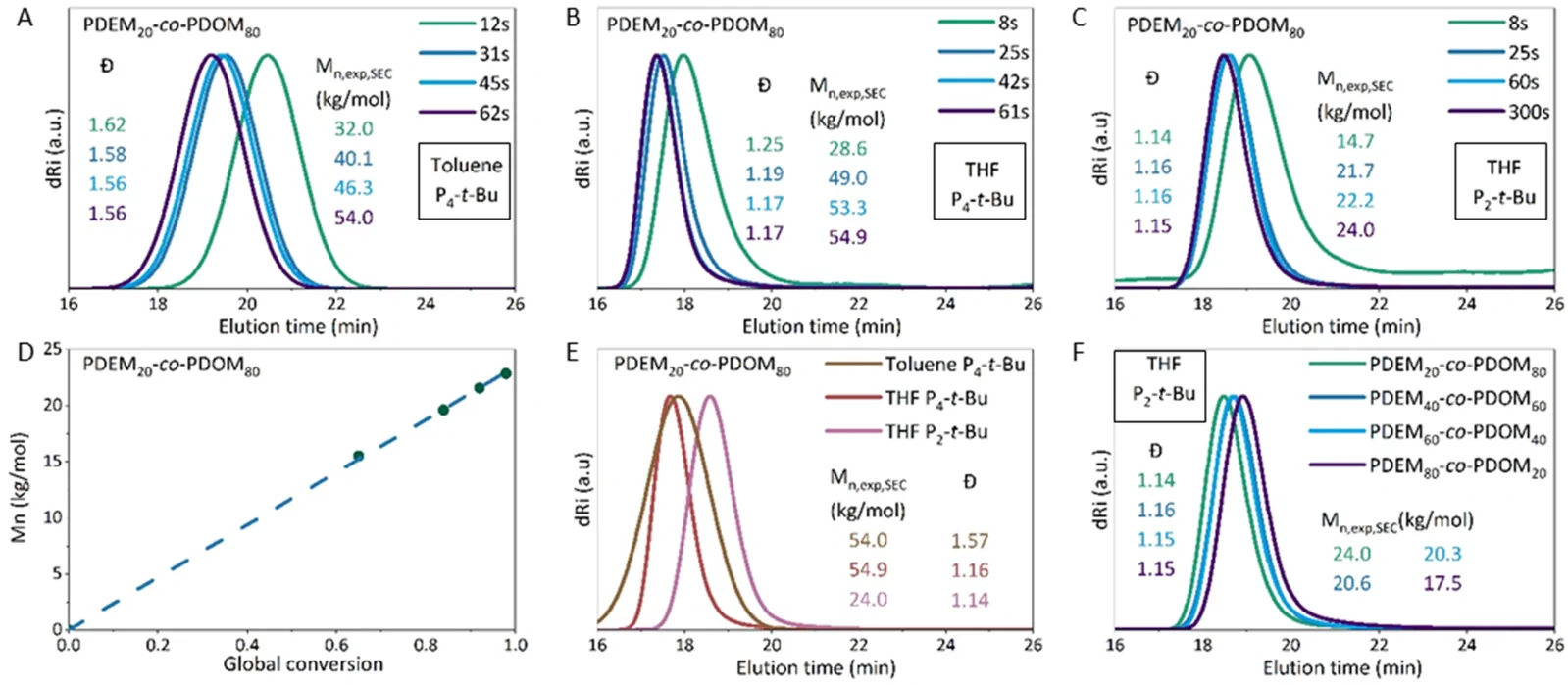
(A) SEC traces at different reaction times for the O-GTcP of DEM and DOM, for the specific composition PDEM_20_-*co*-PDOM_80_ in toluene as solvent and P_4_-*t*-Bu as catalyst (Table 1, entry 1). (B) SEC traces at different reaction times for the copolymerization of DEM and DOM, for the specific composition PDEM_20_-*co*-PDOM_80_ in THF as solvent and P_4_-*t*-Bu as catalyst (Table 1, entry 5). (C) SEC traces at different reaction times for the copolymerization of DEM and DOM, for the specific composition PDEM_20_-*co*-PDOM_80_ in THF as solvent and P_2_-*t*-Bu as catalyst (Table 1, entry 9). (D) Plot of *M*_n_ obtained by SEC *vs.* time for PDEM_20_-co-PDOM_80_ (Table 1, entry 9) see also Fig. S6. (E) SEC traces comparison between polymerization carried out using P_2_-*t*-Bu (pink) and P_4_-*t-*Bu (red) as catalyst in THF and P_4_-*t*-Bu in toluene (brown) showing a shift to lower molar mass when using THF and P_2_-*t*-Bu (Table 1, entries 1, 5, 9). (F) SEC traces comparison of copolymers obtained in THF and using P_2_-*t*-Bu as catalyst (Table 1, entries 9–12) showing a low dispersity (<1.16). See also Fig. S6.

We next evaluated the influence of solvent polarity (toluene *vs.* THF) on the level of control achieved in P_4_-*t*-Bu-mediated O-GTcP of DEM and DOM. Comparable reaction rates were observed in both media (Fig. S2 to S4), indicating that the change in solvent does not strongly affect the overall propagation kinetics under these conditions. However, a clear difference in molar mass distribution was evident: copolymers prepared in THF exhibited lower dispersity values, Fig. 1B (*Đ* < 1.30, Fig. S5) than those obtained in toluene (*Đ* = 1.56–1.60, Fig. S1). This improved control in THF is consistent with more efficient stabilization and exchange of ionic siliconate intermediates in the more polar solvent, thereby favoring a better-balanced activation-deactivation process (Scheme S1).^15^

In contrast, stronger ion pairing in the less polar toluene medium may reduce the efficiency of this exchange and contribute to the broader molar-mass distributions. The P_4_-*t*-Bu-mediated copolymerizations also afforded experimental number-average molar masses (*M*_n_) higher than the theoretical values. This behavior was observed previously for dialkyl muconate O-GTP in both THF and toluene,^14^ suggesting that the effective number of chains contributing to polymer growth is lower than that calculated from the nominal ETSB concentration. This may be due to the formation of inactive initiator-derived species under these highly basic and rapidly propagating conditions.

To further improve control over O-GTcP the less basic phosphazene P_2_-*t*-Bu was investigated as catalyst in both THF and toluene (Table 1, entries 9–14). In GTP processes, the balance between propagation and reversible deactivation strongly depends on catalyst basicity, which influences the exchange dynamics of the siliconate intermediates (Scheme S1).^19,22,25^ Accordingly, reducing catalyst basicity was expected to moderate the highly rapid polymerization and promote a more balanced activation-deactivation regime during chain growth. In THF, O-GTcP mediated by P_2_-*t*-Bu remained rapid, although a higher catalyst loading (10 mol% relative to initiator) was required compared to P_4_-*t*-Bu (1 mol%). Under these conditions, near-complete monomer conversion was still achieved within minutes, while improved control over molar mass was obtained (Fig. 1C and D). In particular, the experimental *M*_n_ values proved lower than those obtained with P_4_-*t*-Bu, by a factor of approximately 2.3, and were closer to the targeted values (Fig. 1E). Narrow molar mass distributions were maintained across all copolymer compositions investigated (*Đ* < 1.30, Fig. 1F and S6). The *M*_n_ values obtained for these relatively low-molar-mass polymers should nevertheless be interpreted with consideration of the limitations associated with absolute molar-mass determination using the multi-angle laser light scattering (MALLS) detector and with conventional SEC calibration using PMMA standards. As discussed below, MALDI-ToF MS analysis of low-molar-mass samples further confirms the expected ETSB-derived chain ends and provides no evidence for a side population arising from an alternative initiation pathway.

By contrast, no measurable monomer conversion was observed when P_2_-*t*-Bu was employed in toluene, highlighting the combined importance of catalyst basicity and solvent for efficient activation of the O-GTcP process. The more polar THF medium likely facilitates the exchange of ionic siliconate intermediates, thereby enabling controlled propagation with the less basic phosphazene catalyst (Scheme S1).^15^

In order to evaluate sequence distribution, time-resolved ^1^H NMR spectroscopy was employed to monitor monomer consumption. Owing to the rapid kinetics of O-GTcP in both solvents, aliquots were withdrawn at very short time intervals (≈10 s), quenched with benzoic acid, and analysed by NMR and SEC. Overall monomer conversion was determined from the relative integration of residual vinyl protons and backbone olefinic signals (Fig. 2 and S7–S18). Individual monomer conversions were extracted from characteristic methylene resonances in the α-position to oxygen for DEM and DOM, enabling independent tracking of each species. Across a broad range of initial DEM molar fractions (0.2–0.8), both monomers were consumed concomitantly, with near-quantitative conversion reached within approximately 40 s (Fig. S2–S4). Individual monomer conversions were determined from ^1^H NMR analysis using a system of equations (eqn (S1)–(S4)), enabling calculation of the instantaneous copolymer composition (*F*_DEM_) throughout the reaction.

**Fig. 2:**
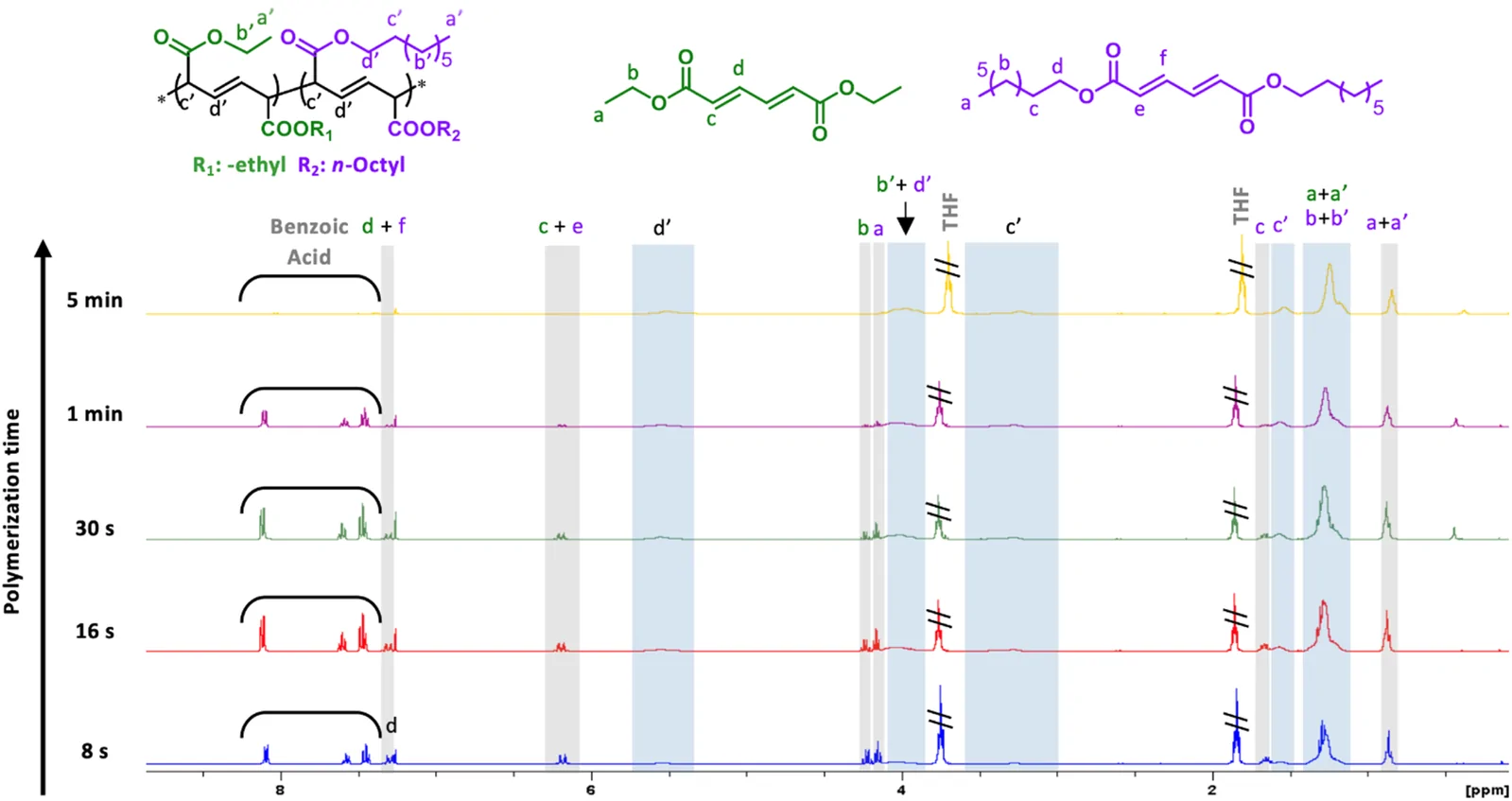
Monitoring by ^1^H NMR of one-pot copolymerization of DEM (green) and DOM (purple), showing the changes in the characteristic peaks of the monomer after the start of the polymerization: 8 s (blue spectrum), 16 s (red spectrum), 30 s (green spectrum), 1 min (purple spectrum) and 5 min (yellow spectrum).

Importantly, DEM and DOM exhibited comparable consumption profiles over the full composition range, indicating the absence of strong preferential incorporation. To quantify copolymer microstructure, reactivity ratios were then determined using the integrated Mayo–Lewis equation within the terminal model framework. Because of the exceptionally fast kinetics of O-GTcP, accessing sufficiently low conversion regimes for reliable Kelen–Tüdös analysis proved experimentally challenging (Fig. S19).^42,43^ Reactivity ratios were therefore extracted using the Meyer–Lowry method,^44^ which provided excellent agreement between experimental and fitted copolymer compositions (Fig. 3A and B). The resulting values, *r*_DEM_ = 1.08 ± 0.26 and *r*_DOM_ = 0.84 ± 0.24, indicate only a modest preference for DEM self-propagation with both values close to unity within the confidence intervals obtained from the fitting. The copolymerization can therefore be described as predominantly statistical, without evidence for strong preferential incorporation of either comonomer.

**Fig. 3:**
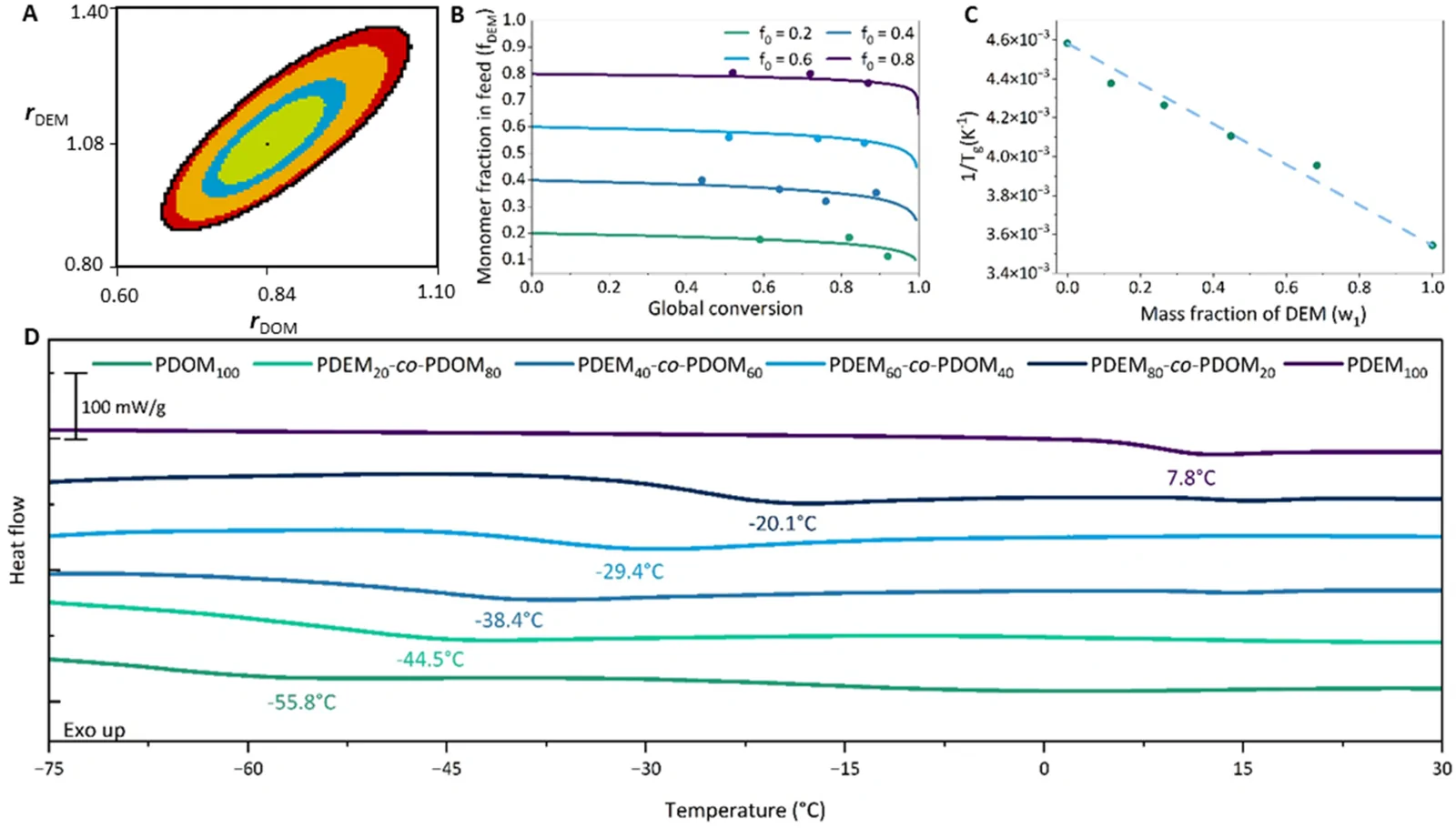
(A) 95% joint confidence interval (JCI) for reactivity ratios in THF and using P_2_-*t*-Bu as catalyst, *r*_DEM_ = 1.08 ± 0.26 and *r*_DOM_ = 0.84 ± 0.24, coloured regions represent 50%, 70% and 90% JCIs. (B) Prediction of the comonomer ratio (solid lines) as a function of conversion *vs.* experimental points (solid dots), for *f*_DEM,0_ = 0.80, 0.60, 0.40 and 0.20 (top to bottom respectively) for the P_2_-*t*-Bu/THF system. (C) Glass transition temperatures of the copolymers predicted using the Fox equation (solid line) and experimentally determined by DSC (solid dots) (D) DSC thermograms (second heating run) showing an increase *T*_g_ value upon increasing the DEM content.

The resulting copolymer microstructure was further investigated by thermal analysis. Differential scanning calorimetry (DSC) revealed a single glass transition temperature (*T*_g_) for all poly(DEM-*co*-DOM) samples (Fig. 3C), consistent with a single thermally homogeneous phase and the absence of detectable phase separation. Moreover, *T*_g_ decreased progressively with increasing DOM content (Fig. 3D), reflecting the enhanced chain flexibility imparted by the octyl side chains. To further examine the relationship between copolymer composition and thermal properties, the experimental *T*_g_ values were compared with those predicted by the Fox equation.^45^ Excellent agreement was observed across the entire composition range. For instance, the equimolar copolymer PDEM_50_-*co*-PDOM_50_ exhibited an experimental *T*_g_ of −39.5 °C, in close agreement with the calculated value of −39 °C. The single *T*_g_ values and their agreement with the Fox prediction are consistent further support the predominantly statistical nature of the copolymers.

Taken together, the kinetic analysis, reactivity ratio determination, and thermal characterization demonstrate that O-GTcP affords predominantly statistical copolymuconates with composition-dependent and predictable thermomechanical properties. Despite substantial differences in copolymerization control between the P_4_-*t*-Bu/toluene and P_2_-*t*-Bu/THF systems, the overall copolymerization behavior remained essentially unchanged (Fig. S20 and S21). Reactivity ratios determined under the more controlled P_2_-*t*-Bu/THF conditions remained close to unity and comparable to those obtained with P_4_-*t*-Bu, with only a modest increase in *r*_DEM_, indicating slightly enhanced DEM self-propagation under these conditions (Table S1).

To further probe copolymer microstructure, a low-molar-mass sample (DP = 10) prepared by O-GTcP using P_2_-*t*-Bu in THF was analyzed by matrix-assisted laser desorption/ionization time-of-flight mass spectrometry (MALDI-ToF MS). The mass spectrum (Fig. 4) displayed a single family of well-resolved peaks corresponding to copolymer chains incorporating both DEM and DOM repeating units, together with the expected ETSB-derived initiating fragment and proton-terminated chain end resulting from the quenching procedure. Importantly, no separate distributions attributable to DEM- or DOM-rich homopolymers were detected. Instead, the observed mixed-chain population is consistent with the incorporation of both comonomers within individual copolymer chains, in agreement with the statistical copolymerization behavior inferred from reactivity ratio analysis. For example, the signal at *m*/*z* = 2763.5 Da can be assigned to a copolymer containing four DEM units (*M* = 198.2 g mol^−1^) and five DOM units (*M* = 366.6 g mol^−1^), together with the ETSB-derived chain end (*M* = 113.3 g mol^−1^) and proton termination. Similar assignments could be made throughout the spectrum, confirming the expected composition and chain-end structures of the copolymuconates. The MALDI-ToF analysis therefore provides direct molecular-level evidence for the formation of statistical poly(DEM-*co*-DOM) compounds by O-GTcP.

**Fig. 4:**
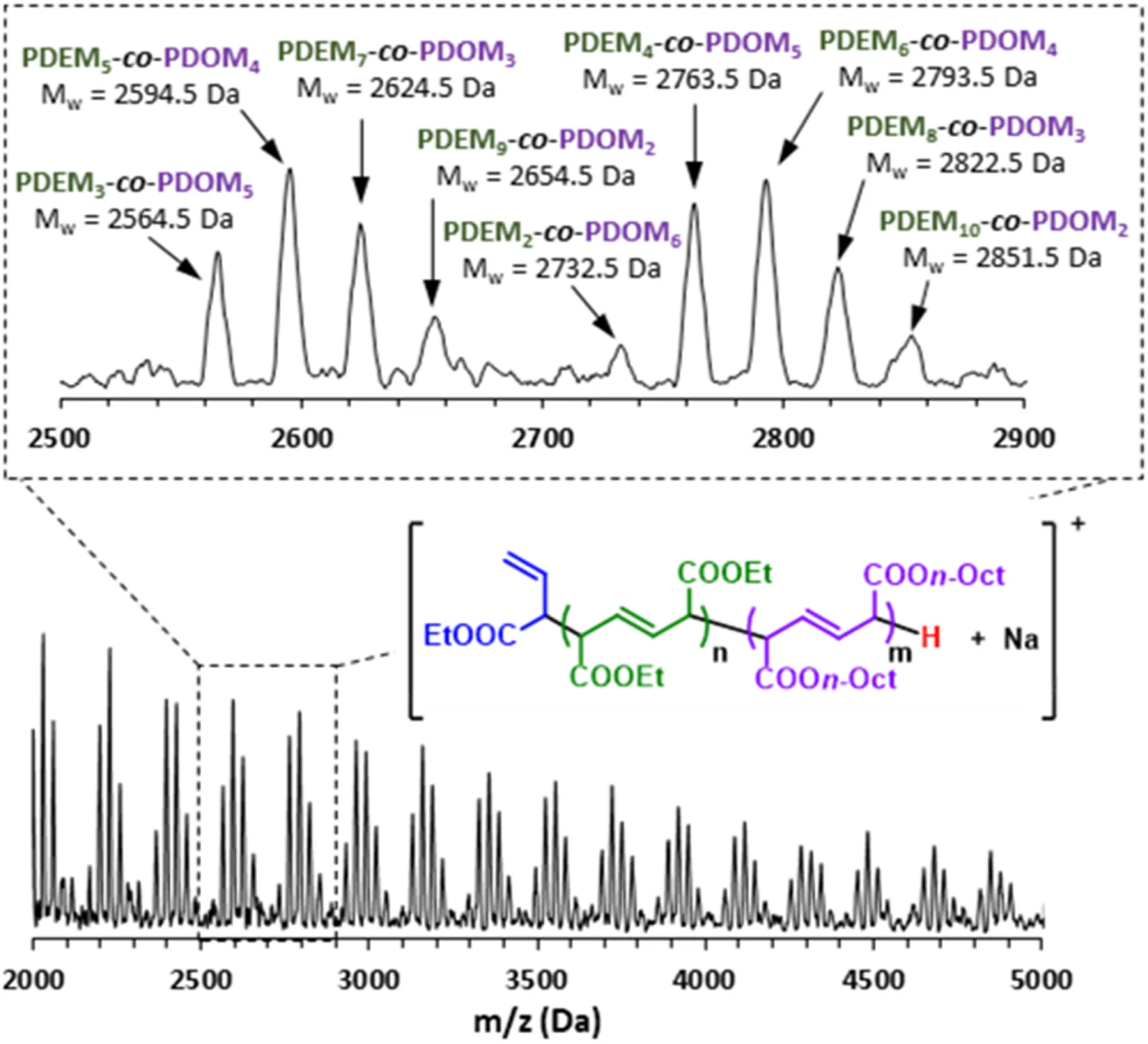
MALDI-TOF MS of PDEM_5_-*co*-PDOM_5_ synthetized with P_2_-*t-*Bu catalysed O-GTcP.

### 2.2 O-GTcPISA in isododecane as nonpolar media

The preceding results establish that O-GTcP of DEM and DOM affords predominantly statistical copolymuconates with composition-dependent properties in homogeneous media. We next investigated whether this copolymerization strategy could be leveraged to direct self-assembly during copolymerization in a selective nonpolar medium. In most established PISA systems, particularly those based on controlled radical polymerization, nanoparticle formation relies on sequential synthesis of amphiphilic block copolymers, in which a soluble stabilizing block is first prepared before chain extension with an insoluble segment triggers self-assembly.^26–30^ More recently, O-GTP was adapted to PISA processes in *n*-heptane as nonpolar medium (GTPISA), enabling the preparation of self-stabilized methacrylic nanoparticles through sequential block copolymer formation.^46–48^ In contrast, the DEM/DOM system provides a fundamentally different scenario. Whereas poly(DOM) remains soluble in nonpolar solvents owing to its long octyl side chains, increasing incorporation of DEM units is expected to progressively decrease the compatibility of the copolymer with the nonpolar medium. We therefore hypothesized that statistical copolymer growth in isododecane could progressively decrease copolymer solubility, and ultimately trigger self-assembly during polymerization, *i.e.*, without requiring a predesigned block copolymer or a sequential polymerization strategy. Isododecane was selected as the reaction medium owing to its nonpolar character and its widespread use as a formulation solvent in industrial and cosmetic applications.^49^ Under these conditions, the behavior of the DOM/DEM system proved to be strongly dependent on copolymer composition (Fig. 5A). Up to equimolar feed ratios, one-pot O-GTcP remained homogeneous when targeting a total degree of polymerization of 100, consistent with sufficient solubilization of the growing copolymers by the DOM-rich units. ^1^H NMR spectroscopy confirmed near-quantitative conversion of both monomers within 10 min under these conditions. SEC analyses further revealed number-average molar masses in reasonable agreement with the targeted values based on the initial [DEM + DOM]_0_/[ETSB]_0_ ratio, while dispersity values remained relatively high (*Đ* = 1.50–1.57), consistent with our previous observations for highly rapid O-GTcP in low-polarity media (Fig. 5C and S22).^14^ As the DEM content increased and the system approached the composition range in which self-assembly occurred, the SEC traces became progressively broader and, for the PDEM_60_-*co*-PDOM_40_ sample, exhibited a discernible shoulder (Fig. 5C). This feature coincides with the transition from homogeneous copolymerization to particle formation and may reflect the increasingly heterogeneous environment of growing chains as self-assembly develops. Importantly, the absence of an abrupt change in the monomer–consumption profiles at the onset of turbidity argues against a distinct change in copolymerization mechanism or to a competing copolymerization pathway. The shoulder is thus tentatively associated with the solvation environment accompanying the transition into the PISA regime.

**Fig. 5:**
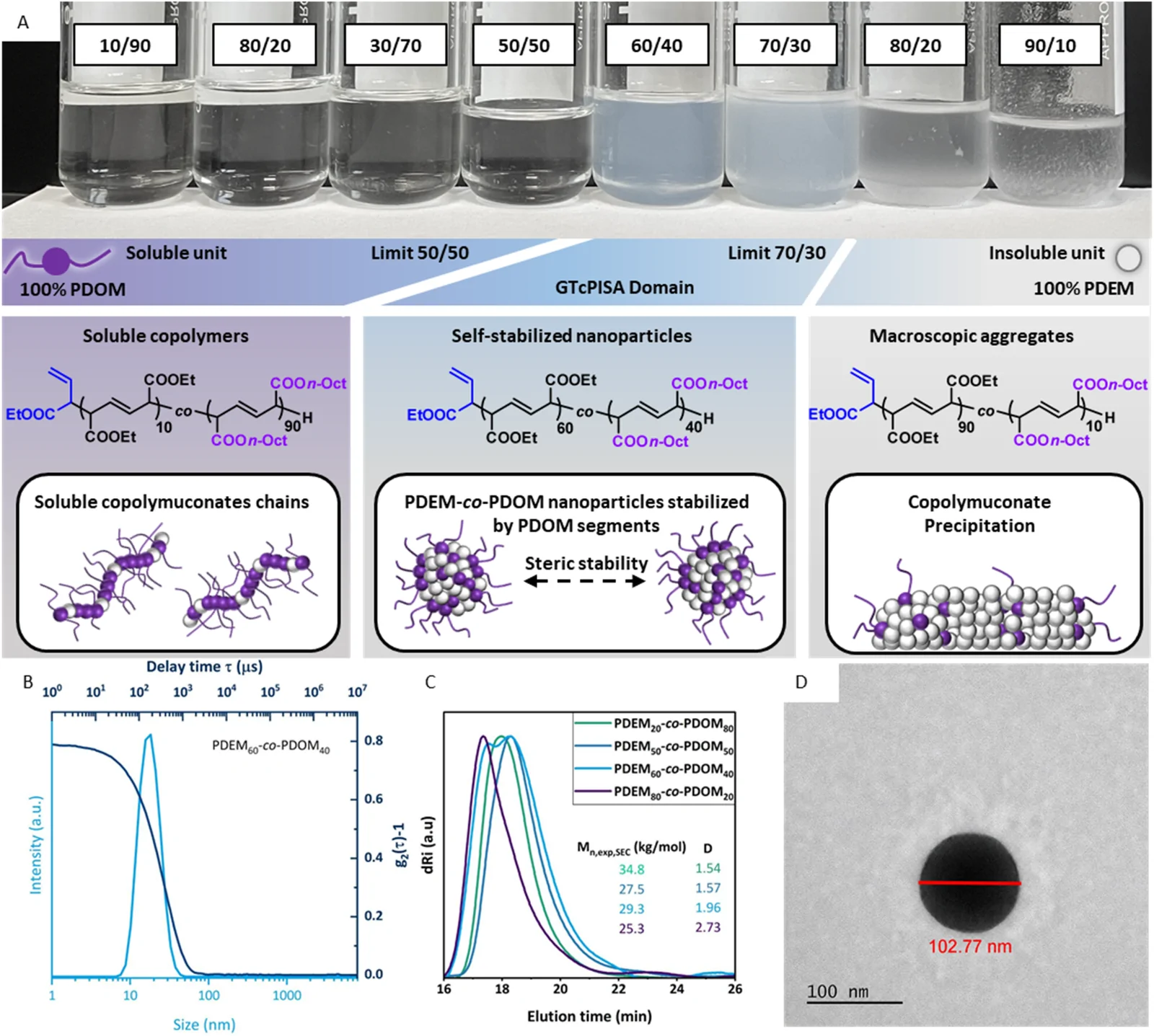
(A) Effect of the molar proportion of DEM and DOM units on statistical copolymers synthesized *via* O-GTcPISA in isododecane using P_4_-*t*-Bu as catalyst and ETSB as initiator (Table 2, entries 1–9) (B) DLS size distribution showing the hydrodynamic diameter as a function of scattered intensity (cyan) and the corresponding correlation function (dark blue) for PDEM_60_-*co*-PDOM_40_ nanoparticles (see also Fig. S29–S30). (C) SEC traces of PDEM-*co*-PDOM copolymers at different monomer compositions (Table 2, entries 2, 4, 5, 8, see Fig. S22 for the rest) of DEM and DOM copolymerization using P_4_-*t*-Bu catalyst in isododecane. (D) TEM image showing a spherical particle of copolymer PDEM_60_*-co*-PDOM_40_ after staining for 30 minutes with OsO_4_.

To assess whether the change from THF or toluene to isododecane altered the relative reactivity of the two comonomers, the reactivity ratios of DEM and DOM were determined in the latter solvent. Despite the experimental challenges associated with kinetic monitoring in this highly nonpolar medium, fitting of the conversion-dependent composition data gave *r*_DEM_ = 1.89 ± 0.93, *r*_DOM_ = 0.58 ± 0.28 (Table S1 and Fig. S23–S27). Compared with the values obtained in THF and toluene, these results indicate a modest increase in the tendency of DEM to undergo self-propagation, whereas DOM displays a comparatively weaker preference for self-incorporation. Importantly, the reactivity ratios remain well below the regime expected for strongly gradient-forming copolymerizations. The resulting sequence distribution should therefore remain predominantly statistical, although with a modest enrichment of consecutive DEM units (Fig. S28A). The kinetic data further indicate that self-assembly does not introduce a discernible change in the overall copolymerization behavior. Similar monomer–consumption profiles were indeed obtained for the different initial feed compositions, including those that remained homogeneous and those for which turbidity developed during copolymerization (Fig. S26). Moreover, the evolution of the instantaneous DEM fraction with increasing global conversion follows the behavior expected from the experimentally determined reactivity ratios, with no abrupt deviation coincident with the onset of turbidity (Fig. S28C). Thus, the progressive changes in copolymer composition observed during O-GTcPISA appear consistent with the intrinsic copolymerization behavior of DEM and DOM, rather than with a distinct change in monomer consumption associated with self-assembly. These observations support a scenario in which nanoparticle formation develops progressively as the growing copolymer becomes increasingly incompatible with the reaction medium.

A pronounced change in the macroscopic behavior was observed when the DEM content exceeded 60 mol%. In this regime, turbidity progressively developed during copolymerization, indicating the onset of phase separation concomitant with copolymer growth, ultimately leading to stable milky dispersions. Dynamic light scattering (DLS) measurements revealed particle hydrodynamic diameters around 20 nm (Fig. 5B, S29 and S30), consistent with the formation of nanoscale spherical particles in dispersion. Transmission electron microscopy (TEM) further revealed spherical objects with lateral dimensions of approximately 100 nm for poly(DEM_60_-*co*-DOM_40_) prepared in isododecane (Fig. 5D). This apparent discrepancy can be rationalized by the different states of the samples during characterization. DLS probes particles dispersed in solution, whereas TEM imaging is performed after deposition and drying, during which soft polymeric particles can undergo deformation. This effect is expected to be particularly pronounced for these low-*T*_g_ copolymers (*T*_g_ = −55 to −20 °C), which remain deformable under the deposition and drying conditions. Consistent with this interpretation, AFM measurements revealed objects with lateral dimensions of approximately 100 nm but heights of only 2–5 nm (Fig. S31), indicating that the particles undergo significant flattening upon deposition. The larger lateral dimensions observed by TEM and AFM therefore most likely result from spreading and deformation of the particles during sample preparation, although aggregation of multiple particles cannot be completely excluded.

The dependence of self-assembly on chain length was subsequently examined by targeting DP = 200. Interestingly, the copolymer containing 20 mol% DEM remained fully soluble, indicating that DOM-rich composition retains sufficient compatibility with isododecane even at the increased chain length. In contrast, the equimolar copolymer, which remained homogeneous at DP = 100, underwent self-assembly at DP = 200, producing stable nanoparticles with an average hydrodynamic diameter of 96 nm, as determined by DLS (Fig. S30). These results demonstrate that nanoparticle formation is governed by the combined effects of composition and chain length. Self-assembly therefore occurs once the growing statistical copolymer reaches a sufficiently high level of incompatibility with the nonpolar medium, which can be achieved either by increasing the proportion of DEM units at a given chain length or by increasing the chain length at a given composition. This behavior differs fundamentally from previously reported GTPISA systems relying on sequential block copolymer synthesis.^30–32^ In the present case, nanoparticle formation emerges directly during one-pot copolymerization, as the growing copolymer progressively crosses a composition- and chain-length-dependent solubility threshold. This organocatalyzed group transfer copolymerization-induced self-assembly (O-GTcPISA) process thus establishes a distinct PISA regime in which nanoscale self-assembly is generated without predesigned block copolymer architecture or sequential polymerization.

### 2.3 Closed-loop thermal recycling of statistical copolymuconates

An important feature of polymuconates is the presence of intrinsically weakened C–C bond along the polymer backbone, which enables thermally induced depolymerization and recovery of the corresponding monomers.^11–13,15,50,51^ O-GTcP affords well-defined copolymuconates with near-quantitative monomer conversion and controlled molar masses within minutes under mild conditions. Importantly, copolymerization proceeds through selective 1,6-conjugate addition to generate 1,4-linked repeating units,^15^ as supported by the spectroscopic analysis (for detailed discussion about the selectivity of the process, see Schemes S2 and S3). To evaluate the closed-loop recyclability of our statistical copolymuconates, thermal depolymerization of the PDEM_50_-*co*-PDOM_50_ sample was investigated in diphenyl ether (DPE) at 260 °C, under conditions favoring the monomer side of the monomer-polymer equilibrium. From a thermodynamic perspective, the extent of depolymerization is governed by the ceiling temperature, which depends on the polymerization equilibrium and experimental conditions. The depolymerization process was monitored by ^1^H NMR spectroscopy (Fig. 6). After 20 min, copolymer signals completely disappeared and were replaced by resonances corresponding to the regenerated muconate monomers (Fig. S34). Further analysis by SEC of the crude depolymerization mixture prior to monomer purification, provided no evidence for detectable residual high-molar-mass copolymer chains (Fig. S40). The absence of polymer resonances was also confirmed by ^1^H NMR spectroscopy. Following removal of DPE and purification, both DEM and DOM were recovered, corresponding to 42% mol and 58% mol, respectively. Analysis of the as-recovered monomer mixture revealed the presence of both *trans*,*trans-* and *cis*,*trans-*muconate isomers (75% and 25% respectively for DEM and 78% and 23% respectively for DOM), consistent with thermal isomerization of the conjugated double bonds occurring during the depolymerization conditions (Fig. 6, S35 and S36).^11,13,15^ The molecular-level origin of the high depolymerizability of polymuconates can be rationalized by the thermally promoted cleavage of weakened C–C bonds within their backbone. Recent computational analysis of thermally depolymerizable polydiene systems has shown that the energetic cost of such bond cleavage is strongly influenced by the stabilization of the resulting radical species by the conjugated unsaturated backbone.^51^ In the present system, cleavage of such activated bonds could consequently initiate successive backbone-cleavage and monomer release, consistent with an unzipping-type depolymerization pathway (see the proposed mechanism in Scheme S4). To evaluate whether the regenerated material could be directly returned to the polymerization cycle, the recovered monomer mixture was dried after separation from DPE, and subsequently subjected to O-GTcP without purification of the individual muconate geometrical isomers. The resulting copolymer displayed a SEC trace that closely overlapped that of the original PDEM_50_-*co*-PDOM_50_ material (Fig. S37, S38 and S40), demonstrating the successful regeneration of comparable molar-mass characteristics from the recycled feed. Thus, the copolymuconate can undergo a polymer–monomer–polymer cycle without requiring purification or separation of the regenerated DEM and DOM geometrical isomers. These results establish the closed-loop chemical recyclability, of the statistical copolymuconates prepared by O-GTcP. The ability to directly repolymerize the thermally regenerated feed, despite the formation of multiple muconate geometrical isomers, further highlights the robustness of the recycling process.

**Fig. 6:**
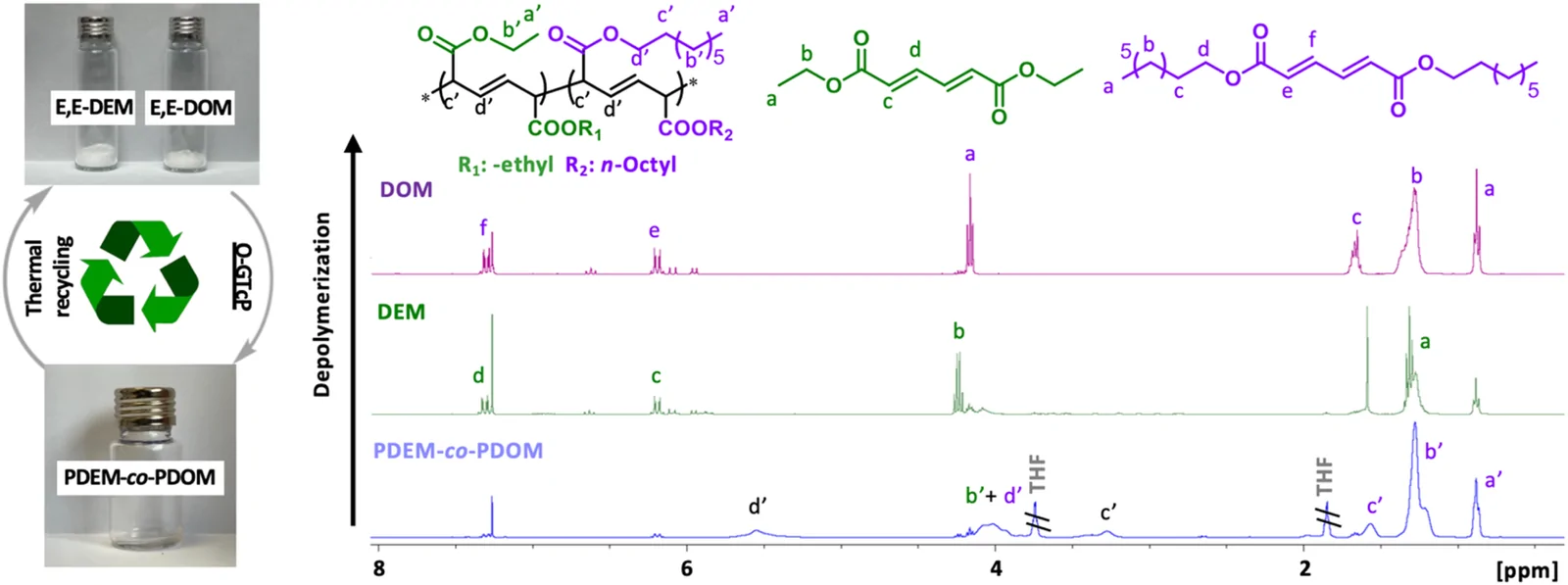
Closed-loop recycling of PDEM_50_-*co*-PDOM_50_, synthesized by GTP with P_2_-*t*-Bu catalyst and thermally recycled at 260 °C in diphenyl ether as solvent. ^1^H NMR spectra of recycled monomers DOM (purple), DEM (green) after thermal recycling of copolymer (blue).

## 3 Conclusion

This work establishes O-GTcP of dialkyl muconate esters as a versatile and solvent-programmable platform that unifies precision copolymerization, self-assembly, and chemical recyclability. Through the O-GTcP of DOM and DEM, predominantly statistical copolymuconates with controlled molar masses, tunable thermal properties, and, under optimized conditions, narrow dispersities were obtained under homogeneous conditions, while maintaining efficient depolymerization back to the parent monomers. Importantly, the regenerated monomer mixture could be directly repolymerized without purification, thereby demonstrating a polymer–monomer–polymer cycle. Altering the reaction medium from non-selective solvents, like toluene and THF, to selective isododecane enabled a transition from molecularly dissolved statistical copolymers to spontaneously formed nanostructured dispersions, giving rise to the first demonstration of O-GTcPISA. In this regime, self-assembly emerges progressively from the combined effects of copolymer composition and chain length. These findings reveal how the interplay between statistical copolymer composition and solvent selectivity can direct nanoscale organization without the need for surfactants, block copolymer architectures, or multistep processing. More broadly, this study highlights the potential of renewable muconate monomers and metal-free organocatalysis to deliver a polymerization platform that integrate sustainable feedstocks, closed-loop recyclability, and advanced materials design.

## 4 Experimental

### 4.1 Materials

Detailed information on the materials used in this study is provided in the SI.

### 4.2 Methods

Detailed information on the characterization techniques and equipment used in this study is provided in the SI.

## Author contributions

J. D. E. S. and E. D. performed the experimental work. J. D. E. S., E. D and T. D. carried out the data analysis. The work and the underlying concepts were developed under the supervision of D. T. The manuscript was written with contributions from all authors. All authors have approved the final version of the manuscript. Paul Marque and Benjamin Cabannes-Boué are gratefully acknowledged for their assistance with DLS and AFM experiments.

## Conflicts of interest

There are no conflicts to declare.

## Supplementary Material

SC-OLF-D6SC05973B-s001

## Data Availability

Supplementary information (SI): all experimental procedures, materials, analysis and spectroscopic data, including SEC traces and NMR. See DOI: https://doi.org/10.1039/d6sc05973b.
