# Supplementary material for: Solvent-programmable organocatalyzed group transfer copolymerization (O-GTcP) of muconate esters: from statistical control to copolymerization-induced self-assembly
Source: Chem Sci. 2026 Oct 1. Online ahead of print. doi: 10.1039/d6sc05973b (PMC13643524; doi:10.1039/d6sc05973b)
Supplement: Supplementary file 1 [file SC-OLF-D6SC05973B-s001.pdf]

Supplementary Information (SI) for Chemical Science.
This journal is © The Royal Society of Chemistry

Supporting Information for

Solvent-Programmable Organocatalyzed Group Transfer Copolymerization (O-GTcP) of Muconate Esters: From Statistical Control to Copolymerization-Induced Self-Assembly.

José-David Estrada Sotomayor,[a] Émilie Diomar,[a] Thomas Dardé,[a] Xavier Schultze,[b] Guillaume Fleury,[a] and Daniel Taton[a]

[a.] Univ. Bordeaux, CNRS, Bordeaux INP, LCPO, UMR 5629, F-33600, Pessac, France

[b.] L'Oréal Research & Innovation, 1 Avenue Eugène Schueller, 93600, Aulnay Sous-Bois, France

Table of Content

Materials: 4

Instrumentation: 4

Nuclear magnetic resonance (NMR) spectroscopy 4

Size exclusion chromatography (SEC). 5

Differential scanning calorimetry (DSC). 5

Thermogravimetric analysis (TGA) 5

Matrix assisted laser desorption ionization – time of flight (MALDI-ToF) 6

Synthesis: 7

Synthesis of 1-ethoxy-1-(trimethylsiloxy)-1,3-butadiene (ETSB). 7

Monomer synthesis: 7

Synthesis of (E,E) diethyl muconate (E,E-DEM) 7

Synthesis of (E,E) di-n-octyl muconate (E,E-DOM) 8

Group transfer copolymerization (GTcP) of trans,trans-dialkyl muconates: 9

Synthesis of $P(E,E\text{-}DEM)_{20}$-co-$P(E,E\text{-}DOM)_{80}$ in toluene with P_4-*t*-Bu catalyst 9

Synthesis of $P(E,E\text{-}DEM)_{20}$-co-$P(E,E\text{-}DOM)_{80}$ in THF with 9

Synthesis of $P(E,E\text{-}DEM)_{60}$-co-$P(E,E\text{-}DOM)_{40}$ in isododecane with 10

Depolymerization of $PDEM_{50}$-co-$PDOM_{50}$ 11

O-GTcP dissociative mechanism: 12

Regioselectivity and geometrical selectivity of muconate O-GTP: 13

O-GTcP monitoring: 16

Determination of reactivity ratios during O-GTcP: 26

DSC copolymer characterization: 40

O-GTcP using isododecane as solvent: 44

Thermal depolymerization of PDEM-co-PDOM: 54

^{1}H NMR of dialkyl muconates: 64

^{13}C NMR of dialkyl muconates: 66

MALS analysis and dn/dc determination: 68

^{1}H NMR of copoly(dialkyl muconate)s: 71

^{13}C NMR of copoly(dialkyl muconate)s: 75

Materials:

Toluene (>99.9%, VWR) was dried on butyl lithium/styrene and purified by cryo-distillation. Tetrahydrofuran (THF, >99.9%, Thermo Scientific) was dried and purified by inhibitor on sodium/benzophenone and by cryo-distillation. Isododecane (80%, FisherScientific) was dried using CaH_2 and purified by a cryo-distillation. Phosphazene base P_4-*t*-Bu solution (0.8 M in hexane, Sigma Aldrich), phosphazene base P_2-*t*-Bu solution (2M in THF, Sigma Aldrich), and 1-ethoxy-1-(trimethylsiloxy)-1,3-butadiene (ETSB) were placed under inert atmosphere (argon) and stored in the glovebox. Ethyl but-3-enoate (98%, BLD Pharm), trans,trans-muconic acid (98%, Sigma Aldrich), cis cis-muconic acid (>97%, Sigma Aldrich), chlorotrimethyl silane (98%, Thermo Scientific Chemicals), methanol (>99.8%, VWR), ethanol (>99.8%, VWR), 1-octanol (>99%, Thermo Scientific), methanol (>99.9%, VWR), dichloromethane (CH_2Cl_2, >99.9% Scharlau), *n*-pentane (>99%, Thermo Scientific) were used as received.

Instrumentation:

Nuclear magnetic resonance (NMR) spectroscopy.

^1H NMR and ^{13}C NMR spectra were recorded in deuterated solvents on Bruker Advance at 400 MHz at room temperature. Deuterated chloroform ($CDCl_3$), was used as solvent and the residual solvent signals were used as an internal reference. For ^1H NMR, $CDCl_3$ = 7.26 ppm. Monomer conversion was determined using ^1H NMR spectroscopy using the following system of equations, where characteristic proton signals of monomers and polymers were selected.

$$\int_{6}^{6.1} H = 2\, H_{DEM} + 2\, H_{DOM} \qquad (S1)$$

$$\int_{5.2}^{5.8} H = 2\, H_{PDEM} + 2\, H_{PDOM} \qquad (S2)$$

$$\int_{4.05}^{4.1} H = 4\, H_{DEM} \qquad (S3)$$

$$\int_{0.85}^{0.95} H = 6\, H_{DOM} + 6\, H_{PDOM} \qquad (S4)$$

After solving this system of equations, the global and specific monomer conversion were determined using the following equations.

$$X_g = \frac{\int_{5.2}^{5.8} H}{\int_{6}^{6.1} H + \int_{5.2}^{5.8} H} \quad (S5)$$

$$X_{DEM} = \frac{H_{PDEM}}{H_{DEM} + H_{PDEM}} \quad (S6)$$

$$X_{DEM} = \frac{H_{PDOM}}{H_{DOM} + H_{PDOM}} \quad (S7)$$

Size exclusion chromatography (SEC).

Polymer molar masses were determined by Size Exclusion Chromatography coupled to Multi-Angle Light Scattering (SEC-MALS) using tetrahydrofuran (THF) as the eluent. Measurements in THF were performed on an Ultimate 3000 system from Thermoscientific equipped with diode array detector (DAD). The system also includes a using a multi-angle laser light scattering (MALLS) detector Optilab t-Rex (Waters/Wyatt Technology, Santa Barbara) and Optilab differential refractive index detector dRI (Waters/Wyatt technology, Santa Barbara). Polymers were separated on three G2000, G3000 and G4000 TOSOH HXL columns (300 mm x 7.8 mm, 5 μm) (linear separation range from 1000 Da to 400 000 Da) at a flowrate of 1 mL/min. Columns temperature was held at 40 °C. Easivial kit of PMMA from Agilent was used as the standard (M_n from 500 to 1.5 MDa). Molar masses were determined by conventional calibration and flow fluctuation was corrected by using TCB (0.2% final volume/volume) as flow marker. Data generated were analyzed by using ASTRA 7.3.2. Molar masses were also determined from a MALLS detector online, using dn/dc values reported in Table S1. The dn/dc values were determined experimentally by direct sequentially injections on the refractometer.

Differential scanning calorimetry (DSC).

Differential scanning calorimetry (DSC) measurements were performed using a TA Instruments DSC Q250 LN2 apparatus with a heating and cooling ramp of 10 °C min^{-1}. Glass transition temperatures were determined using the TA Universal Analysis software.

Thermogravimetric analysis (TGA).

Thermo-gravimetric measurements (TGA) of polymers samples (≈ 10 mg) were performed on a TA Instruments Q500 from room temperature to 950 °C with a heating rate of 10 °C.min^{-1}. The analyses were conducted under nitrogen atmosphere in platinum pans.

Matrix assisted laser desorption ionization – time of flight (MALDI-ToF).

Matrix assisted laser desorption ionization mass spectrometry spectra were performed at CESAMO (ISM, Bordeaux, France) on a Autoflex max TOF mass spectrometer (Bruker Daltonics, Bremen, Germany) equipped with a frequency tripled Nd:YAG laser emitting at 355 nm (Smartbeam laser). Spectra were recorded in positive ionization using the reflectron mode with an accelerating voltage of 19 kV. Samples were dissolved in THF at 10 mg/ml. The Dihydroxybenzoic acid matrix solution was prepared by dissolving 10 mg in 1 ml of THF. A THF solution of cationization agent (NaI, 10 mg/ml) was also prepared. The solutions were combined in a 10:1:1 volume ratio of matrix to sample to cationization agent. One to two microliters of the obtained solution were deposited onto the sample target. A polyethylene glycol (PEG) was used for external calibration. Data acquisition was performed using Flex Control 3.4 and reprocessed using Flex Analysis 3.4 software packages (Bruker Daltonics, Bremen, Germany) in the range of m/z 200-9000

Synthesis:

The initiator (ETSB) was synthetized using ethyl 3-butenoate as precursor after an adaptation of previous work.[1] Monomer synthesis was performed following the protocol of a previous work.[1]

Synthesis of 1-ethoxy-1-(trimethylsiloxy)-1,3-butadiene (ETSB).

A dry 100 mL Schlenk flask containing a magnetic stirring bar, flushed with argon, was charged with 16.05 mL of 1.5 M LDA solution in THF/heptane/ethylbenzene (24.08 mmol, 174 mmol, 1.4 eq.) and 20 mL of dry THF. The mixture was cooled at -84 °C in an ethyl acetate/liquid nitrogen bath. 2 mL (17.2 mmol, 1 eq.) of dried ethyl 3-butenoate were then added to the mixture at a rate of 1-2 drops every 5 seconds. After 30 min, 3.5 mL (27.5 mmol, 1.6 eq.) of chlorotrimethyl silane were added in the flask. The mixture was then allowed to warm at room temperature while we observed the formation of a white precipitate. After 20 min, the Schlenk flask was transferred in a glovebox and the mixture was filtrated with 0.45 μm, PTFE, syringe filter. The filtrate was concentrated under vacuum to remove volatile products, mixed with hexane, filtrated and concentrated under vacuum again. The crude product was then purified by cryo-distillation under vacuum (bath temperature: 80 to 120 °C). The products, a mix of 65%Z and 35%E, were analyzed by ^{1}H and ^{13}C NMR. Yield = 67%.

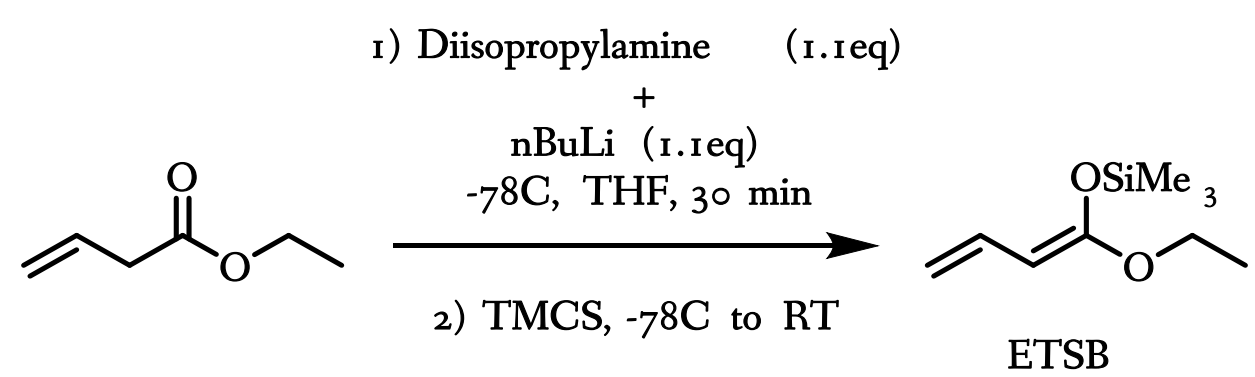

Monomer synthesis:

Synthesis of (E,E) diethyl muconate (E,E-DEM). Chloro-trimethylsilane (18.3 mL, 144 mmol, 4 eq.) was added to a solution of *trans,trans*-muconic acid (5.12 g, 36 mmol, 1 eq.) in ethanol (150 mL, in excess) in a 250 mL two-necked flask fitted with a condenser. The mixture was heated to 80 °C for 3 h. The reaction mixture was then cooled to room temperature. The progress of the reaction was monitored by ^{1}H NMR spectroscopy. After complete conversion, the crude product was processed through a series of purification steps. A saturated solution

of sodium bicarbonate ($NaHCO_3$, 70 mL) was slowly added to the flask. The mixture was stirred for 10 min, then transferred to a 500 mL separatory funnel, and 70 mL diethyl ether were added. The organic phase was preserved and the aqueous phase was extracted twice with diethyl ether (Et_2O, 70 mL). The organic phases were combined and washed with a saturated sodium chloride solution (NaCl, 70 mL), then dried over magnesium sulfate, filtered through cotton and concentrated under reduced pressure in a flask. The solid obtained was recrystallized three times in cold methanol. The diethyl muconate crystals were placed in a Schlenk flask with a magnetic stirring bar and the product was dried by azeotropic distillation in toluene (2 mL) for 30 min two times. A final azeotropic distillation with toluene (2 mL) was carried out overnight. The product was kept under argon. Yield: > 95%.

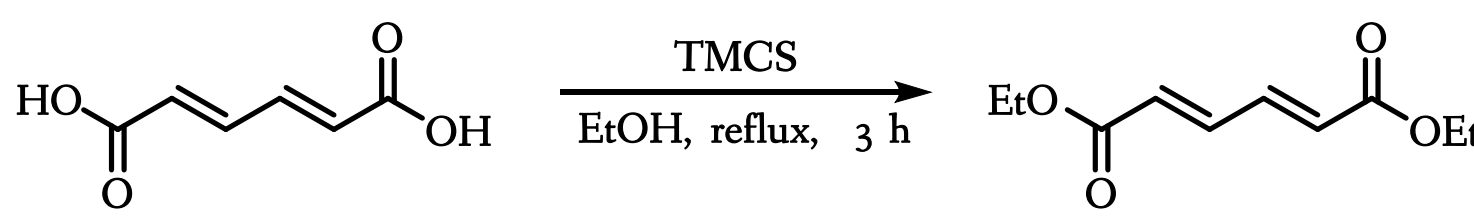

Synthesis of (E,E) di-n-octyl muconate (E,E-DOM). Chloro-trimethylsilane (18.3 mL, 144 mmol, 4 eq.) was added to a solution of *trans,trans*-muconic acid (5.12 g, 36 mmol, 1 eq.) in 1-octanol (150 mL, in excess) in a 250 mL two-necked flask fitted with a condenser. The mixture was heated to 100 °C for 3 h, then cooled to room temperature. The progress of the reaction was monitored by ^1H NMR. Once conversion was complete, the crude product was processed through a series of purification steps. The mixture was cooled in an ice bath and filtered through sintered glass. The solid residue was dissolved in 50 mL of diethyl ether and a saturated solution of sodium bicarbonate ($NaHCO_3$, 70 mL) was slowly added to the flask. The mixture was stirred for 10 min, then transferred to a 500 mL separatory funnel, to which 70 mL diethyl ether was added. The organic phase was preserved and the aqueous phase was extracted twice with diethyl ether (Et_2O, 70 mL). The organic phases were combined and washed with a saturated sodium chloride solution (NaCl, 70 mL), then dried over magnesium sulfate, filtered through cotton and concentrated under reduced pressure in a flask. The solid obtained after this step was recrystallized three times in cold methanol. The di-octyl muconate crystals are placed in a Schlenk flask with a magnetic stirring bar and the product was dried by azeotropic distillation in toluene (2 mL) for 30 min two times. A final azeotropic distillation with toluene (2 mL) was carried out overnight. The product was kept under argon. Yield: > 95%.

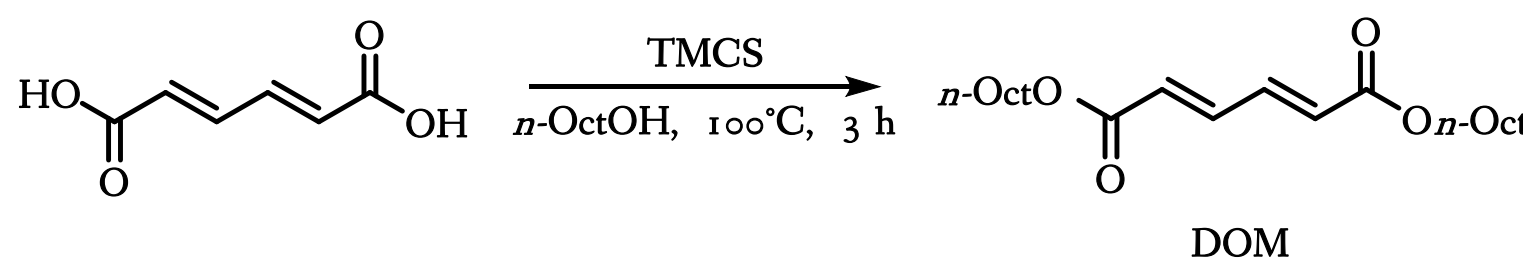

Group transfer copolymerization (GTcP) of trans,trans-dialkyl muconates:

General polymerization procedure. All (co)polymerization reactions were performed at room temperature (290-300 K) and in an argon-filled glovebox.

Synthesis of P(E,E-DEM)$_{20}$-co-P(E,E-DOM)$_{80}$ in toluene with P$_4$-*t*-Bu catalyst. In a typical procedure ([DEM]$_0$+[DOM]$_0$/[ETSB]$_0$/[P$_4$-*t*-Bu]$_0$= 20+80/1/0.01), 200 μL of a solution of diethyl muconate dissolved in toluene (1 mol.L^{-1} in toluene, 0.2 mmol), 800 μL of a solution of dioctyl muconate (1 mol.L^{-1} in toluene, 0.8 mmol) and 2 mL of dried toluene were added in a previously flame-dried 10 mL Schlenk flask containing a magnetic stirring bar. The mixture was stirred for 30 s and afterwards 100 μL of an ETSB solution (100 mmol.L^{-1} in toluene, 10 μmol) and 100 μL of a P$_4$-*t*-Bu catalyst solution (10 mmol.L^{-1} in toluene, 0.1 μmol) were added. The solution turned slightly yellow-orange when the catalyst solution was added. After stirring for 1 min, the reaction was quenched by adding few drops of benzoic acid dissolved in THF and the solution turned colorless. A sample was analyzed by ^{1}H NMR to control the complete conversion of the monomer then solvent and volatiles were removed under vacuum and the remaining polymer was dissolved in a small amount of THF. The copolymer was precipitated in cold methanol and recovered as a solid. The product was analyzed by ^{1}H and ^{13}C NMR, SEC and MALDI-ToF MS. Yield: > 99%, $M_{n,theo.}$ = 33 200 g.mol^{-1}. $M_{n,SEC}$ = 54 000 g.mol^{-1}, and M_w/M_n = 1.54.

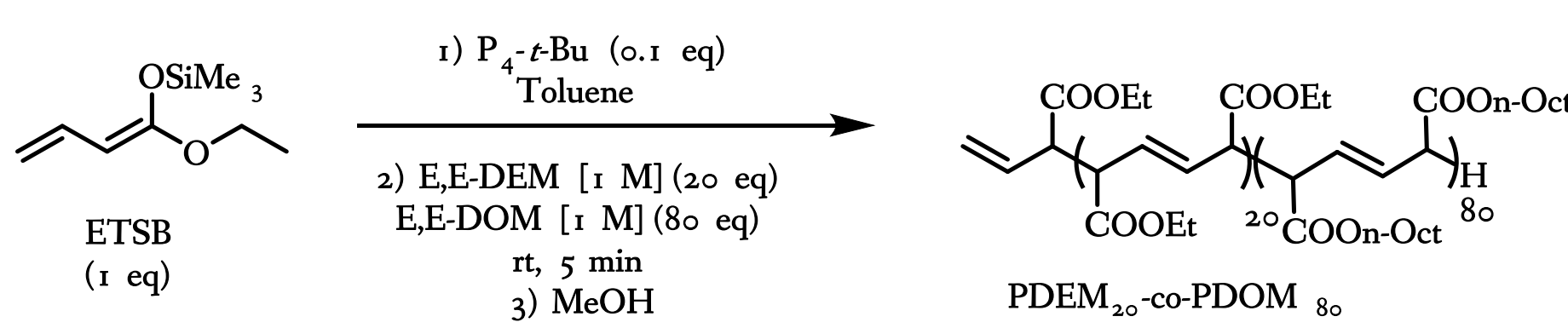

Synthesis of P(E,E-DEM)$_{20}$-co-P(E,E-DOM)$_{80}$ in THF with P$_2$-*t*-Bu catalyst. In a typical procedure ([DEM]$_0$+[DOM]$_0$/[ETSB]$_0$/[P$_2$-*t*-Bu]$_0$= 20+80/1/0.1), 200 μL of a solution of diethyl muconate dissolved in

THF (1 mol.L^{-1} in THF, 0.2 mmol), 800 μL of a solution of dioctyl muconate (1 mol.L^{-1} in THF, 0.8 mmol) and 2 mL of dried THF were added in a previously flame-dried 10 mL Schlenk flask containing a magnetic stirring bar. The mixture was stirred for 30s and afterwards 100 μL of an ETSB solution (100 mmol.L^{-1} in THF, 10 μmol) and 100 μL of a P_2-*t*-Bu catalyst solution (10 mmol.L^{-1} in THF, 1 μmol) were added. The solution turned slightly yellow-orange when the catalyst solution was added. After stirring for 5 min, the reaction was quenched by adding few drops of benzoic acid dissolved in THF and the solution turned colorless. A sample was analyzed by ^{1}H NMR to control the complete conversion of the monomer then solvent and volatiles were removed under vacuum and the remaining polymer was dissolved in a small amount of THF. The copolymer was precipitated in cold methanol and recovered as a solid. The product was analyzed by ^{1}H and ^{13}C NMR, SEC and MALDI-ToF MS. Yield: > 99%, $M_{n,theo.}$ = 33 200 g.mol^{-1}. $M_{n,SEC}$ = 24 000 g.mol^{-1}, and M_w/M_n = 1.15.

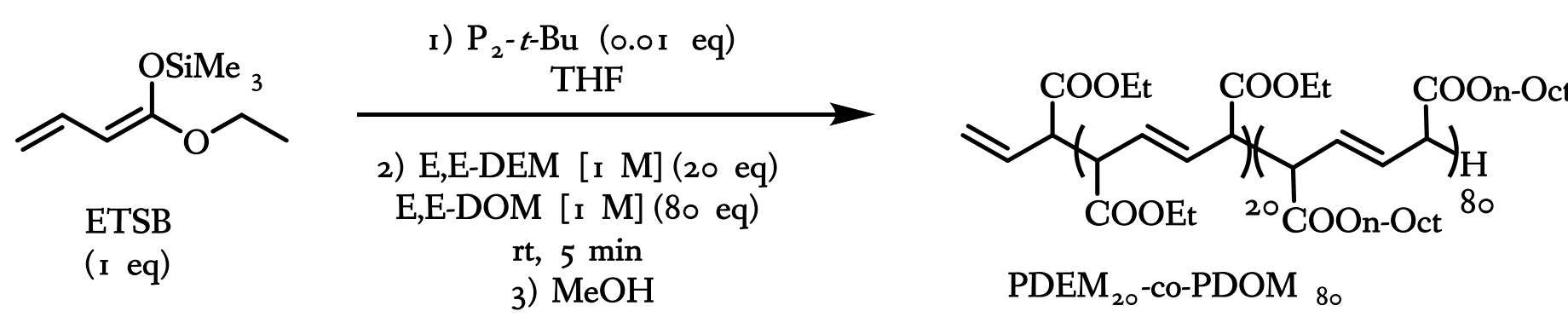

Synthesis of P(E,E-DEM)$_{60}$-co-P(E,E-DOM)$_{40}$ in isododecane with P_4-*t*-Bu catalyst. In a typical procedure ($[DEM]_0+[DOM]_0/[ETSB]_0/[P_4\text{-}t\text{-}Bu]_0$= 20+80/1/0.01), 118.8 mg of diethyl muconate (0.6 mmol), 146.4 mg of dioctyl muconate (0.4 mmol) and 5 mL of dried isododecane were added in a previously flame-dried 10 mL Schlenk flask containing a magnetic stirring bar. The mixture was stirred until complete dissolution and afterwards 100 μL of an ETSB solution (100 mmol.L^{-1} in isododecane, 10 μmol) and 100 μL of a P_4-*t*-Bu catalyst solution (1 mmol.L^{-1} in isododecane, 0.1 μmol) were added. The solution turned slightly yellow-orange and turbid when the catalyst solution was added. After stirring for 5 min, the reaction was quenched by adding few drops of benzoic acid dissolved in THF and the solution turned slightly blue and turbid. A sample was analyzed by ^{1}H NMR to control the complete conversion of the monomer. The product was analyzed by ^{1}H and ^{13}C NMR, SEC and MALDI-ToF MS. Yield: > 99%, $M_{n,theo.}$ = 26 500 g.mol^{-1}. $M_{n,SEC}$ = 29 300 g.mol^{-1}, and M_w/M_n = 1.96.

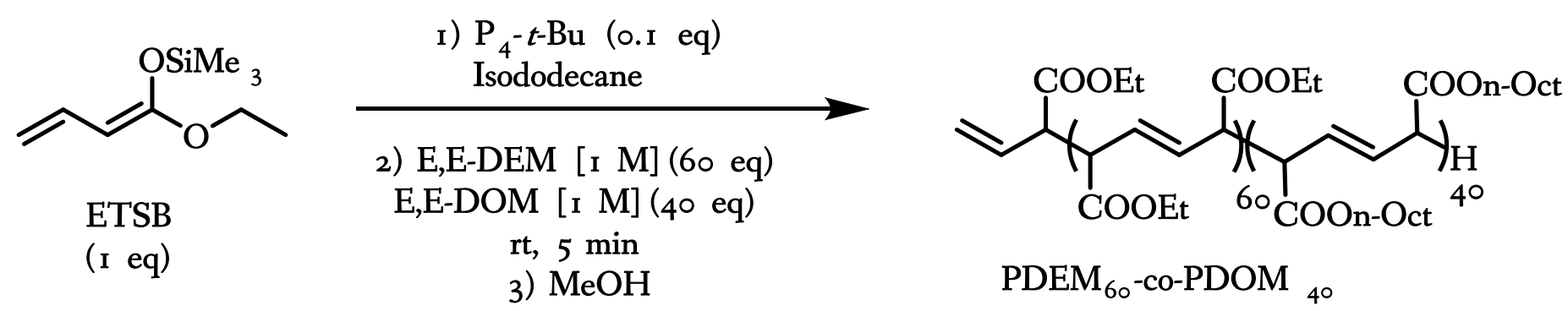

Depolymerization of PDEM$_{50}$-co-PDOM$_{50}$. In a typical procedure, 1 g of dry PDEM$_{50}$-co-PDOM$_{50}$ was solubilized in 50 mL of diphenyl ether (DPE). The solution was bubbled with argon for 10 minutes and was at 260 °C for 20 min under reflux conditions. The solution progressively turned slight yellow. The reaction was cooled in an ice bath to stop the reaction. An aliquot was taken and analyzed by ^{1}H NMR and by SEC. The resulting mixture was then purified by a two-step silica column. Firstly, an eluent consisting of 3:2 hexane:dichloromethane was used to remove residual DPE. Secondly, a 2:3 hexane:chloroform elution system was employed to separate each monomer mixture. A mixture containing E,E-DEM and E,Z-DEM could thus be isolated from a mixture containing E,E-DOM and E,Z-DOM (75% yield in total), as evidenced by characterization by ^{1}H NMR.

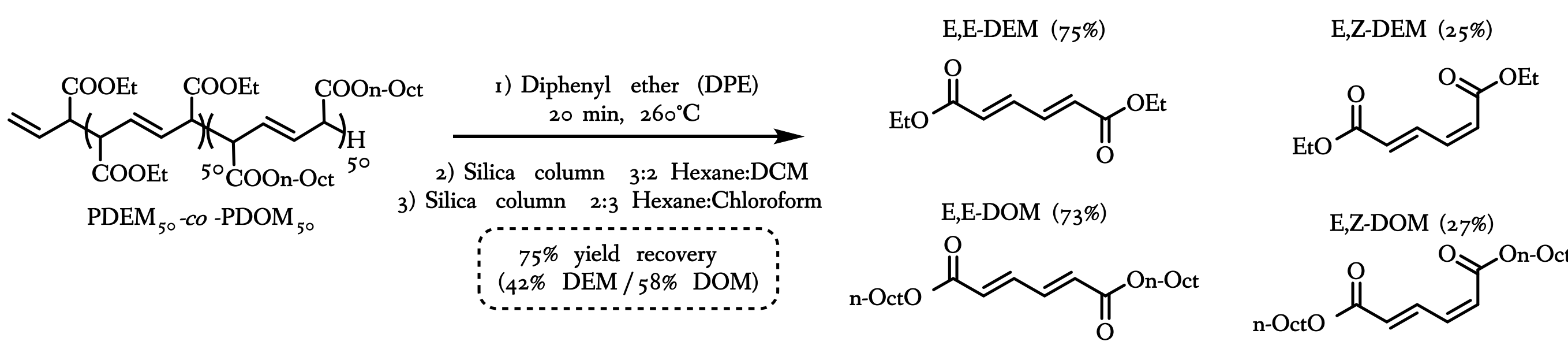

O-GTcP dissociative mechanism:

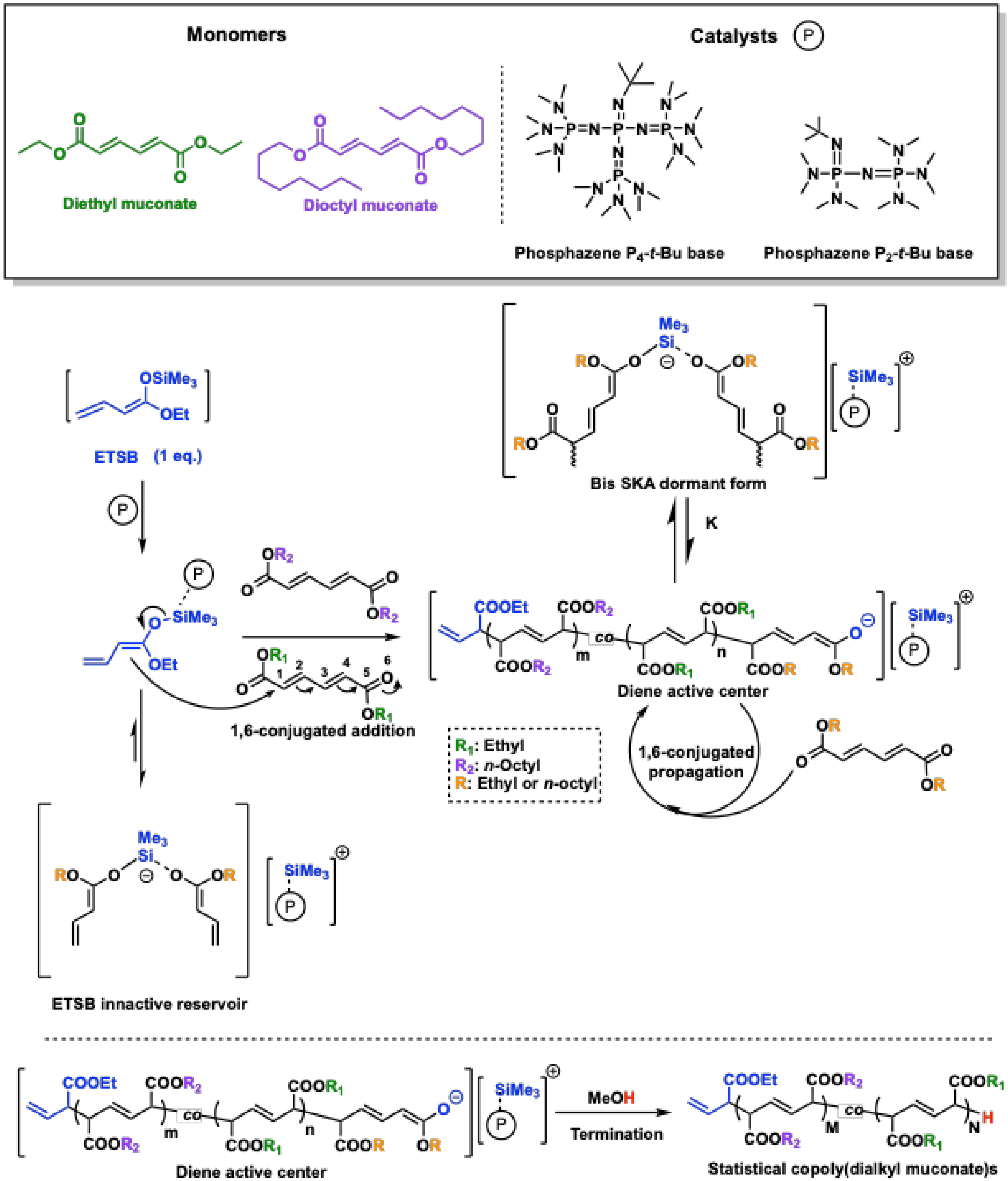

Scheme S1. Dissociative mechanism of phosphazene-base catalyzed group transfer copolymerization of t,t-diethyl muconate and t,t-dioctyl muconate, initiated by 1-ethoxy-1-(trimethylsiloxy)-1,3-butadiene (ETSB).

Regioselectivity and geometrical selectivity of muconate O-GTP:

Regioselectivity. In the O-GTcP of dialkyl muconates, the propagating species is a stabilized diene-enolate of the SKA type. Its conjugate nucleophilic attack onto the muconate monomer occurs at the terminal carbon of the conjugated diene system, resulting in a 1,6-conjugate addition and generating the corresponding 1,4-addition repeat unit in the polymer backbone. We therefore consider " 1,6-conjugate addition polymerization" to be a more mechanistically explicit description of this process.

Importantly, our spectroscopic data provide experimental evidence for the regioselectivity of the reaction. Alternative 3,6-conjugate addition would generate a different unsaturated structural motif, for which characteristic resonances have been reported at approximately 6.3-6.9 ppm in the ^{1}H NMR spectra and around 125 and 165 ppm in the ^{13}C NMR spectra (see for instance ref. 17). These diagnostic signals are absent from the NMR spectra of our copolymuconates prepared by O-GTcP (**Figures S49–S56**). We therefore find no spectroscopic evidence for the formation of 3,6-addition units, supporting the essentially exclusive formation of the 1,4-addition structure.

This regioselectivity is also consistent with the proposed reaction mechanism. The 1,6-conjugate addition generates a highly stabilized diene-enolate propagating species in which the remaining double bonds are conjugated, whereas the alternative 3,6-addition would disrupt this favorable conjugation and generate a less stabilized enolate-type active center. The electronic and steric environment of the conjugated muconate system therefore favors the observed 1,6-conjugate addition mechanism.

From a mechanistic perspective, we propose that 3,6-conjugated addition is disfavored because the diene-enolate active center formed after monomer addition is more stabilized by conjugation between the two double bonds than the simple enolate that would result from a 3,6-addition pathway (**Scheme S2**).

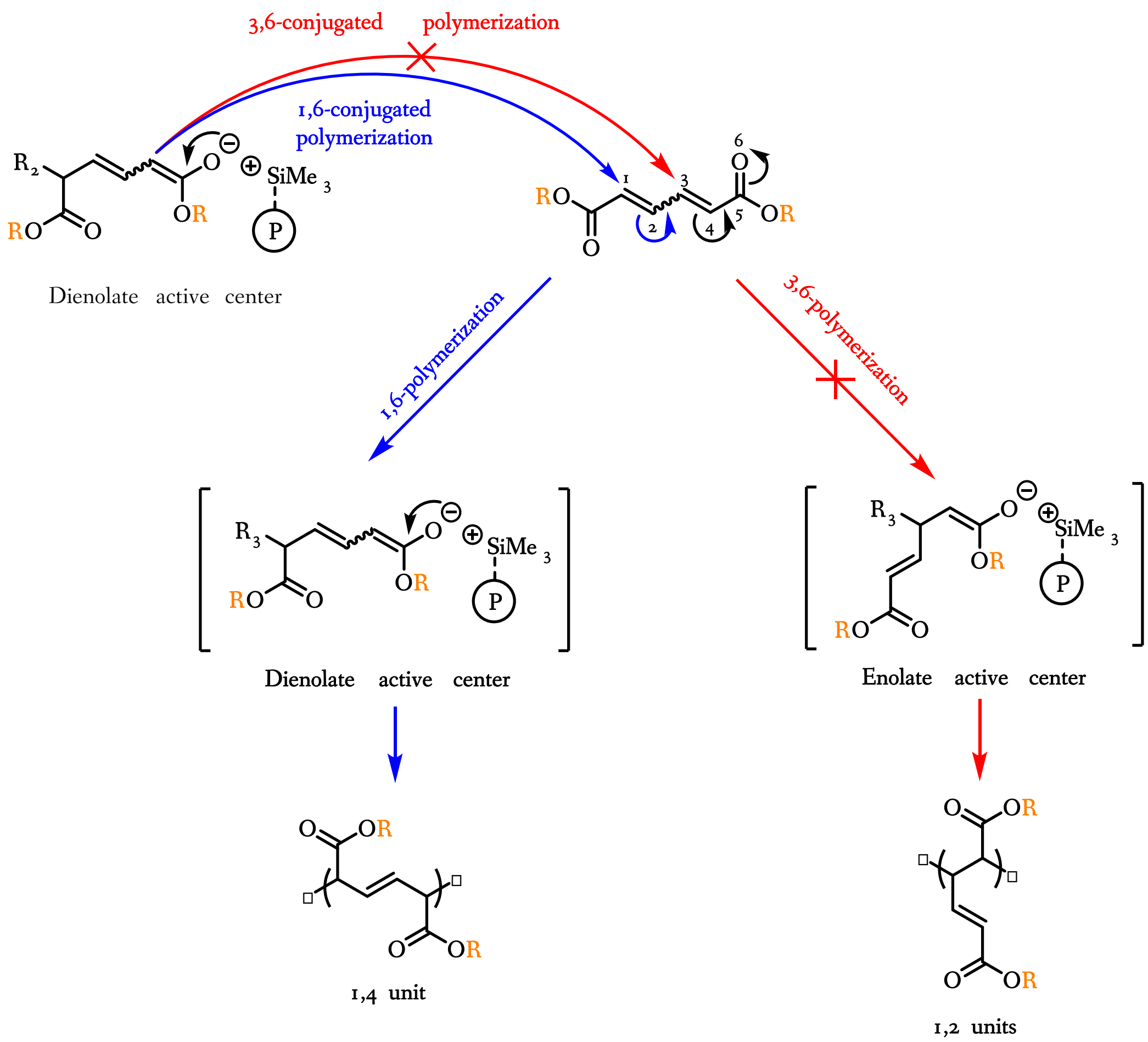

Scheme S2. 1,6- and 3,6-conjugated addition reactions of the propagating dienolate onto a dialkyl muconate monomer. The structural motifs resulting from the 3,6-addition have not been identified experimentally. The absence of this reaction is attributed to the lower electrophilicity of carbon atoms 2 and 3, relatively to carbon atoms 1 and 4.

Geometrical selectivity. The copolymerization is not exclusively *trans*-selective. Starting from the predominantly *trans,trans*-muconate monomer, rotation around the central C3-C4 single bond allows two relevant conformations of the conjugated diene system. These conformers lead to different geometrical configurations of the newly formed polymer units (**Scheme S3**; see ref. 15). The more accessible conformation preferentially leads to the *trans*-1,4 configuration, while the alternative conformation gives the *cis*-1,4 configuration. Experimentally, we observe a predominantly *trans*-1,4 product, with approximately 80% *trans*-1,4 and 20% *cis*-1,4-addition units.

In summary, the O-GTcP of dialkyl muconates is characterized by high regioselectivity for 1,4-addition units arising from 1,6-conjugate addition, together with a pronounced but not complete geometrical selectivity toward the *trans* configuration.

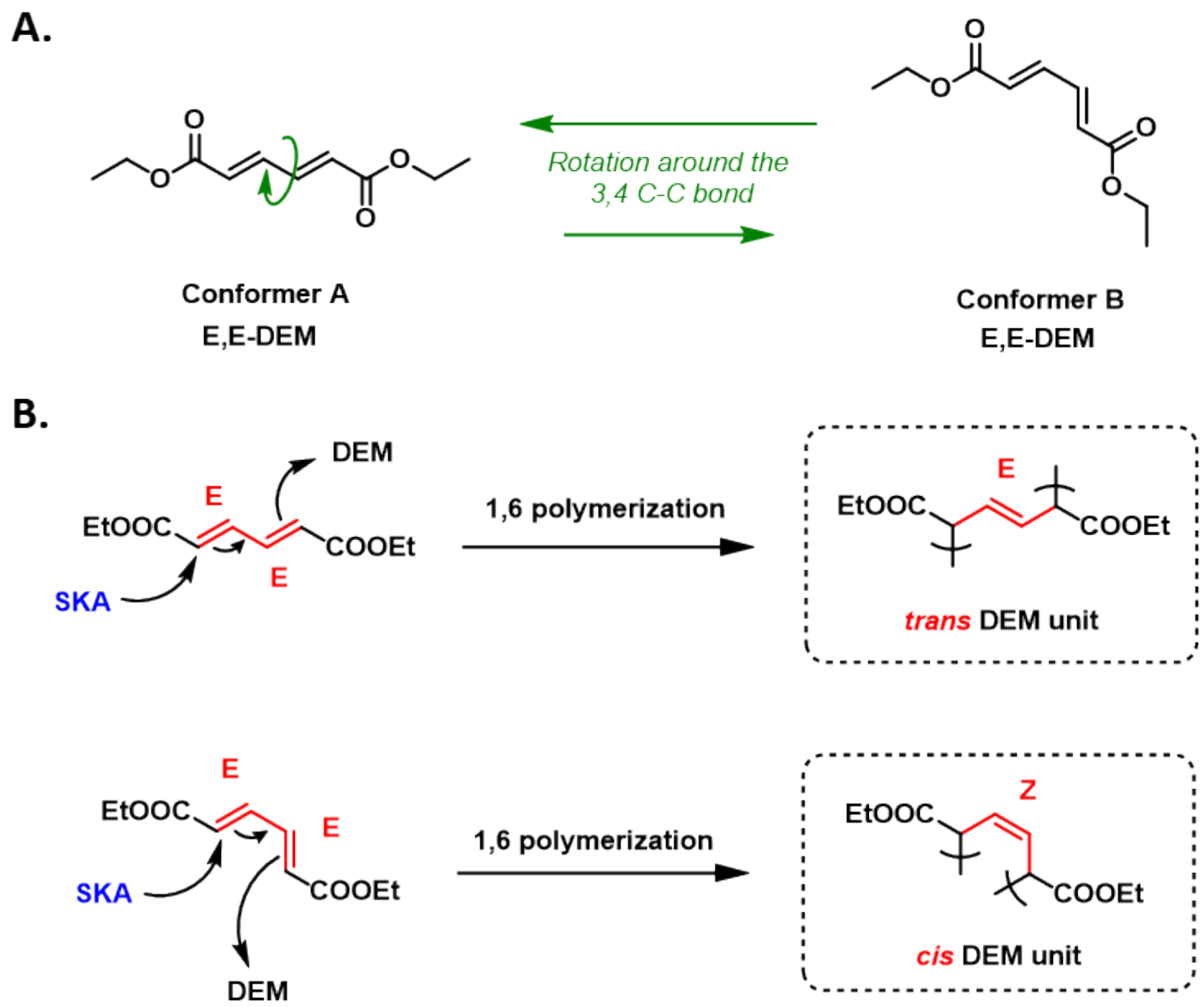

Scheme S3. A. Representation of E,E-DEM showing two different conformations (conformers A and B) depending of the rotation around the simple 3,4 C-C bond **B.** Simplified mechanism of the 1,6-conjugate nucleophilic attack of the propagating SKA-type diene-enolate on E,E-DEM of two distinct conformations, leading to two different conformations of the resulting DEM units, *i.e., trans* and *cis* units.

O-GTcP monitoring:

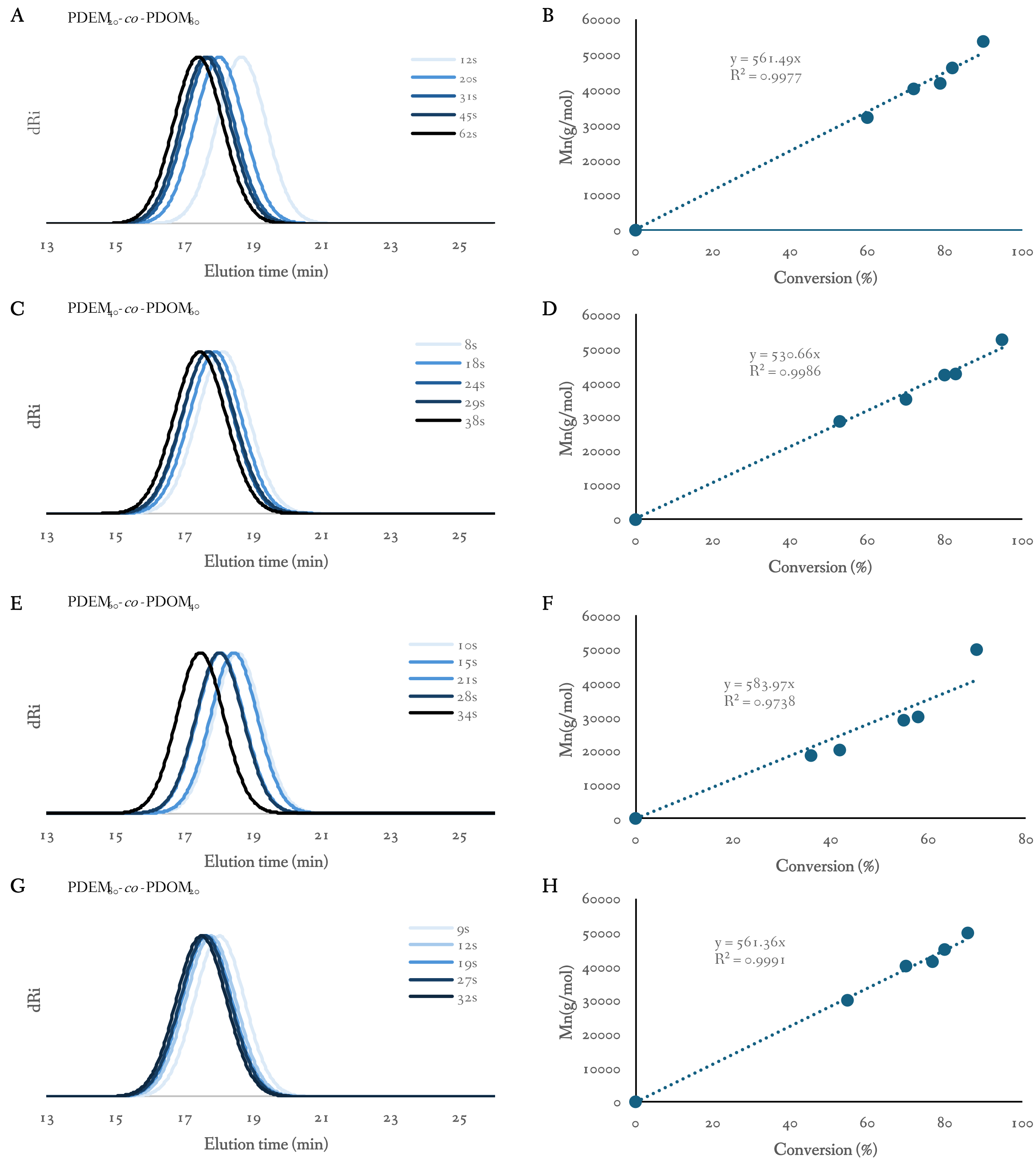

Figure S1. Molecular weight analysis done by SEC for entries using toluene as solvent and P_4-*t*-Bu as catalyst. **A.** Evolution of molecular weight distribution with the progress of the polymerization of PDEM$_{20}$-*co*-PDOM$_{80}$. **B.** Molecular weight as a function of global conversion to examine the presence of transfer reactions. **C.** Evolution of molecular weight distribution with the progress of the polymerization of PDEM$_{50}$-*co*-PDOM$_{50}$. **D.** Molecular

weight as a function of global conversion to examine the presence of transfer reactions. **E.** Evolution of molecular weight distribution with the progress of the polymerization of $PDEM_{60}$-*co*-$PDOM_{40}$. **F.** Molecular weight as a function of global conversion to examine the presence of transfer reactions. **G.** Evolution of molecular weight distribution with the progress of the polymerization of $PDEM_{80}$-*co*-$PDOM_{20}$. **H.** Molecular weight as a function of global conversion to examine the presence of transfer reactions.

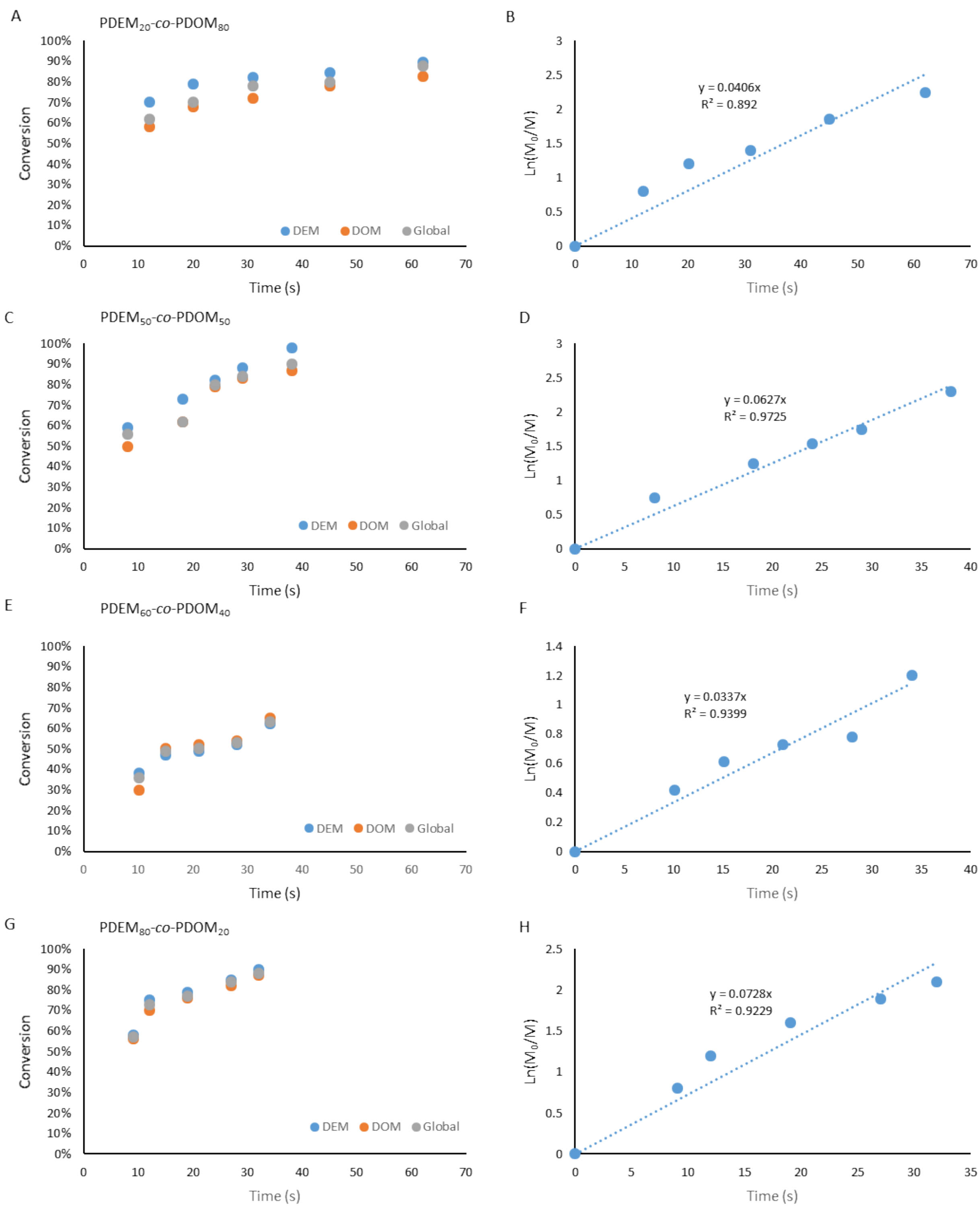

Figure S2. Monitoring of copolymerization using toluene as solvent and P_4-*t*-Bu as catalyst. **A.** Conversion of DEM and DOM during the synthesis of $PDEM_{20}$-*co*-$PDOM_{80}$. **B.** Ln($[M]_o$/[M]) as a function of time, where $[M]_o$ is the initial concentration of DEM and DOM together, and M is the concentration of DEM and DOM at the time of

the sampling. **C.** Conversion of DEM and DOM during the synthesis of $PDEM_{50}$-*co*-$PDOM_{50}$. **D.** Ln($[M]_0/[M]$) as a function of time. **E.** Conversion of DEM and DOM during the synthesis of $PDEM_{60}$-*co*-$PDOM_{40}$. **F.** Ln($[M]_0/[M]$) as a function of time. **G.** Conversion of DEM and DOM during the synthesis of $PDEM_{80}$-*co*-$PDOM_{20}$. **H.** Ln($[M]_0/[M]$) as a function of time.

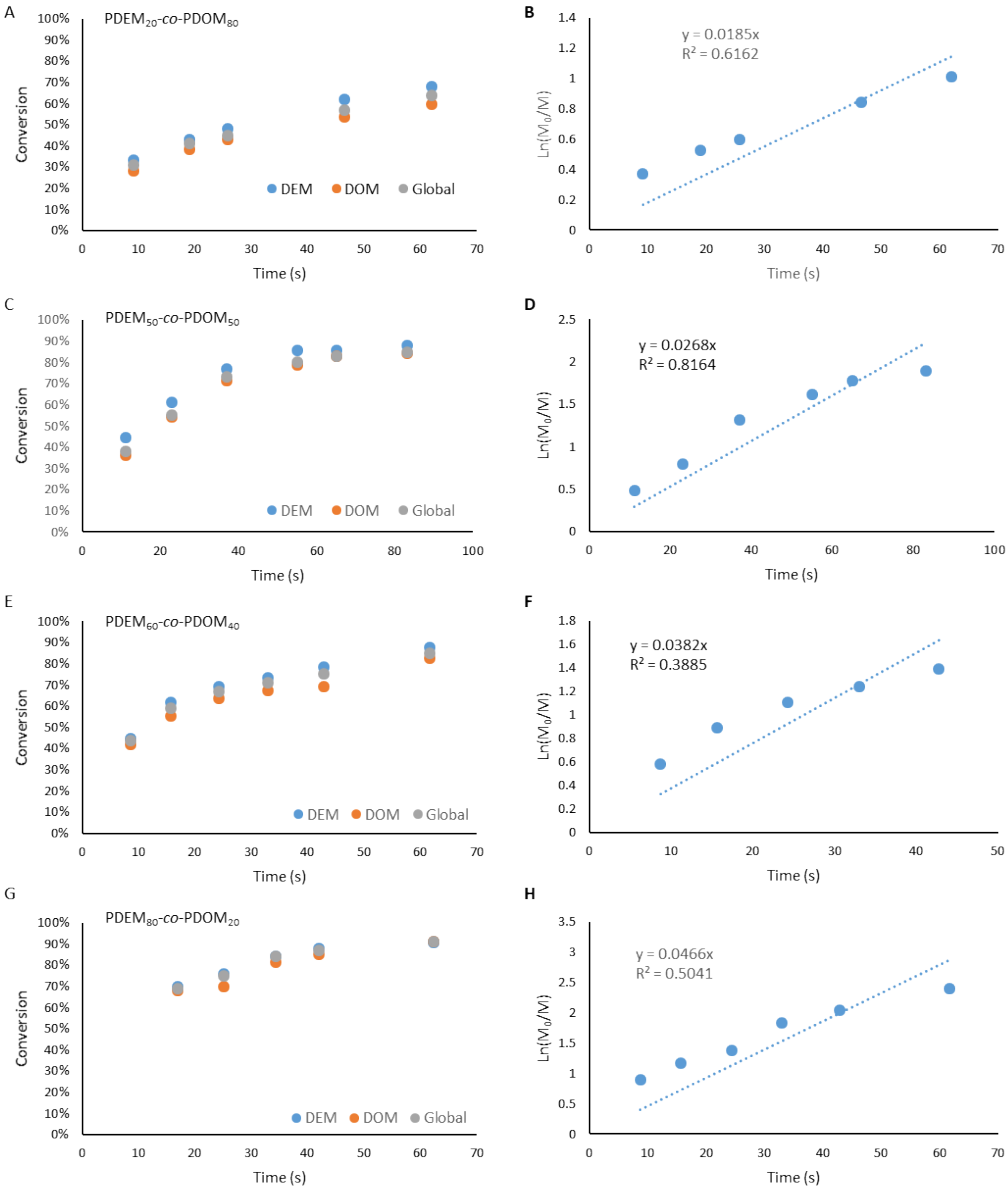

Figure S3. Monitoring of copolymerization using THF as solvent and P_4-*t*-Bu as catalyst. **A.** Conversion of DEM and DOM during the synthesis of $PDEM_{20}$-*co*-$PDOM_{80}$. **B.** Ln($[M]_0/[M]$) as a function of time, where $[M]_0$ is the initial concentration of DEM and DOM together, and M is the concentration of DEM and DOM at the time of the sampling. **C.** Conversion of DEM and DOM during the synthesis of $PDEM_{50}$-*co*-$PDOM_{50}$. **D.** Ln($[M]_0/[M]$) as a function of time. **E.** Conversion of DEM and DOM during the synthesis of $PDEM_{60}$-*co*-$PDOM_{40}$. **F.**

Ln([M]$_0$/[M]) as a function of time. **G.** Conversion of DEM and DOM during the synthesis of PDEM$_{80}$-*co*-PDOM$_{20}$. **H.** Ln([M]$_0$/[M]) as a function of time.

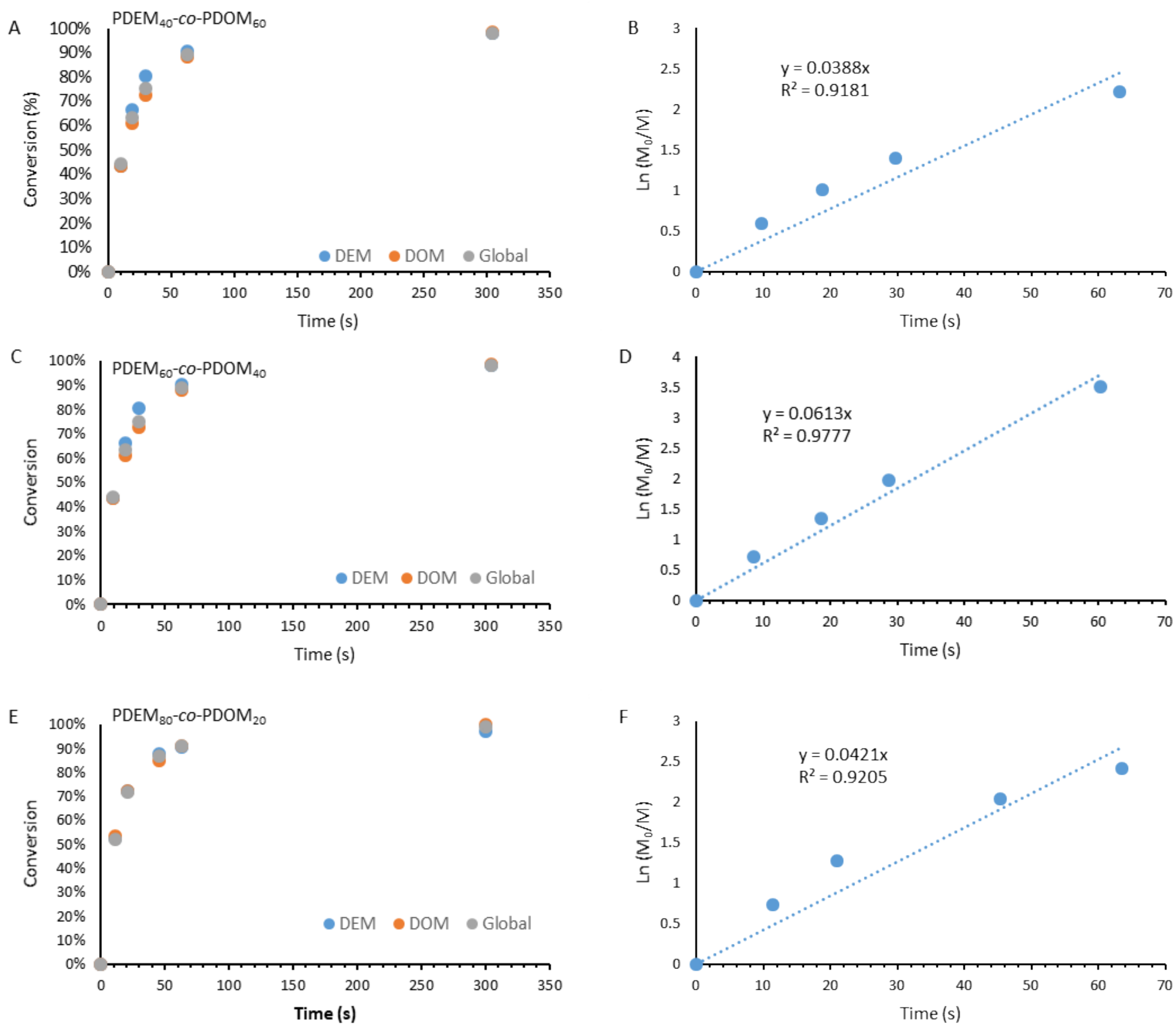

Figure S4. Monitoring of copolymerization using THF as solvent and P_2-*t*-Bu as catalyst. **A.** Conversion of DEM and DOM during the synthesis of PDEM$_{40}$-*co*-PDOM$_{60}$. **B.** Ln([M]$_0$/[M]) as a function of time, where [M]$_0$ is the initial concentration of DEM and DOM together, and M is the concentration of DEM and DOM at the time of the sampling. **C.** Conversion of DEM and DOM during the synthesis of PDEM$_{60}$-*co*-PDOM$_{40}$. **D.** Ln([M]$_0$/[M]) as a function of time. **E.** Conversion of DEM and DOM during the synthesis of PDEM$_{20}$-*co*-PDOM$_{80}$. **F.** Ln([M]$_0$/[M]) as a function of time.

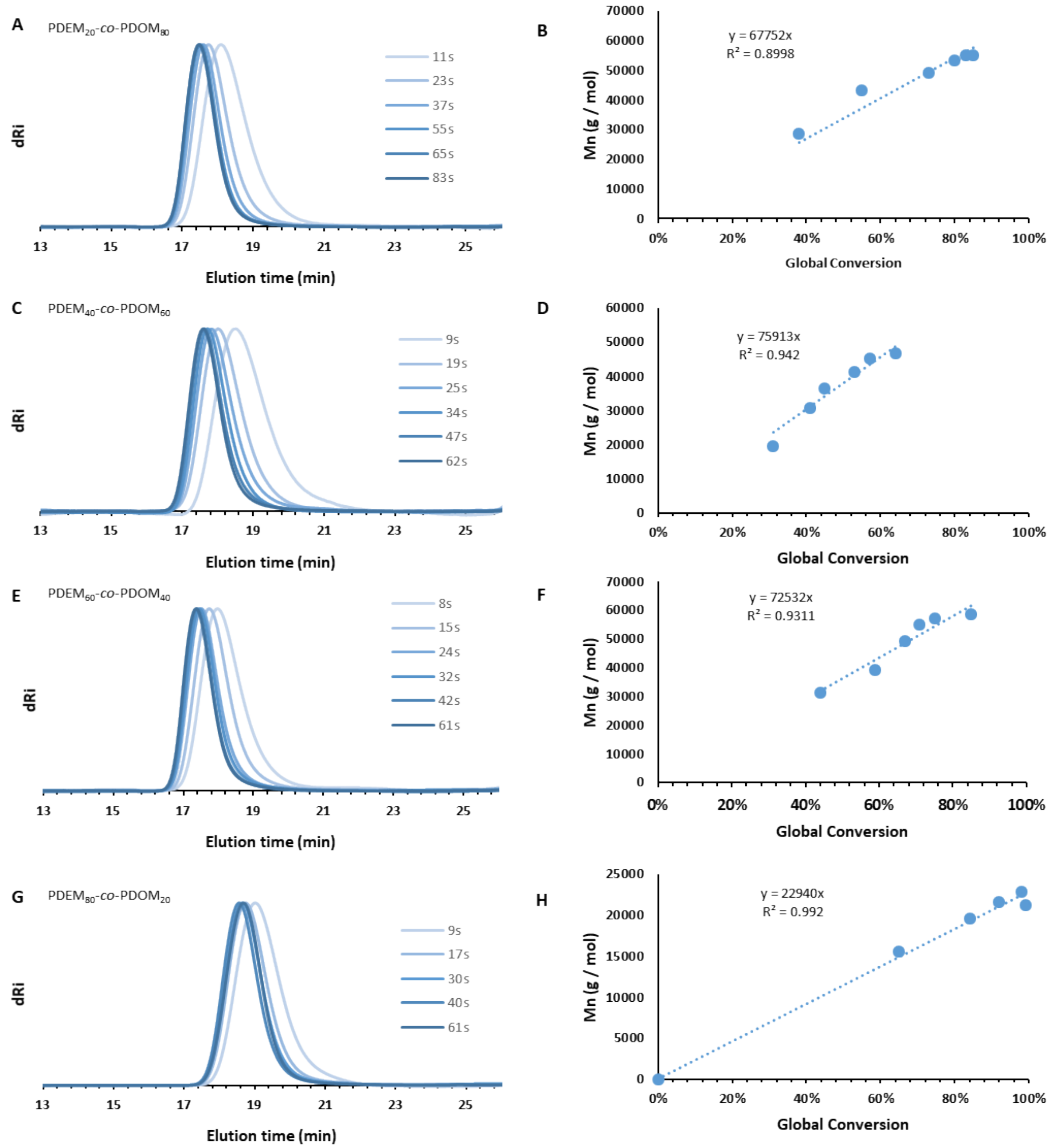

Figure S5. Molecular Weight analysis done by SEC for entries using THF as solvent and P_4-*t*-Bu as catalyst. **A.** Evolution of molecular weight distribution with the progress of the polymerization of $PDEM_{20}$-*co*-$PDOM_{80}$. **B.** Molecular weight as a function of global conversion to examine the presence of transfer reactions. **C.** Evolution of molecular weight distribution with the progress of the polymerization of $PDEM_{50}$-*co*-$PDOM_{50}$. **D.** Molecular weight as a function of global conversion to examine the presence of transfer reactions. **E.** Evolution of molecular weight distribution with the progress of the polymerization of $PDEM_{60}$-*co*-$PDOM_{40}$. **F.** Molecular weight as a function of global conversion to examine the presence of transfer reactions. **G.** Evolution of molecular weight

distribution with the progress of the polymerization of $PDEM_{80}$-*co*-$PDOM_{20}$. **H.** Molecular weight as a function of global conversion to examine the presence of transfer reactions.

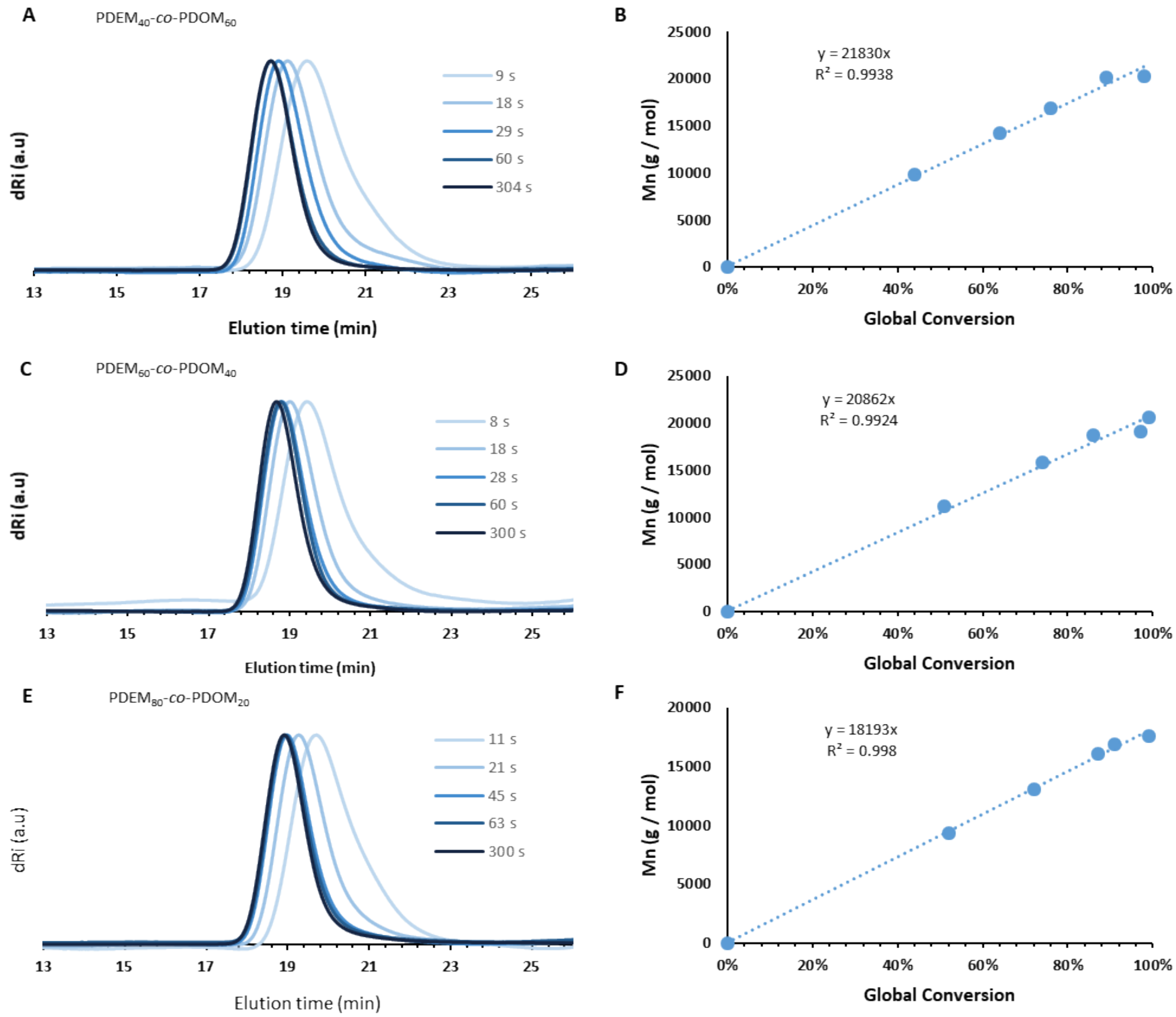

Figure S6. Molecular Weight analysis done by SEC for entries using THF as solvent and P_2-*t*-Bu as catalyst. **A.** Evolution of molecular weight distribution with the progress of the polymerization of PDEM$_{40}$-*co*-PDOM$_{60}$. **B.** Molecular weight as a function of global conversion to examine the presence of transfer reactions. **C.** Evolution of molecular weight distribution with the progress of the polymerization of PDEM$_{60}$-*co*-PDOM$_{40}$. **D.** Molecular weight as a function of global conversion to examine the presence of transfer reactions. **E.** Evolution of molecular weight distribution with the progress of the polymerization of PDEM$_{80}$-*co*-PDOM$_{20}$. **F.** Molecular weight as a function of global conversion to examine the presence of transfer reactions.

Determination of reactivity ratios during O-GTcP:

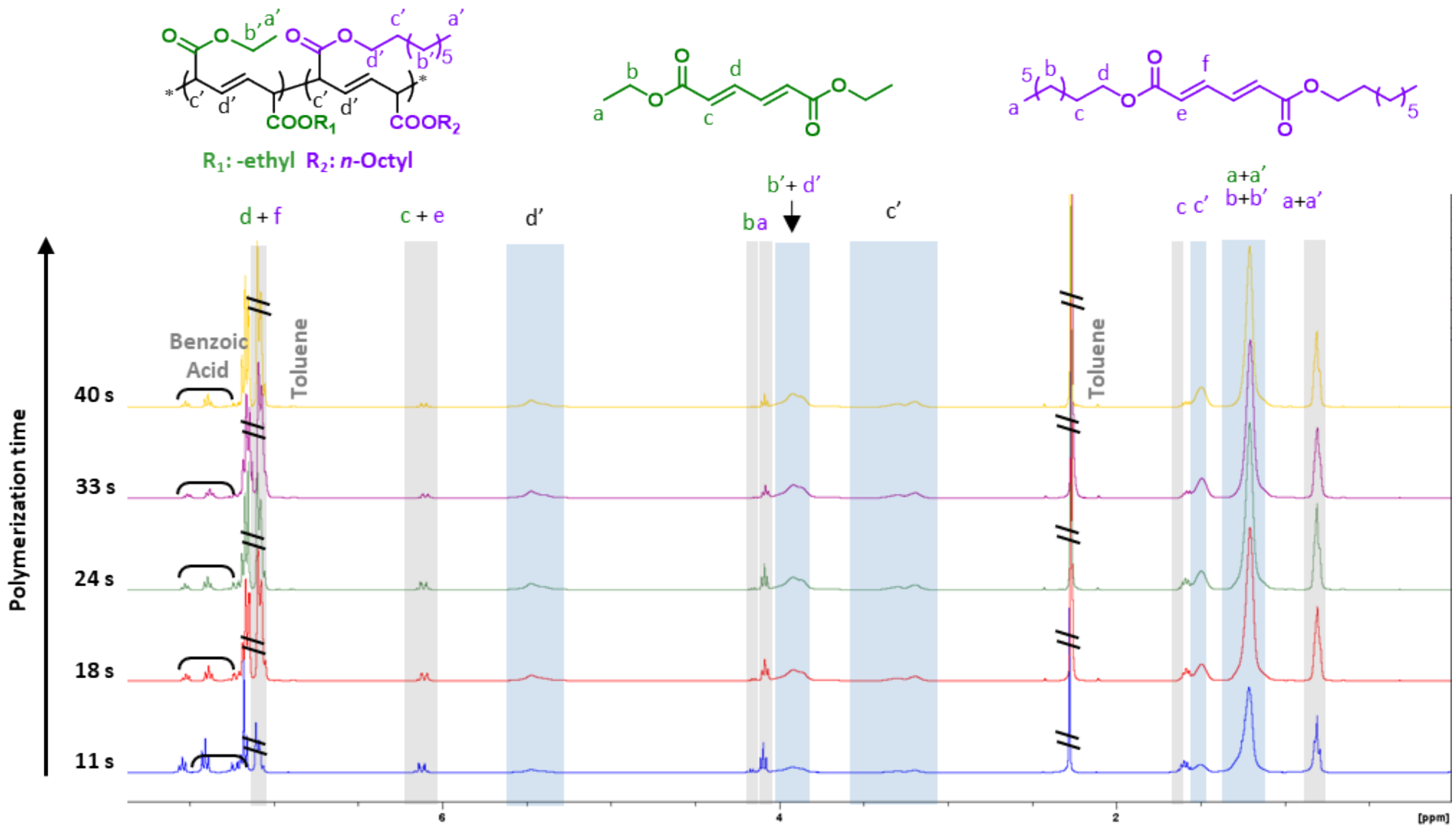

Figure S7. Monitoring study by ^{1}H NMR of one-pot copolymerization of DEM (green) and DOM (purple) in toluene using P_4-*t*-Bu as catalyst (Table 1, entry 1), showing the changes in the characteristic peaks of the monomer after the start of the polymerization: 11 s (blue spectrum), 18 s (red spectrum), 24 s (green spectrum), 33 s (purple spectrum) and 40 s (yellow spectrum).

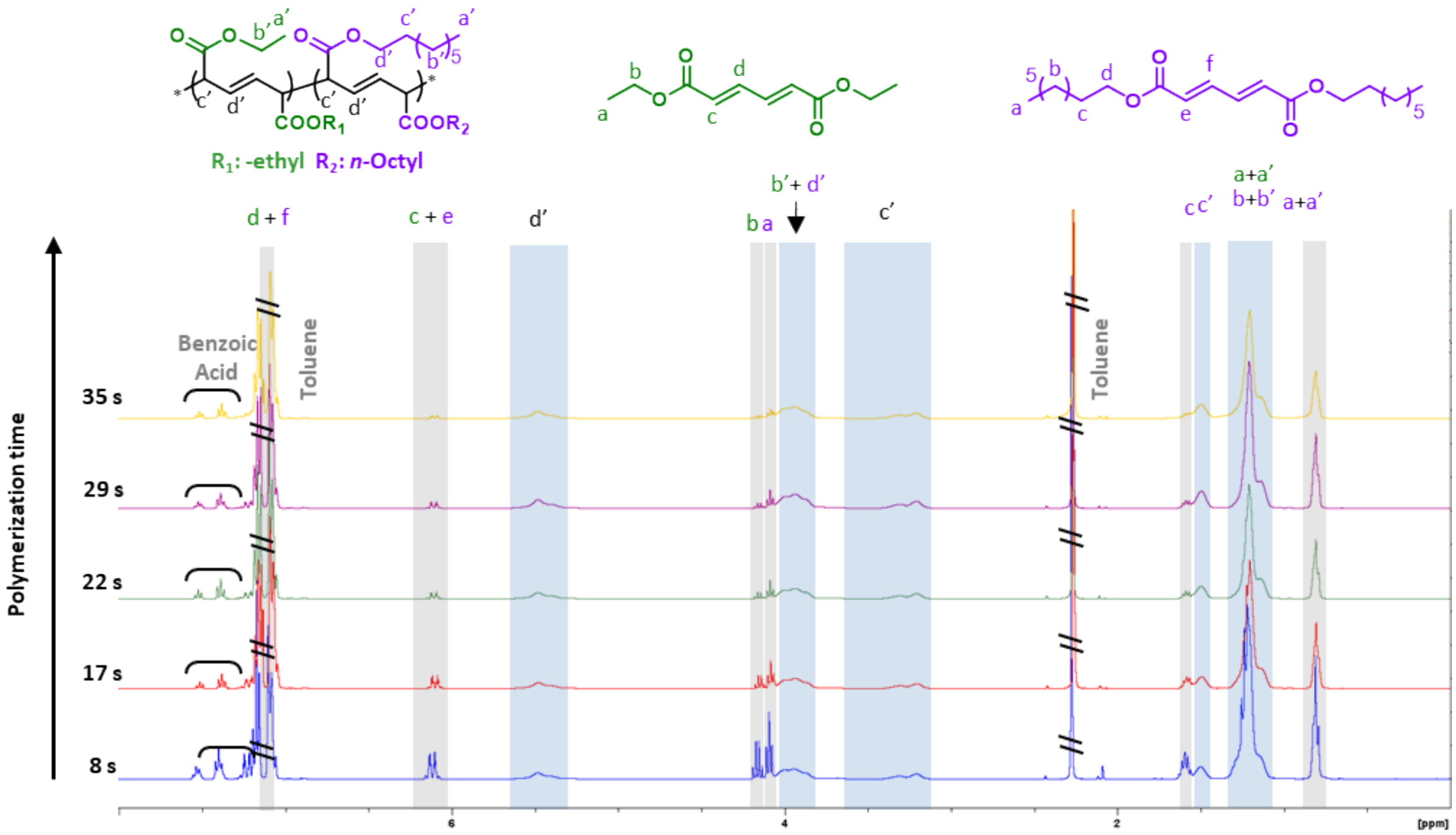

Figure S8. Monitoring study by ¹H NMR of one-pot copolymerization of DEM (green) and DOM (purple) in toluene using P_4-*t*-Bu as catalyst (Table 1, entry 2), showing the changes in the characteristic peaks of the monomer after the start of the polymerization: 8 s (blue spectrum), 17 s (red spectrum), 22 s (green spectrum), 29 s (purple spectrum) and 35 s (yellow spectrum).

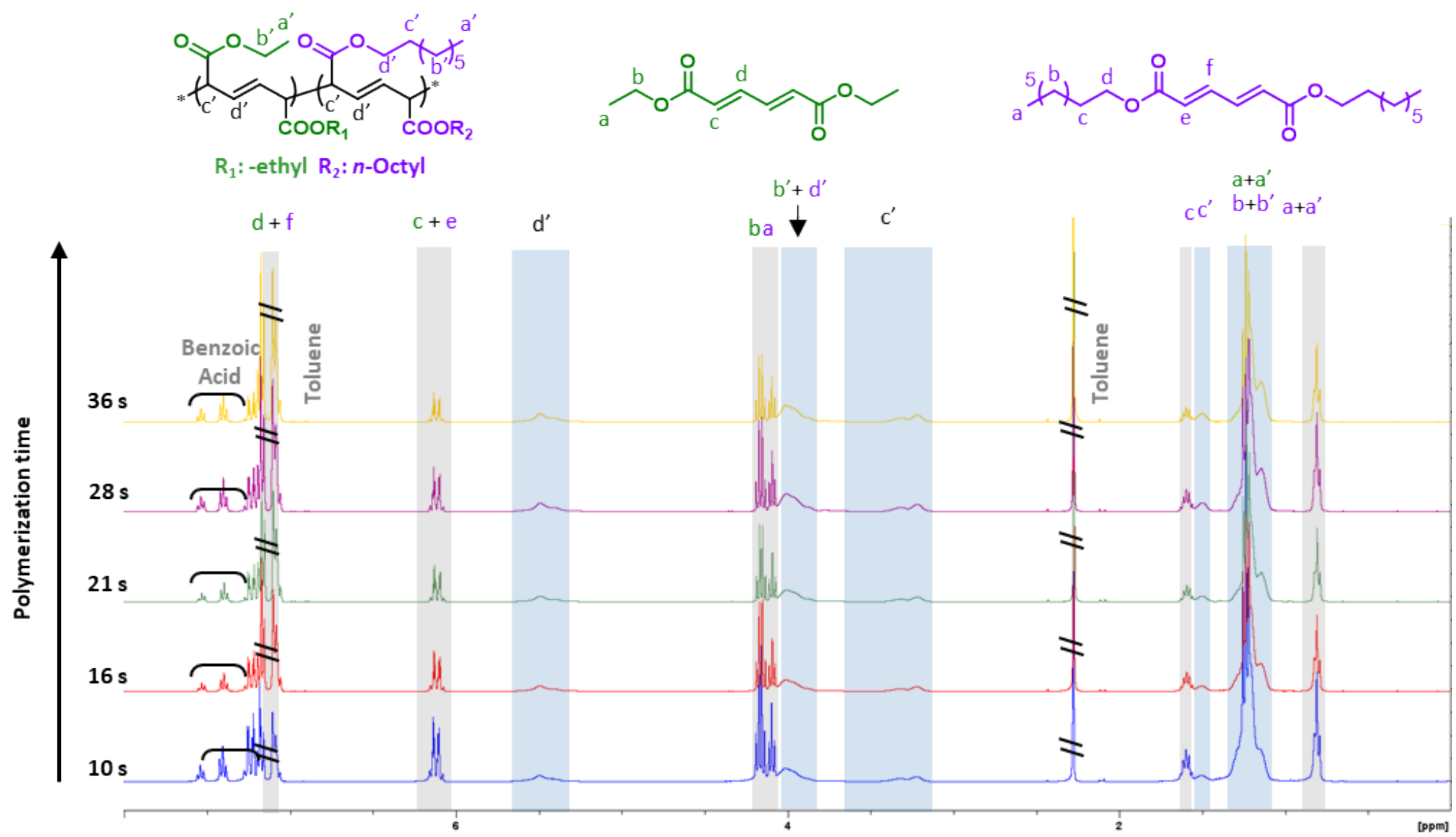

Figure S9. Monitoring study by ^{1}H NMR of one-pot copolymerization of DEM (green) and DOM (purple) in toluene using P_4-*t*-Bu as catalyst (Table 1, entry 3), showing the changes in the characteristic peaks of the monomer after the start of the polymerization: 10 s (blue spectrum), 16 s (red spectrum), 21 s (green spectrum), 28 s (purple spectrum) and 36 s (yellow spectrum).

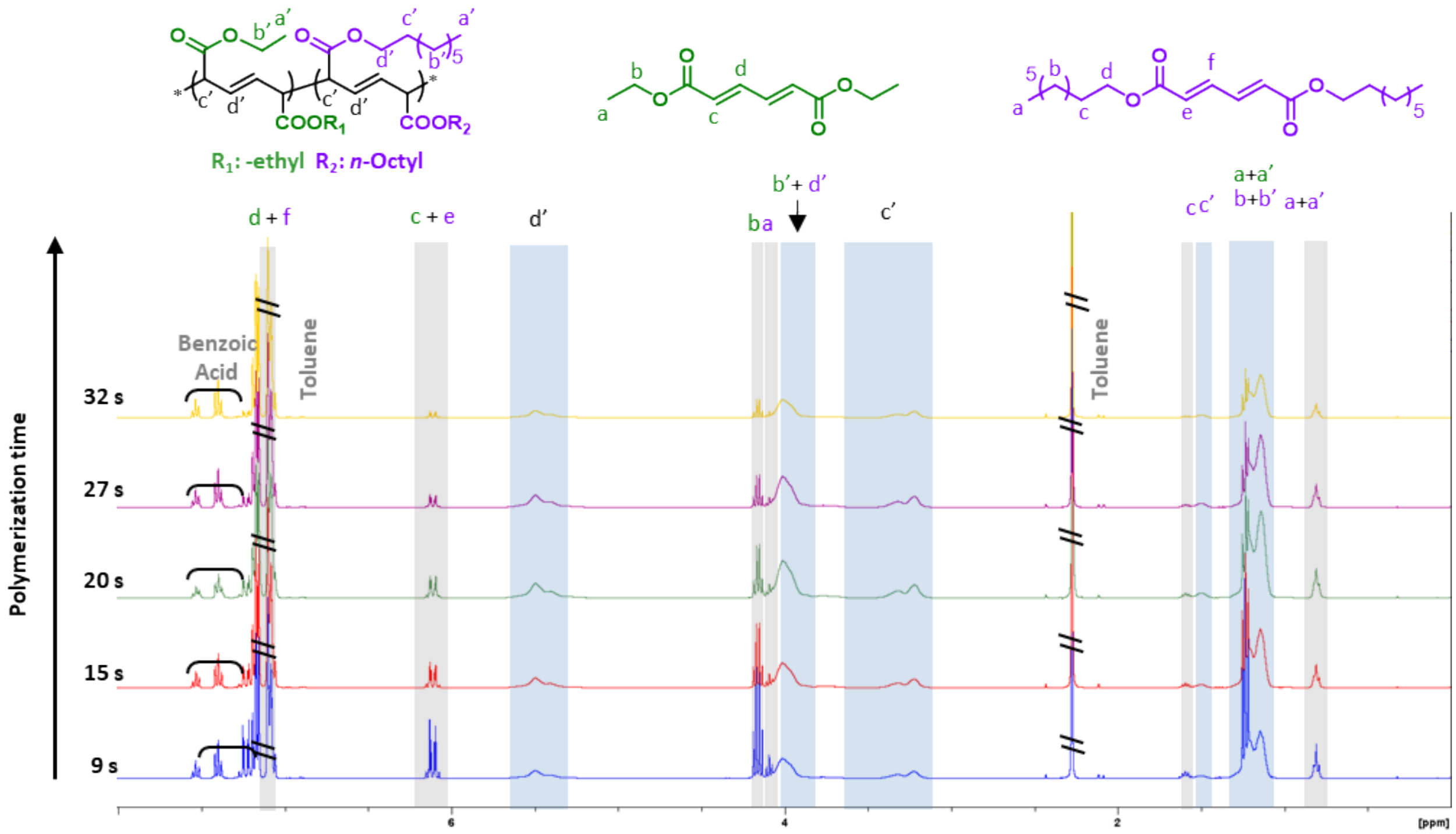

Figure S10. Monitoring study by ^{1}H NMR of one-pot copolymerization of DEM (green) and DOM (purple) in toluene using P_4-*t*-Bu as catalyst (Table 1, entry 4), showing the changes in the characteristic peaks of the monomer after the start of the polymerization: 9 s (blue spectrum), 15 s (red spectrum), 20 s (green spectrum), 27 s (purple spectrum) and 32 s (yellow spectrum).

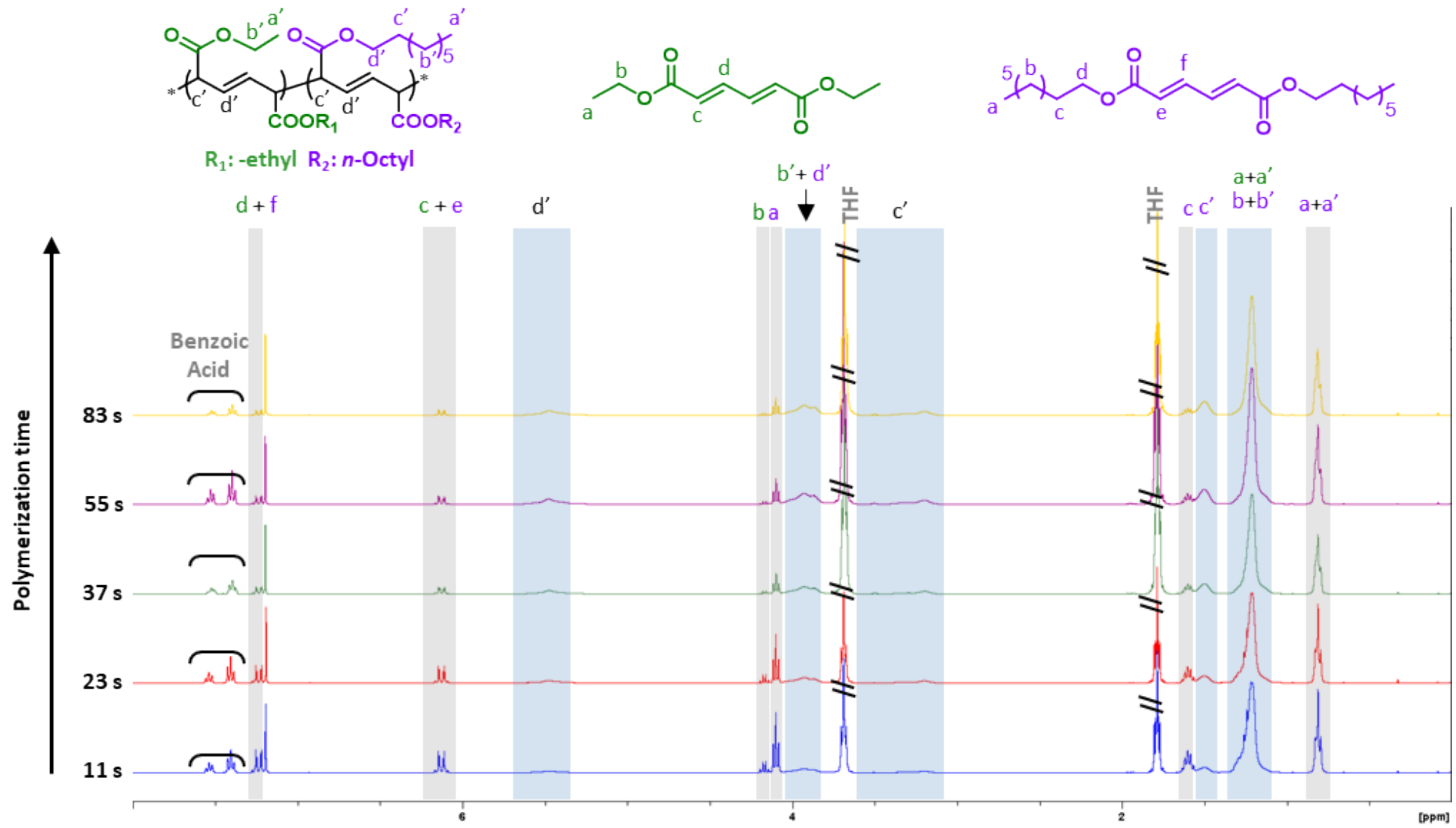

Figure S11. Monitoring study by ¹H NMR of one-pot copolymerization of DEM (green) and DOM (purple) in THF using P_4-*t*-Bu as catalyst (Table 1, entry 5), showing the changes in the characteristic peaks of the monomer after the start of the polymerization: 11 s (blue spectrum), 23 s (red spectrum), 37 s (green spectrum), 55 s (purple spectrum) and 83 s (yellow spectrum).

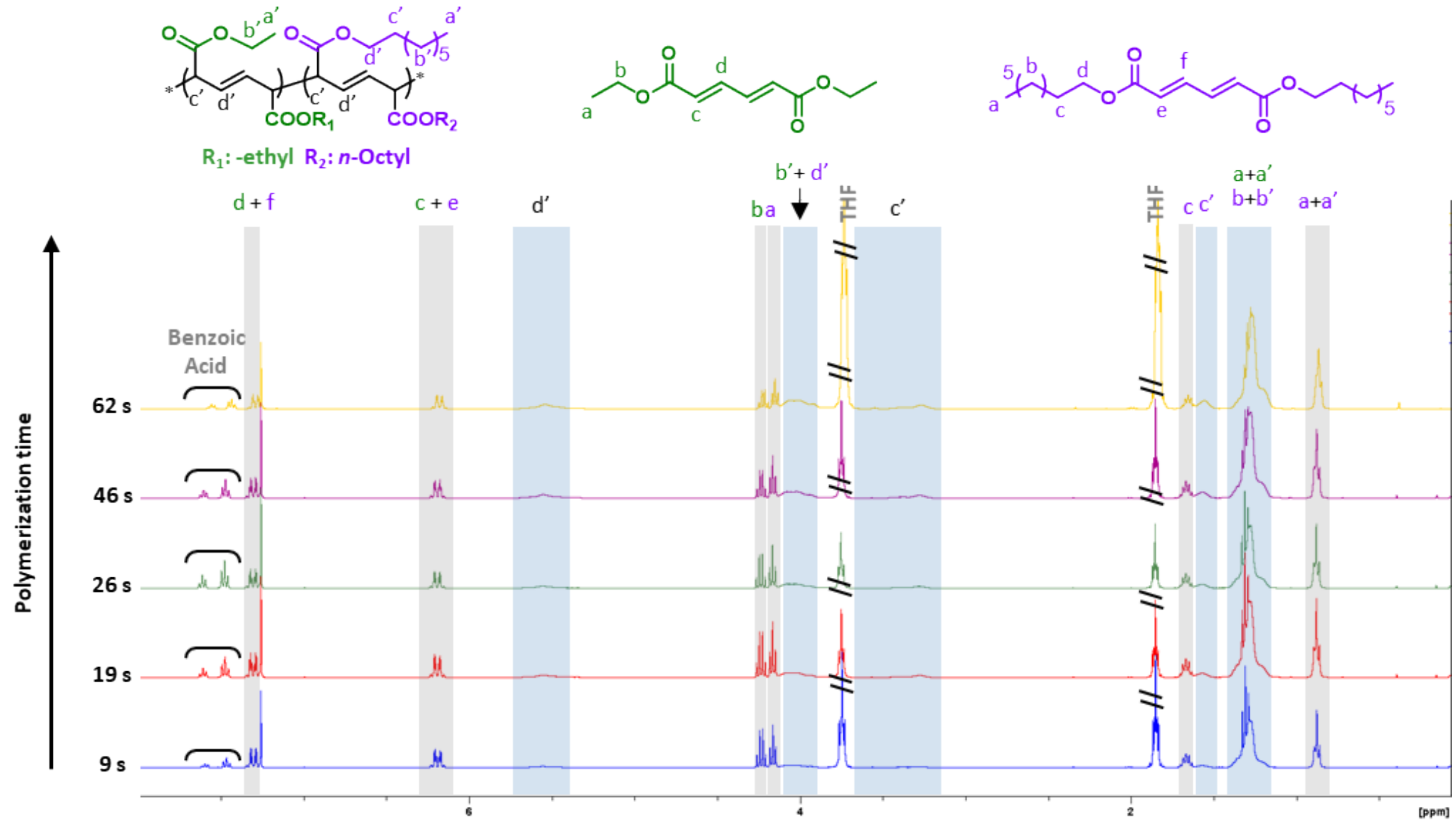

Figure S12. Monitoring study by ^{1}H NMR of one-pot copolymerization of DEM (green) and DOM (purple) in THF using P_4-*t*-Bu as catalyst (Table 1, entry 6), showing the changes in the characteristic peaks of the monomer after the start of the polymerization: 9 s (blue spectrum), 19 s (red spectrum), 26 s (green spectrum), 46 s (purple spectrum) and 62 s (yellow spectrum).

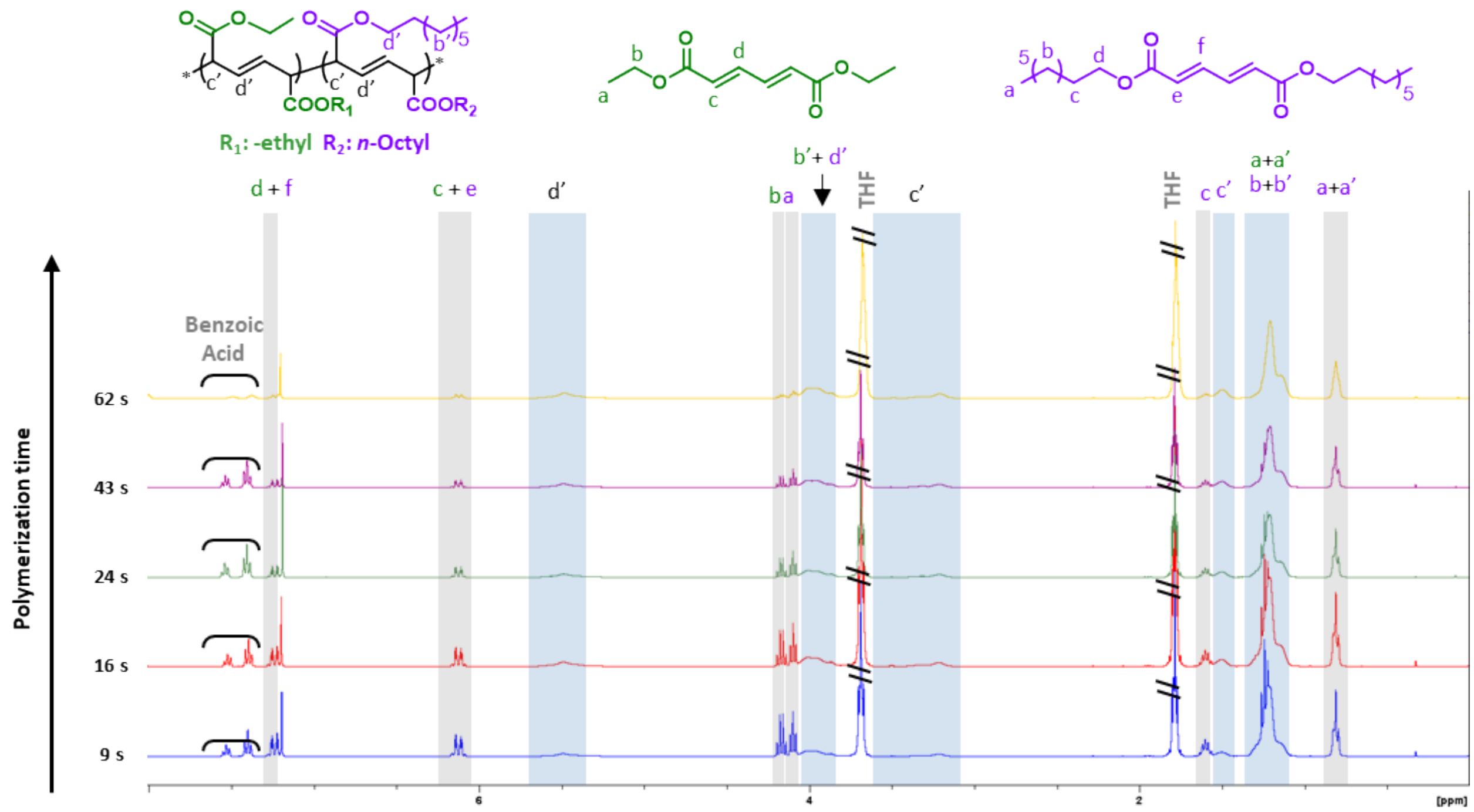

Figure S13. Monitoring study by ¹H NMR of one-pot copolymerization of DEM (green) and DOM (purple) in THF using P_4-*t*-Bu as catalyst (Table 1, entry 7), showing the changes in the characteristic peaks of the monomer after the start of the polymerization: 9 s (blue spectrum), 16 s (red spectrum), 24 s (green spectrum), 43 s (purple spectrum) and 62 s (yellow spectrum).

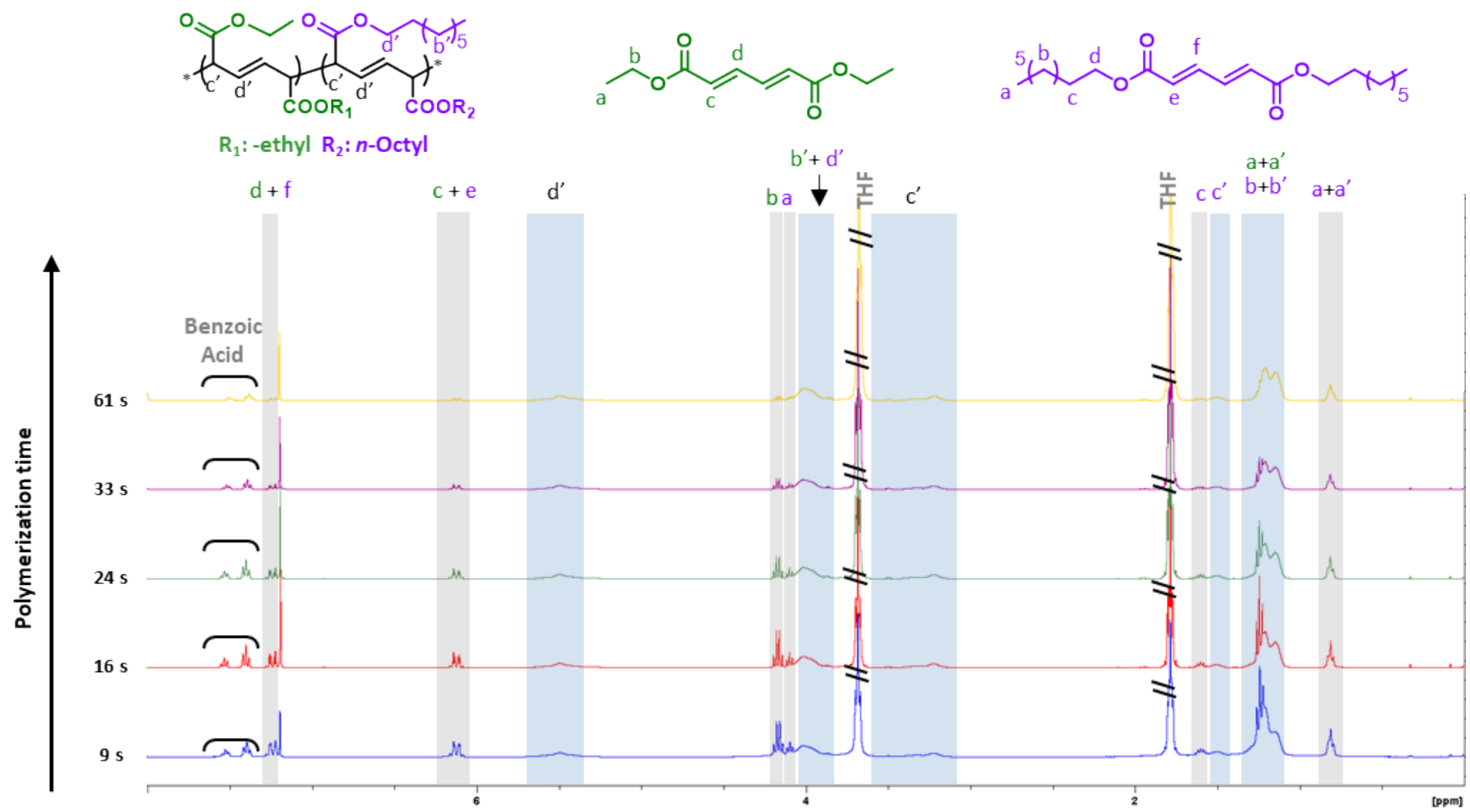

Figure S14. Monitoring study by ^{1}H NMR of one-pot copolymerization of DEM (green) and DOM (purple) in THF using P_4-*t*-Bu as catalyst (Table 1, entry 8), showing the changes in the characteristic peaks of the monomer after the start of the polymerization: 9 s (blue spectrum), 16 s (red spectrum), 24 s (green spectrum), 33 s (purple spectrum) and 61 s (yellow spectrum).

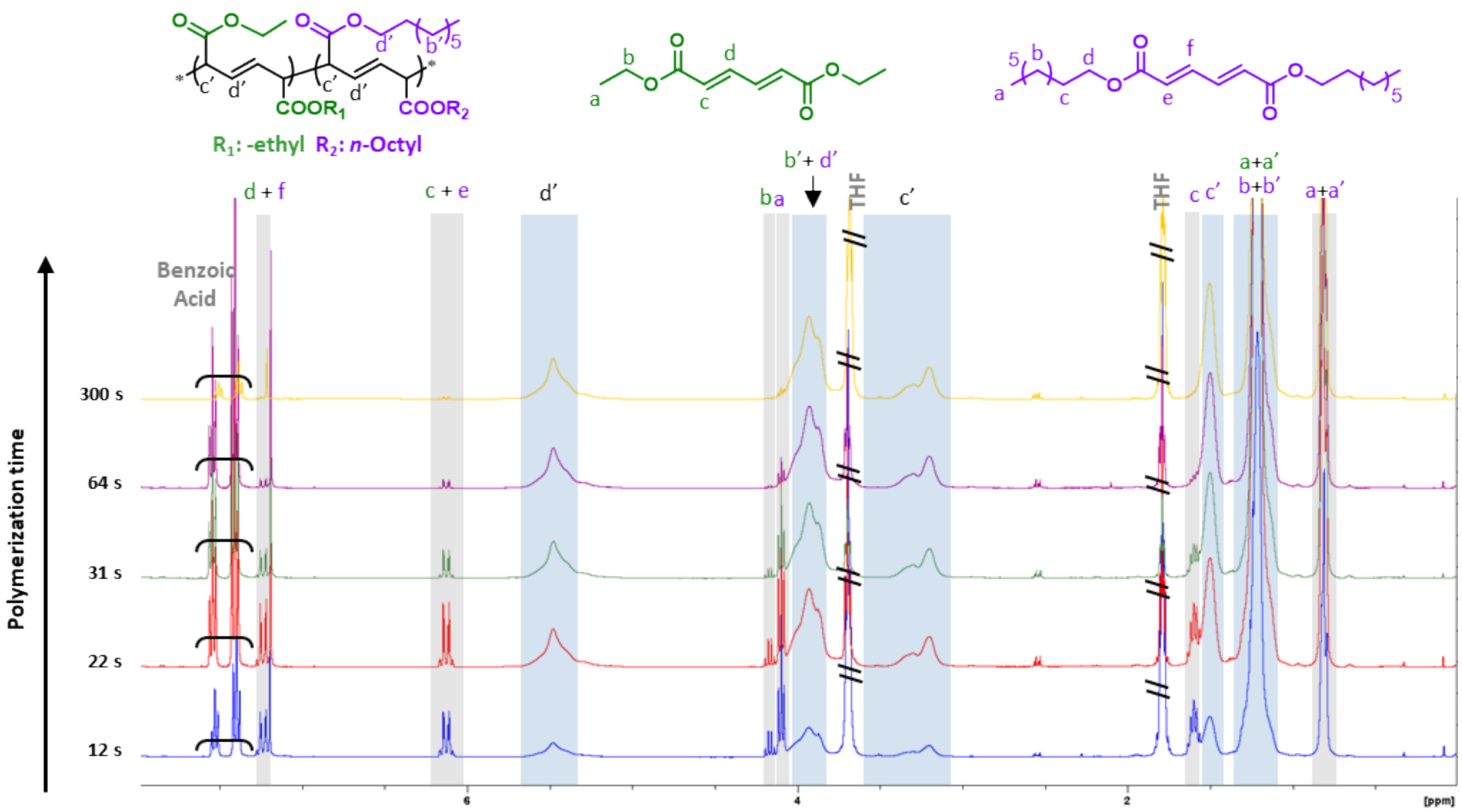

Figure S15. Monitoring study by ^{1}H NMR of one-pot copolymerization of DEM (green) and DOM (purple) in THF using P_2-*t*-Bu as catalyst (Table 1, entry 9), showing the changes in the characteristic peaks of the monomer after the start of the polymerization: 12 s (blue spectrum), 22 s (red spectrum), 31 s (green spectrum), 64 s (purple spectrum) and 300 s (yellow spectrum).

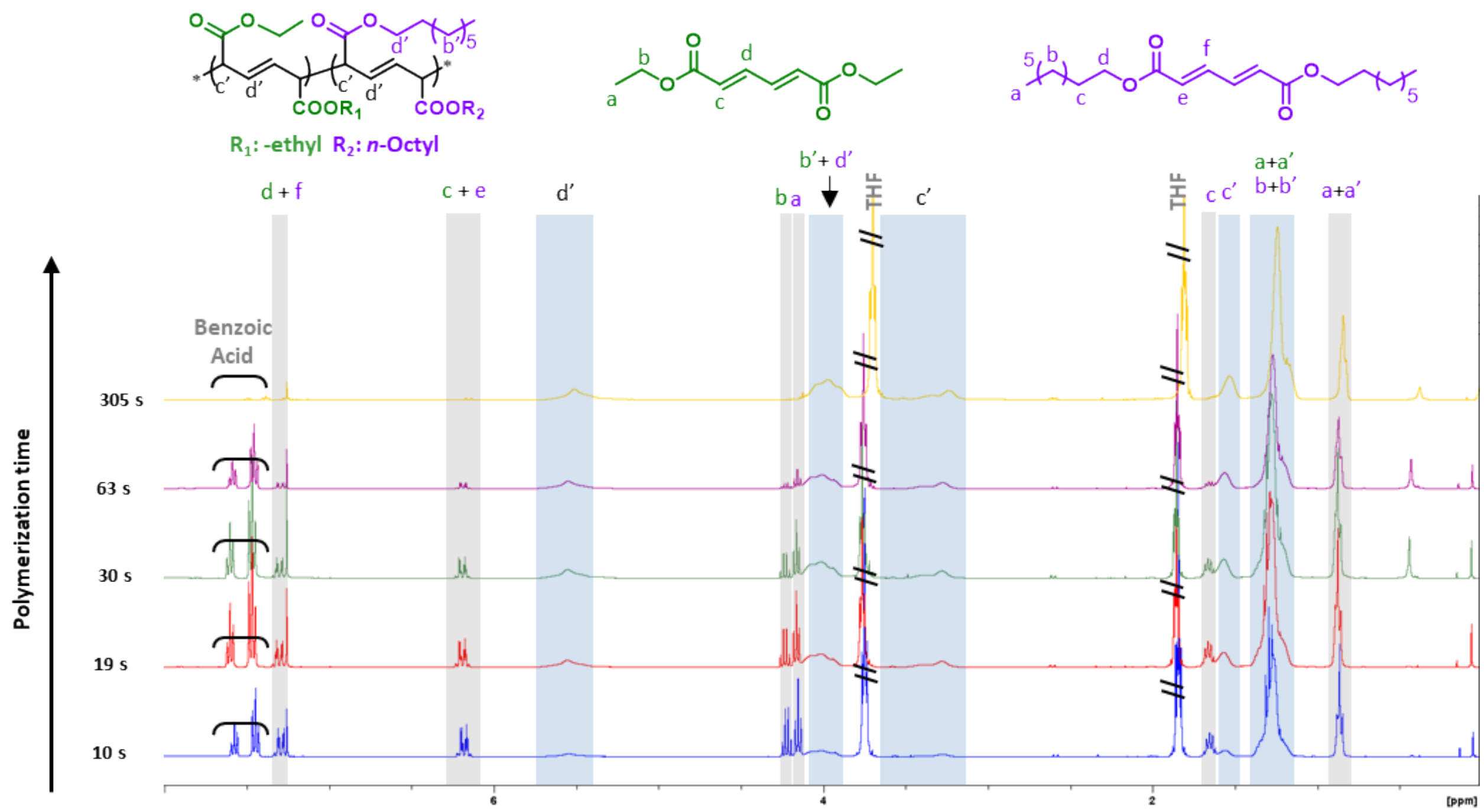

Figure S16. Monitoring study by ¹H NMR of one-pot copolymerization of DEM (green) and DOM (purple) in THF using P_2-*t*-Bu as catalyst (Table 1, entry 10), showing the changes in the characteristic peaks of the monomer after the start of the polymerization: 10 s (blue spectrum), 19 s (red spectrum), 30 s (green spectrum), 63 s (purple spectrum) and 305 s (yellow spectrum).

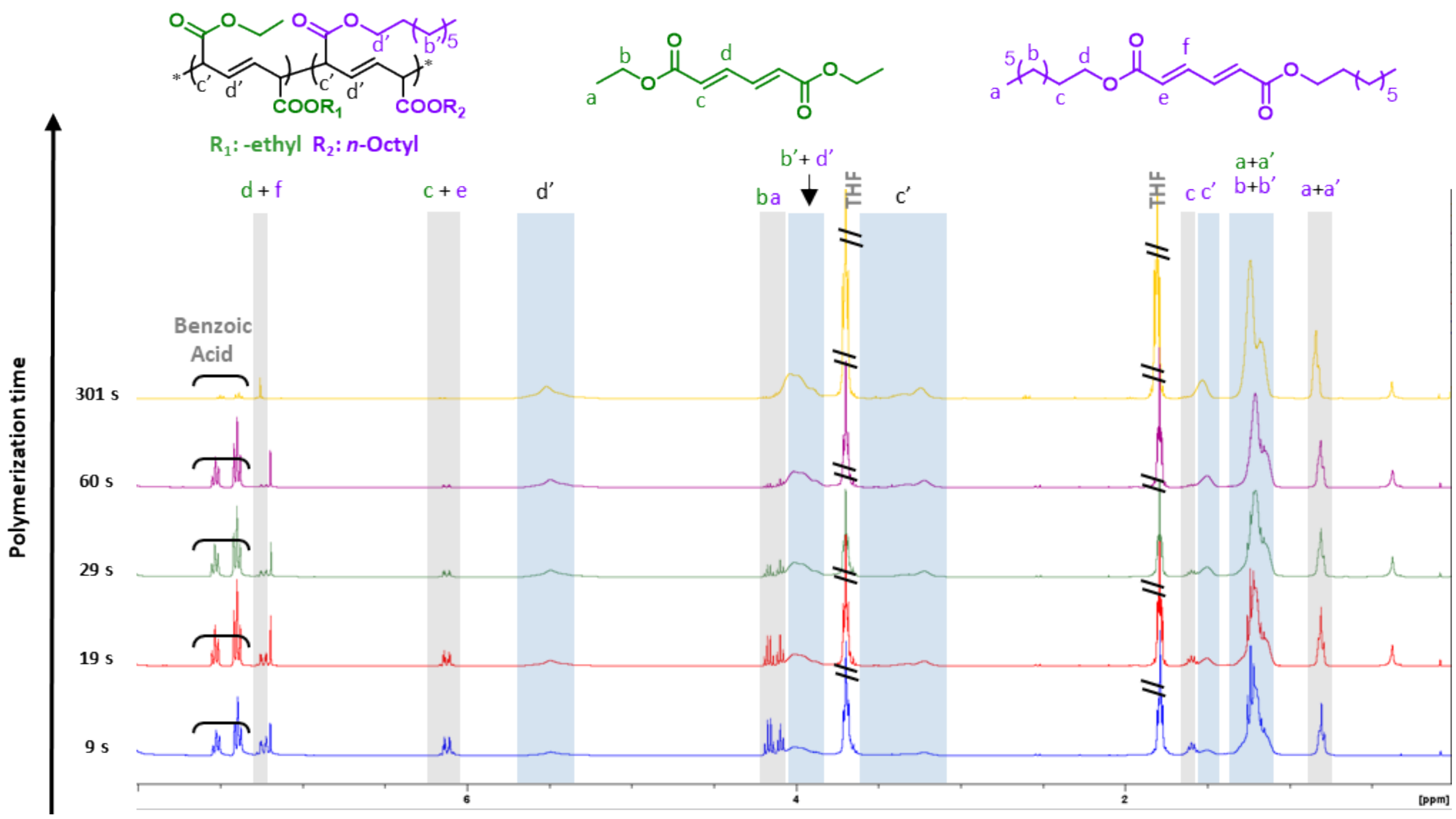

Figure S17. Monitoring study by ¹H NMR of one-pot copolymerization of DEM (green) and DOM (purple) in THF using P_2-*t*-Bu as catalyst (Table 1, entry 11), showing the changes in the characteristic peaks of the monomer after the start of the polymerization: 9 s (blue spectrum), 19 s (red spectrum), 29 s (green spectrum), 60 s (purple spectrum) and 301 s (yellow spectrum).

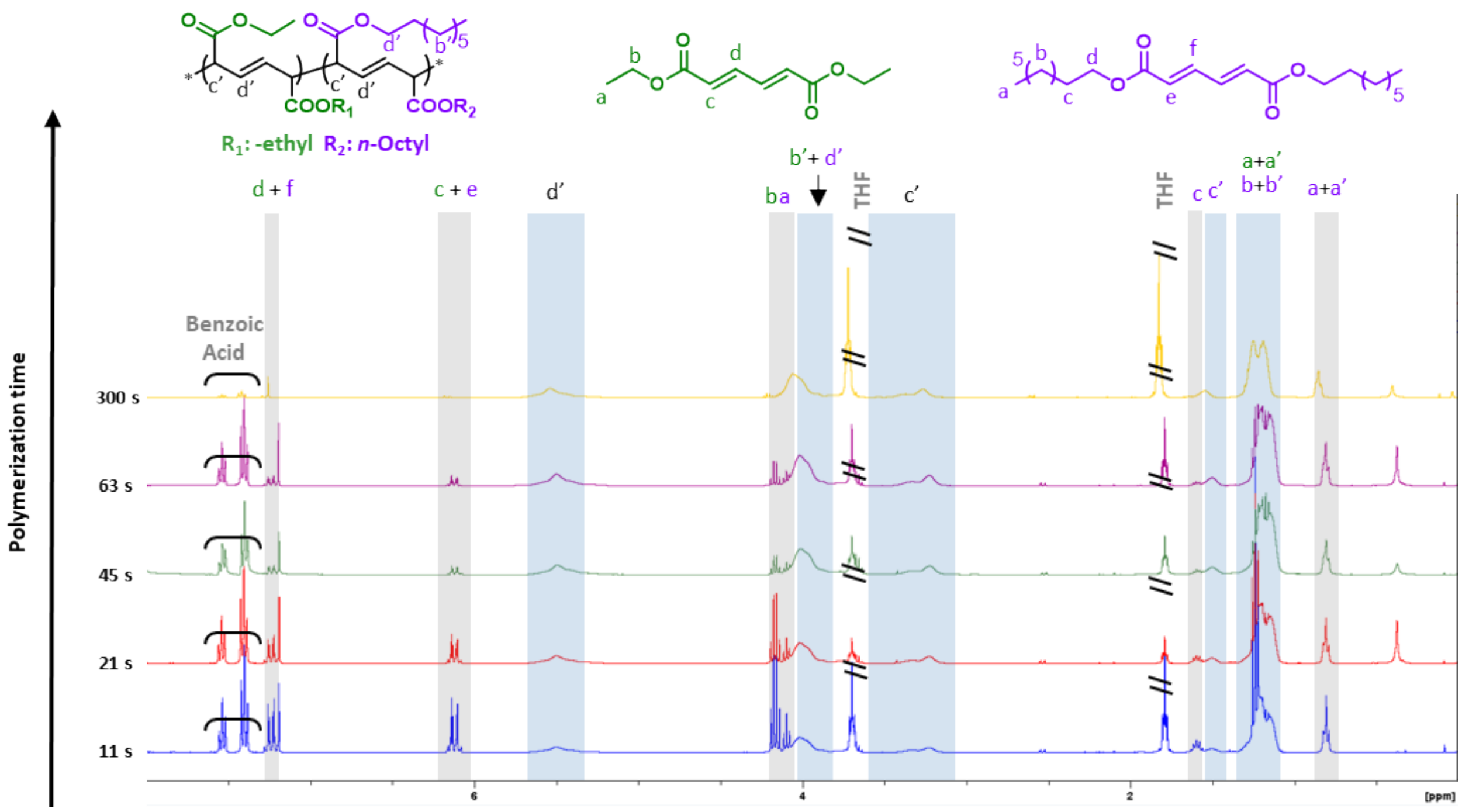

Figure S18. Monitoring study by ¹H NMR of one-pot copolymerization of DEM (green) and DOM (purple) in THF using P_2-*t*-Bu as catalyst (Table 1, entry 12), showing the changes in the characteristic peaks of the monomer after the start of the polymerization: 11 s (blue spectrum), 21 s (red spectrum), 45 s (green spectrum), 63 s (purple spectrum) and 300 s (yellow spectrum).

- **Kelen-Tüdos linearization method :**[2]

$$\square = \left(r_1 + \frac{r_2}{\square}\right)\square\,\square - \frac{r_2}{\square} \qquad \text{(S8)}$$

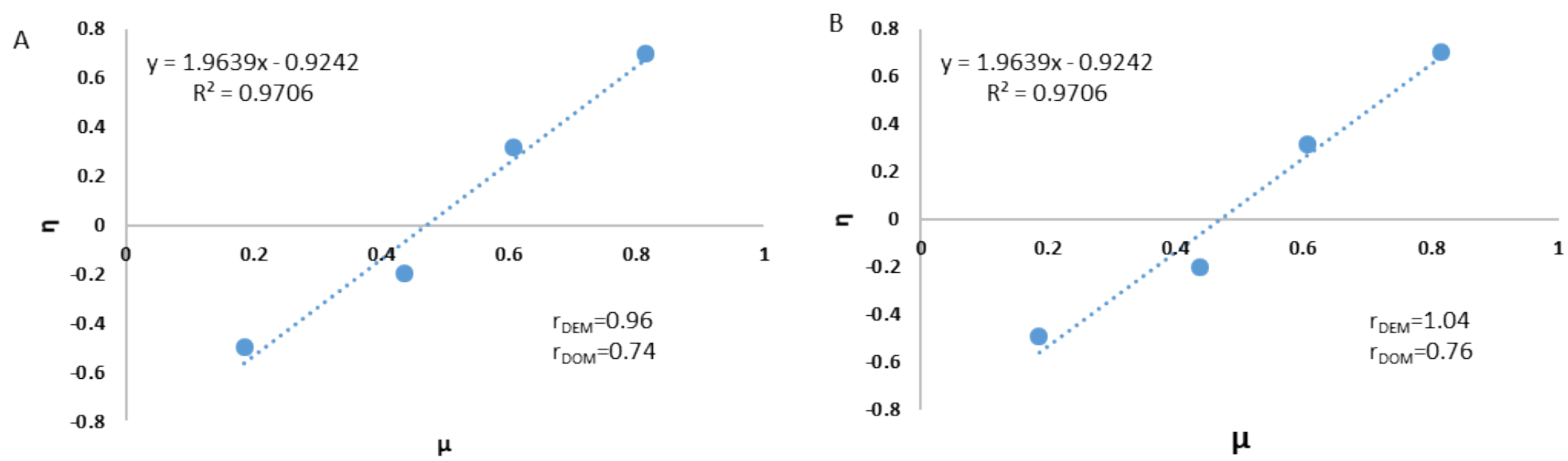

Figure S19. Calculation of reactivity ratios through Kelen-Tüdos approach for **A.** THF-P_4-*t*-Bu systems, **B.** THF-P_2-*t*-Bu.

- **Integrated form of Mayo-Lewis equation proposed by Meyer-Lowry:**[3]

$$conv. = 1 - \left(\frac{f_A}{f_A^0}\right)^{r_B/(1-r_B)} \left(\frac{1-f_A}{1-f_A^0}\right)^{r_A/(1-r_A)} \left(\frac{f_A(2-r_A-r_B)+r_B-1}{f_A^0(2-r_A-r_B)+r_B-1}\right)^{r_A r_B/[(1-r_A)(1-r_B)]} \qquad \text{(S9)}$$

The confidence intervals were calculated by minimizing the sum of squares of the residuals obtained from fitting the Meyer-Lowry equation. Multiple initial monomer feeds were studied because each individual reaction provides estimates of the reactivity ratios through fitting of Equation S9. By varying the initial monomer feed, multiple estimates of the reactivity ratios can therefore be obtained. The reactivity ratios were then determined by simultaneously minimizing the sum of squares of the residuals from all experiments, allowing convergence to a common solution and enabling the corresponding confidence intervals to be calculated. This approach allows the reactivity ratios to be determined using experimental data at relatively high conversion. However, to avoid potential overestimation, experimental data were excluded from the reactivity-ratio calculation when either the monomer conversion or the global conversion exceeded 95%.

The reactivity ratios were determined by minimizing the sum of squared residuals (SS), and the corresponding confidence intervals were calculated according to the VSSRS statistical procedure described by Van den Brink *et al.*[5] No replicate experiments were performed under identical experimental conditions. Each experimental condition corresponds to an independent polymerization experiment. Therefore, the confidence intervals reported for the reactivity ratios arise from the statistical analysis of the nonlinear least-squares fit and should not be interpreted as an estimate of experimental repeatability.

DSC copolymer characterization:

- **Fox equation:**

The Fox-equation was used to determine the theoretical glass transition temperature to confirm the presence of statistical copolymers.[4]

$$\frac{1}{T_g} = \sum \frac{w_i}{T_{(g,i)}} \qquad \text{(S10)}$$

where w_i represents the weight fraction of each comonomer unit.

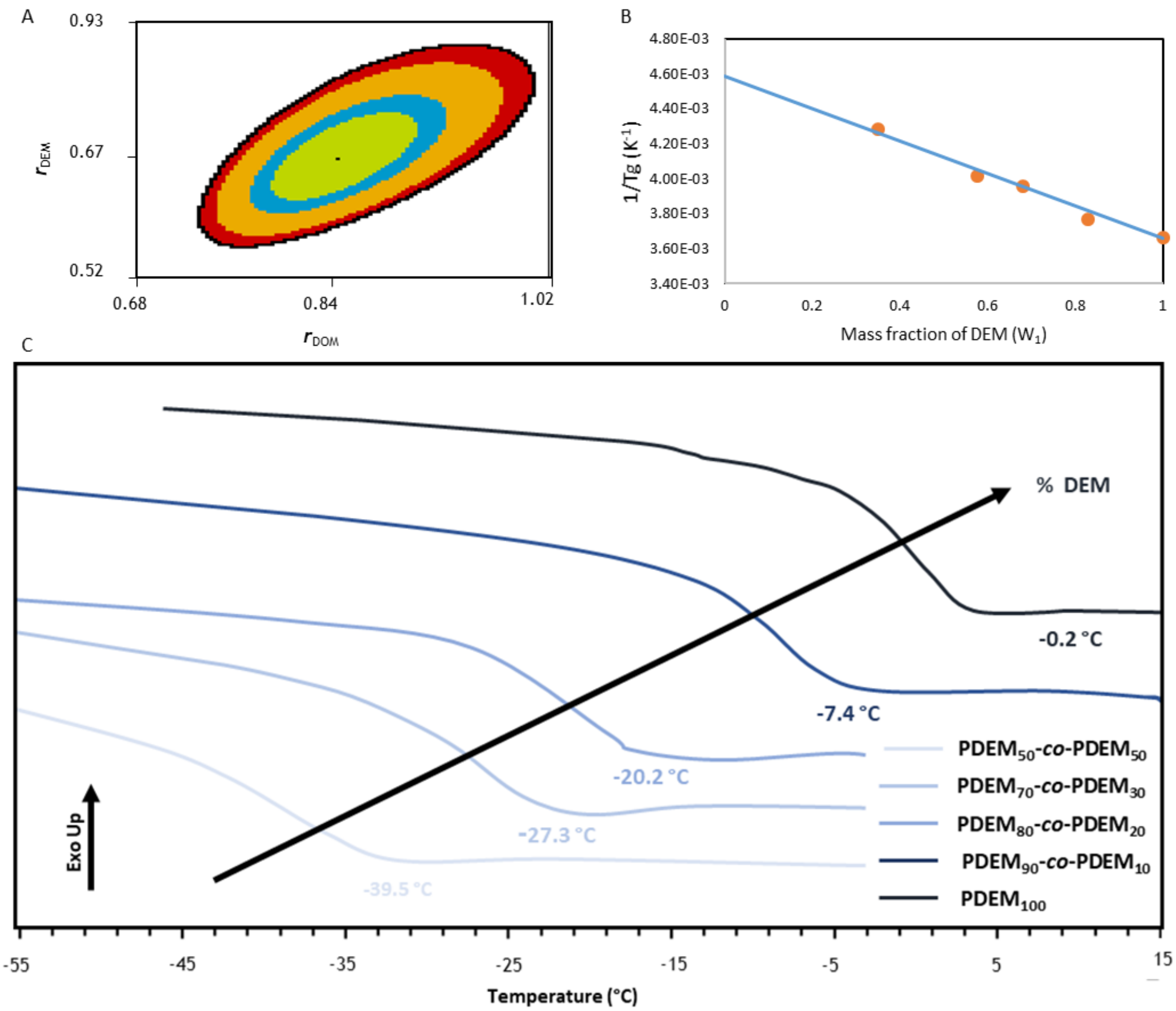

Figure S20. A. 95% joint confidence interval (JCI) for reactivity ratios in toluene and using P_4-*t*-Bu as catalyst, $r_{DEM} = 0.67$ and $r_{DOM} = 0.84$, colored regions represent 50%, 70% and 90% JCIs. **B.** Glass transition temperature of the copolymers calculated by Fox equation (solid line) with the experimental values (solid dots). **C.** Thermograms of second heating run obtained by DSC, showing an increase in the glass transition temperature as the composition in DEM increases.

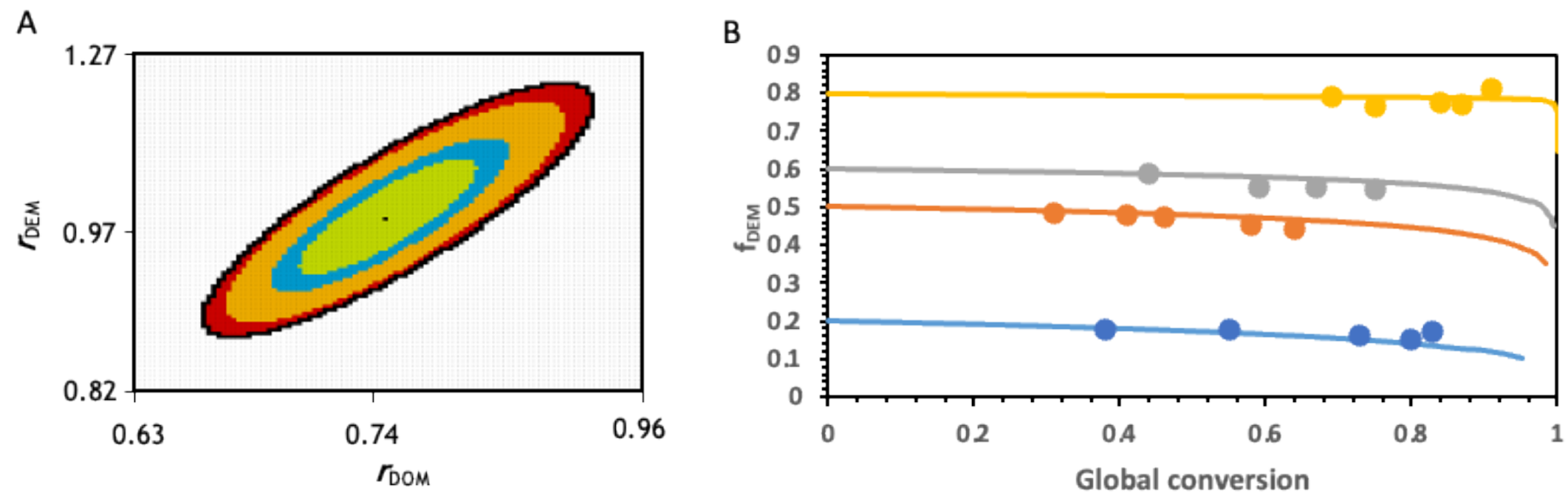

Figure S21. A. 95% joint confidence interval (JCI) for reactivity ratios in THF and using P_4-*t*-Bu as catalyst, r_{DEM} = 0.97 and r_{DOM} = 0.74, colored regions represent 50%, 70% and 90% JCIs. **B.** Prediction of the comonomer ratio (solid lines) as a function of conversion vs experimental points (solid dots), for $f_{DEM,0}$ = 0.80, 0.60, 0.40 and 0.20 (top to bottom respectively) for the system THF-P_4-*t*-Bu.

Table S1. Reactivity ratios for the copolymerization of DEM and DOM in different solvents and with various catalysts.

Solvent	Catalyst	Monomer 1	Monomer 2	r_1	r_2
Toluene	P_4-*t*-Bu	DEM	DOM	0.67 ± 0.22	0.84 ± 0.16
THF	P_4-*t*-Bu	DEM	DOM	0.97 ± 0.25	0.74 ± 0.18
THF	P_2-*t*-Bu	DEM	DOM	1.08 ± 0.26	0.84 ± 0.24
Isododecane*	P_4-*t*-Bu	DEM	DOM	1.86 ± 0.93	0.58 ± 0.28

*Isododecane was removed under vacuum. Residual traces of isododecane were subsequently removed by dissolution in pentane followed by evaporation under vacuum. Note that even small amounts of residual isododecane can significantly affect the calculated DOM conversion, potentially introducing larger deviations in the conversion values.

O-GTcP using isododecane as solvent:

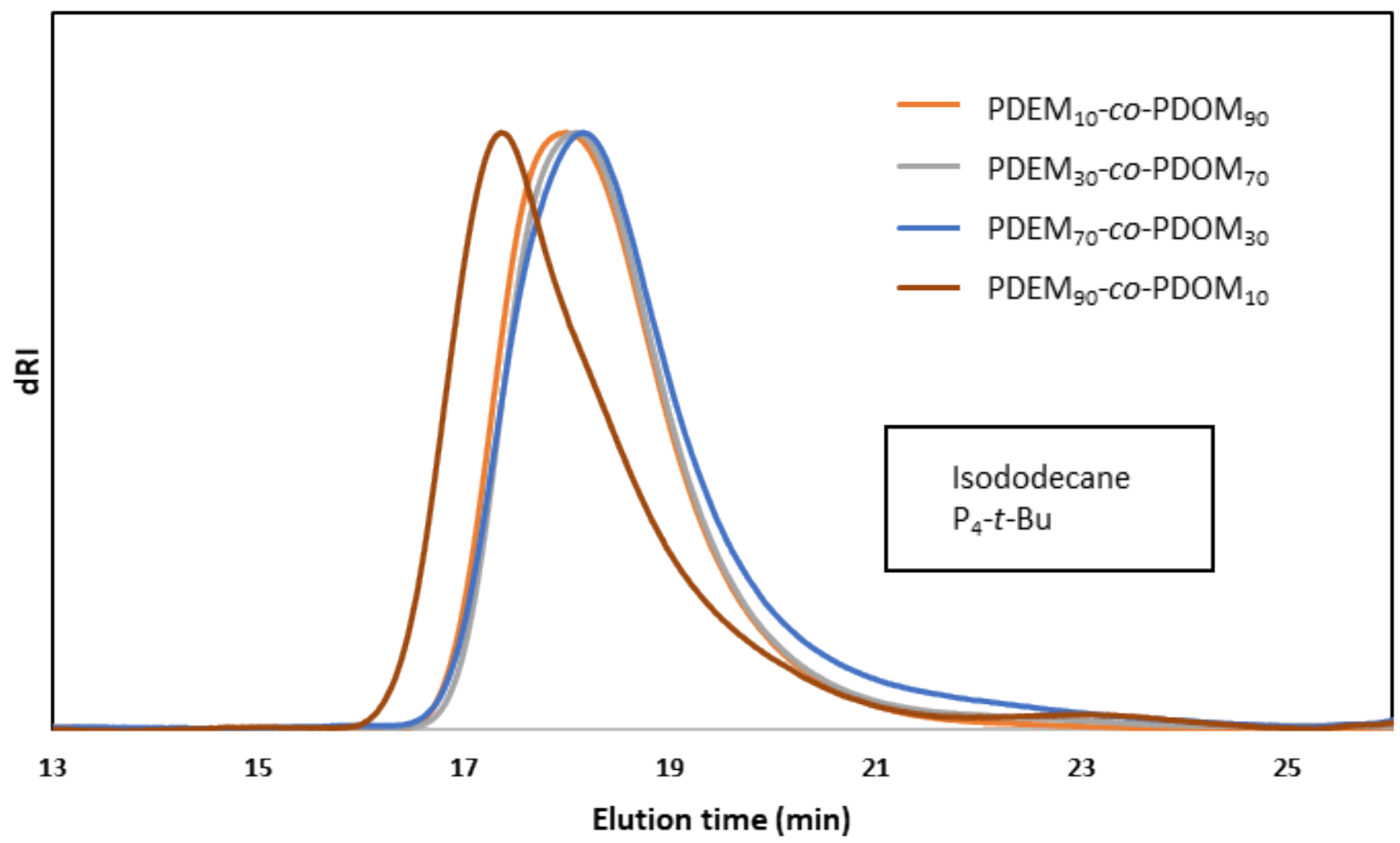

Figure S22. SEC traces comparison of different PDEM-*co*-PDOM compositions (Table 2, Entries 1,3,7,9) obtained after DEM and DOM O-GTcP using P_4-*t*-Bu catalyst in isododecane.

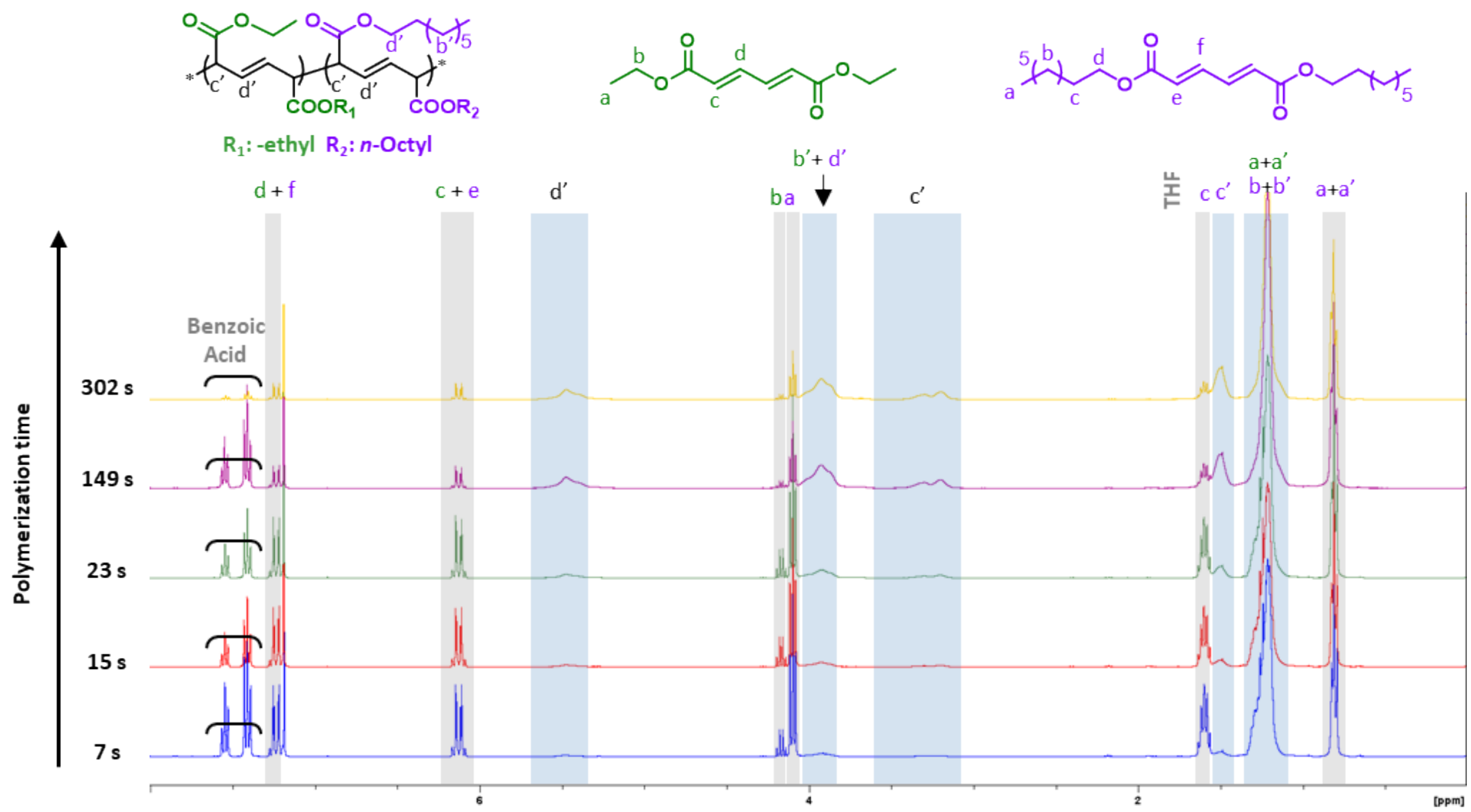

Figure S23. Monitoring study by ¹H NMR of one-pot copolymerization of DEM (green) and DOM (purple) in isododecane using P_4-*t*-Bu as catalyst (Table 2, entry 2 conditions), showing the changes in the characteristic peaks of the monomer after the start of the polymerization: 7 s (blue spectrum), 15 s (red spectrum), 23 s (green spectrum), 149 s (purple spectrum) and 302 s (yellow spectrum).

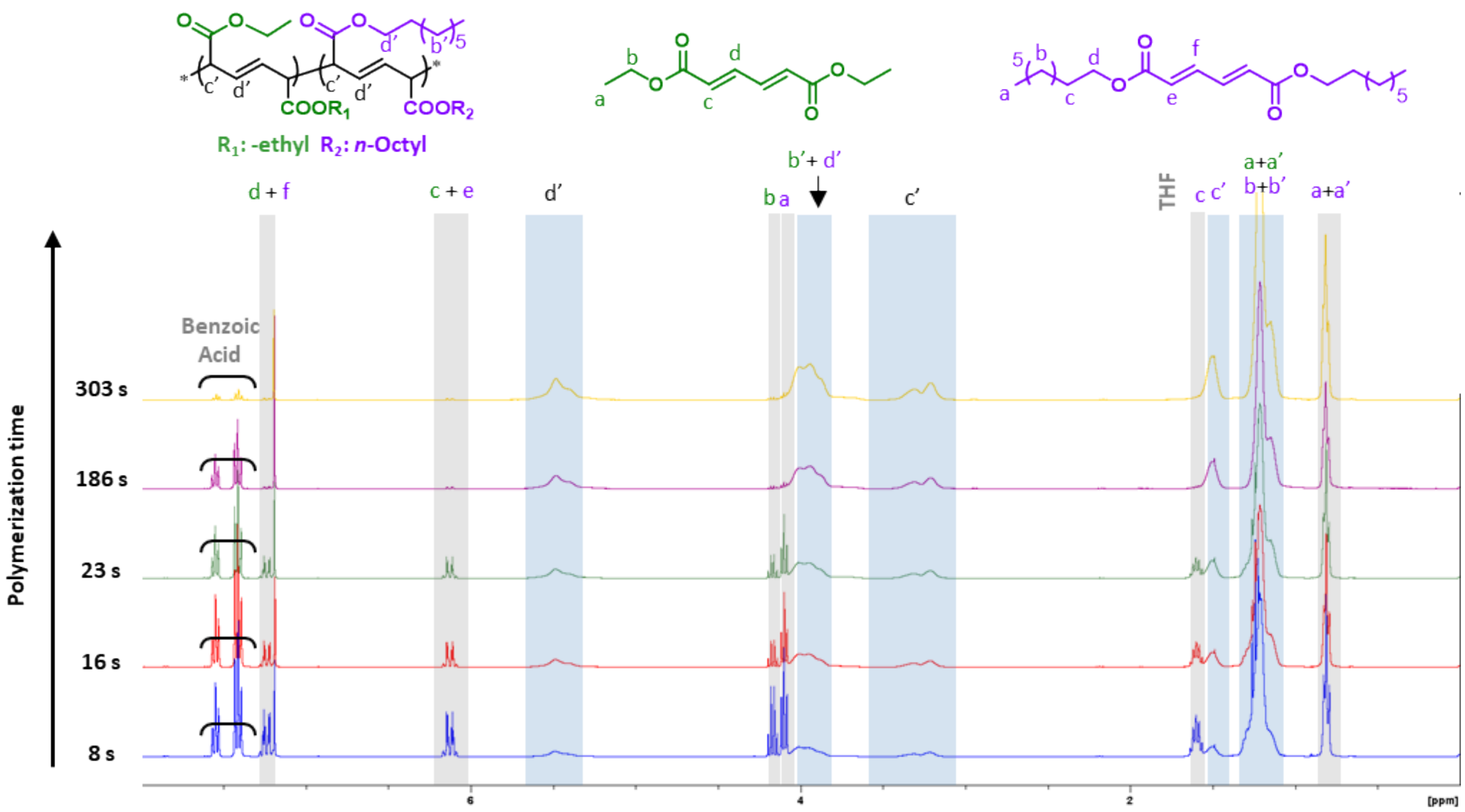

Figure S24. Monitoring study by ¹H NMR of one-pot copolymerization of DEM (green) and DOM (purple) in isododecane using P_4-*t*-Bu as catalyst (Table 2, entry 4 conditions), showing the changes in the characteristic peaks of the monomer after the start of the polymerization: 8 s (blue spectrum), 16 s (red spectrum), 23 s (green spectrum), 186 s (purple spectrum) and 303 s (yellow spectrum).

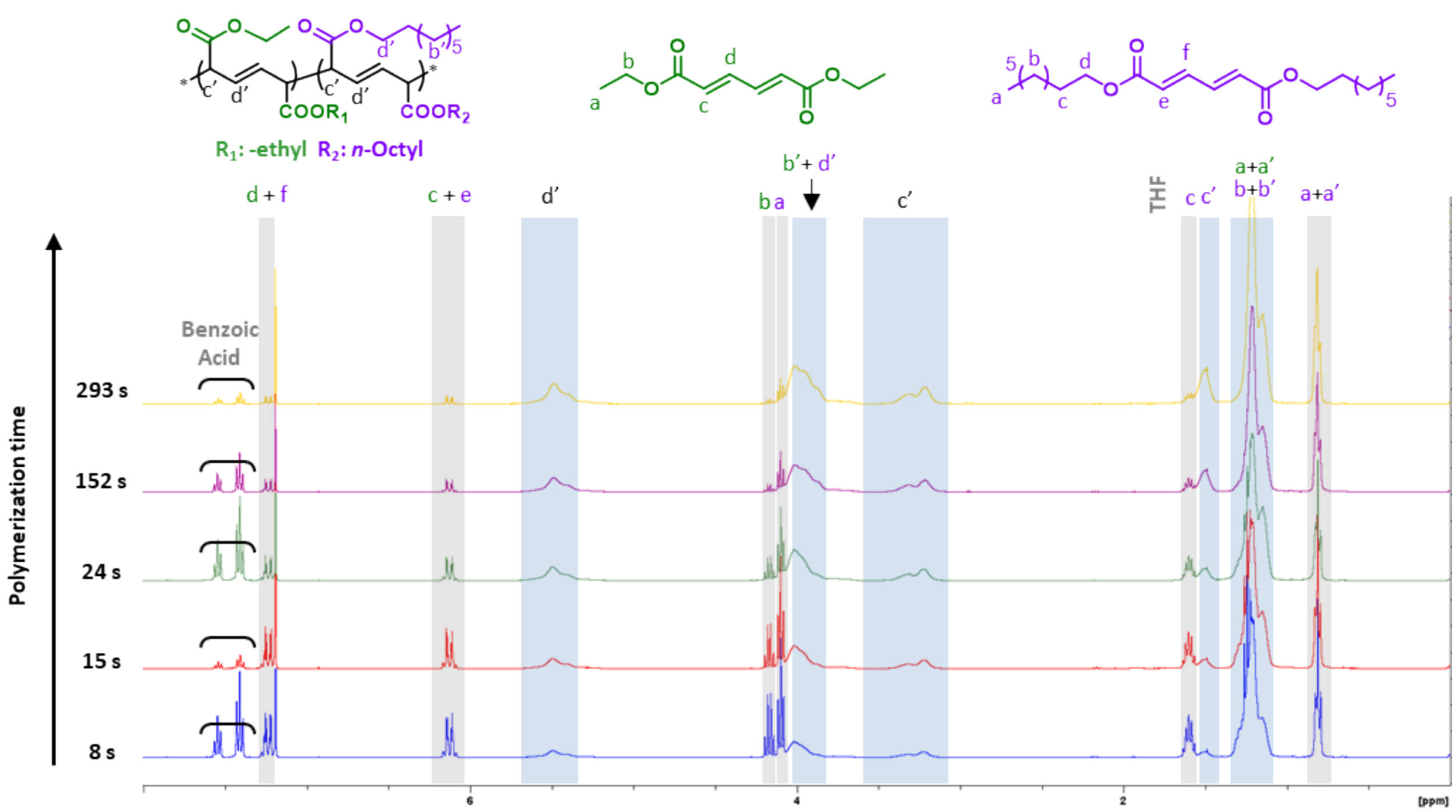

Figure S25. Monitoring study by ^{1}H NMR of one-pot copolymerization of DEM (green) and DOM (purple) in isododecane using P_4-*t*-Bu as catalyst (Table 2, entry 5 conditions), showing the changes in the characteristic peaks of the monomer after the start of the polymerization: 8 s (blue spectrum), 15 s (red spectrum), 24 s (green spectrum), 152 s (purple spectrum) and 293 s (yellow spectrum).

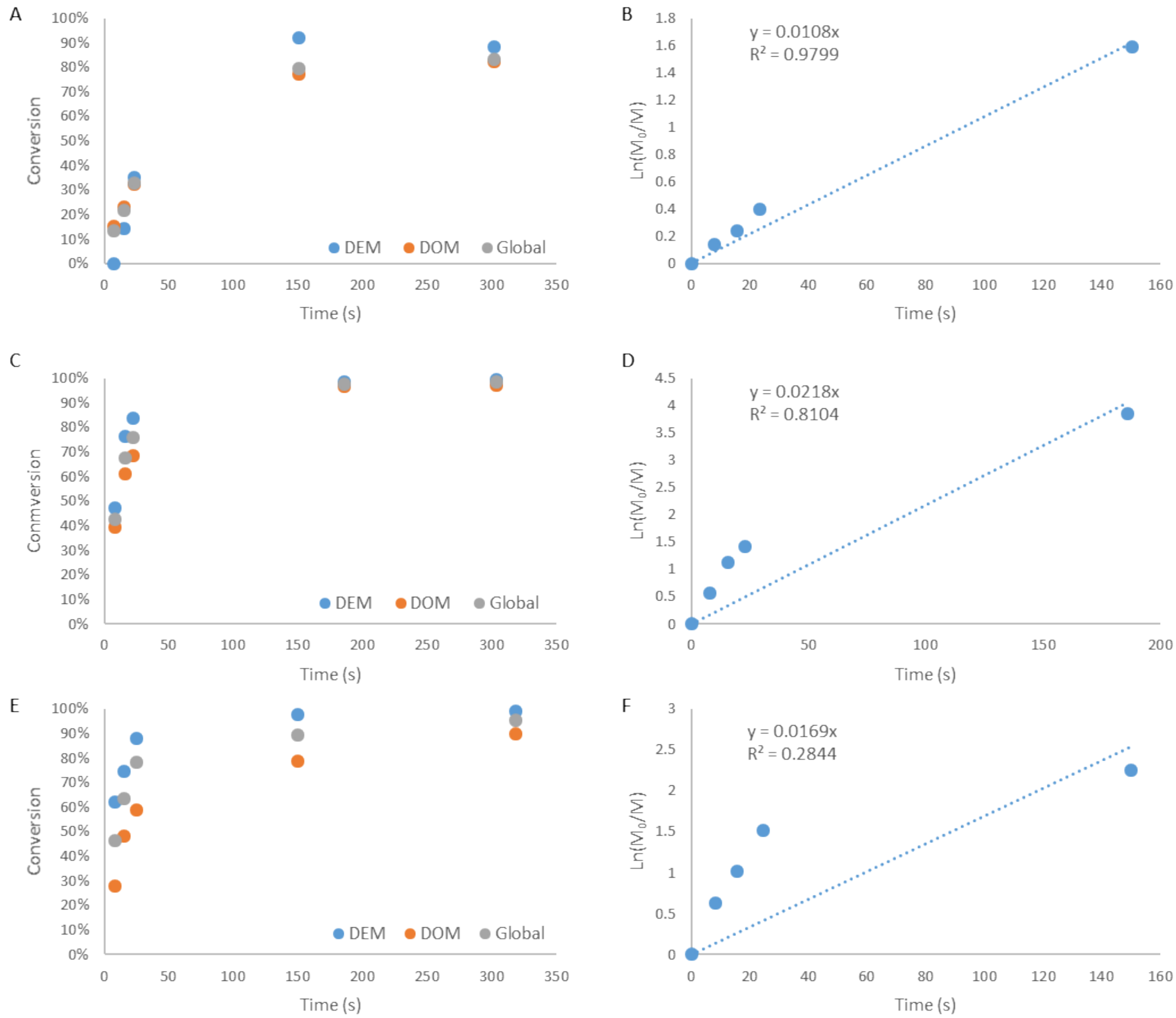

Figure S26. Monitoring of copolymerization using isododecane as solvent and P_4-*t*-Bu as catalyst. **A.** Conversion of DEM and DOM during the synthesis of $PDEM_{20}$-*co*-$PDOM_{80}$. **B.** Ln($[M]_0/[M]$) as a function of time, where $[M]_0$ is the initial concentration of DEM and DOM together, and M is the concentration of DEM and DOM at the time of the sampling. **C.** Conversion of DEM and DOM during the synthesis of $PDEM_{50}$-*co*-$PDOM_{50}$. **D.** Ln($[M]_0/[M]$) as a function of time. **E.** Conversion of DEM and DOM during the synthesis of $PDEM_{60}$-*co*-$PDOM_{40}$. **F.** Ln($[M]_0/[M]$) as a function of time.

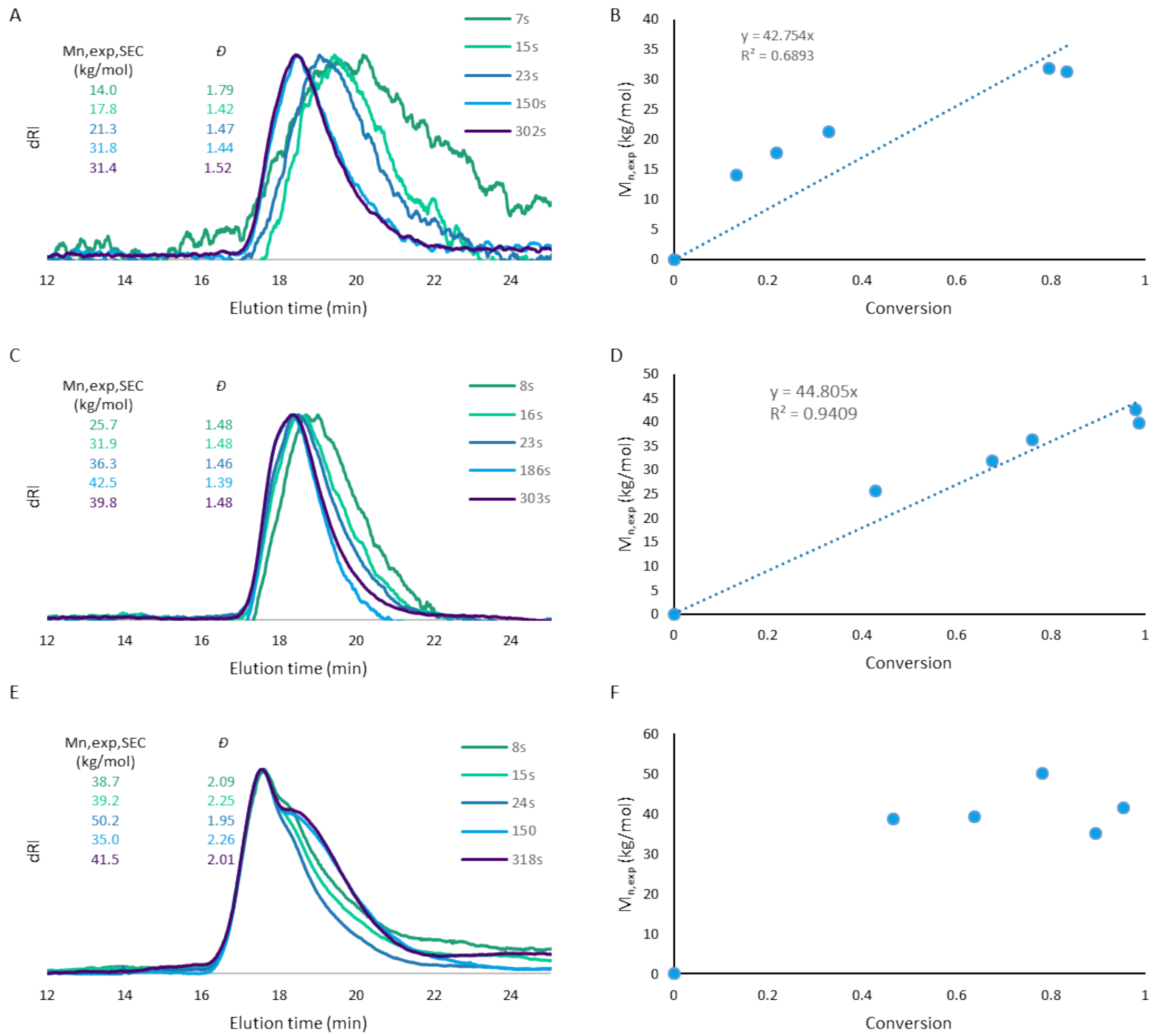

Figure S27. Molecular Weight analysis done by SEC for entries using isododecane as solvent and P_4-*t*-Bu as catalyst. **A.** Evolution of molecular weight distribution with the progress of the polymerization of $PDEM_{20}$-*co*-$PDOM_{80}$. **B.** Molecular weight as a function of global conversion to examine the presence of transfer reactions. **C.** Evolution of molecular weight distribution with the progress of the polymerization of $PDEM_{50}$-*co*-$PDOM_{50}$. **D.** Molecular weight as a function of global conversion to examine the presence of transfer reactions. **E.** Evolution of molecular weight distribution with the progress of the polymerization of $PDEM_{60}$-*co*-$PDOM_{40}$. **F.** Molecular weight as a function of global conversion to examine the presence of transfer reactions.

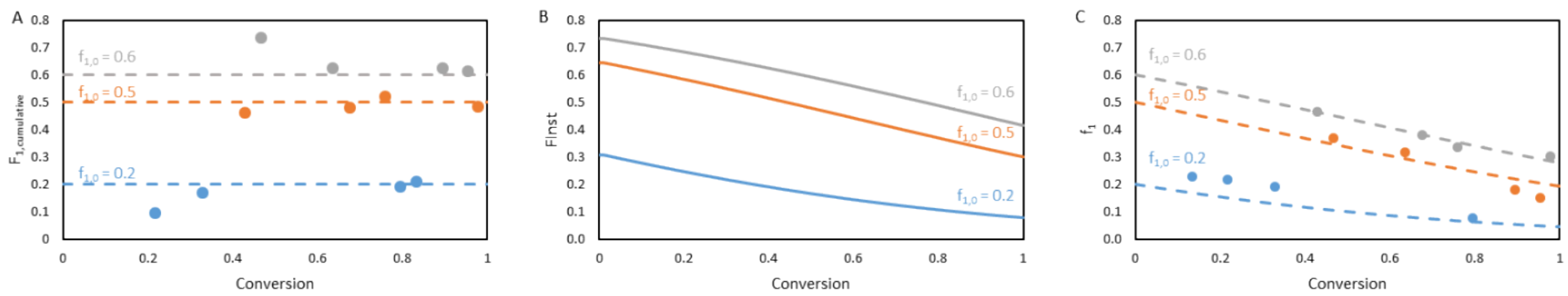

Figure 28. A. $F_{DEM,cumulative}$ *vs* global conversion of the copolymerization of DEM and DOM in isododecane using P_4-*t*-Bu as catalyst with initial feed composition of DEM ($f_{1,0}$) equal to 0.2 (blue), 0.5 (orange), 0.6 (grey). **B.** Theoretical instantantenous polymer composition ($F_{DEM,inst}$) vs global conversion calculated using reactivity ratios of r_{DEM} = 1.89 ± 0.93 and r_{DOM} = 0.58 ± 0.28. **C**. f_{DEM} *vs* global conversion of the copolymerization of DEM and DOM in isododecane using P_4-*t*-Bu as catalyst with initial feed composition of DEM ($f_{1,0}$) equal to 0.2 (blue), 0.5 (orange), 0.6 (grey).

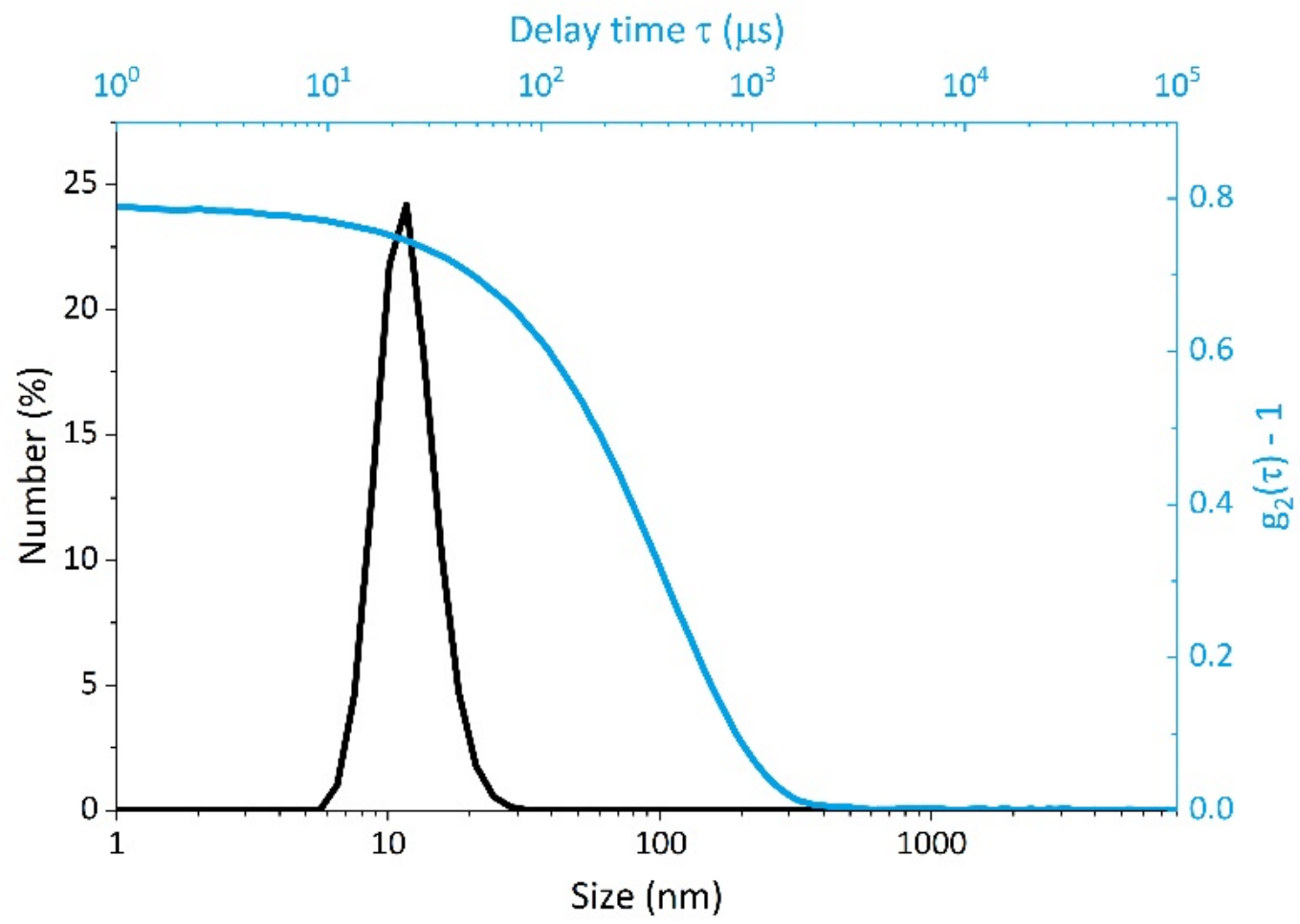

Figure S29. DLS size distribution showing the hydrodynamic diameter as a function of particle number (black) and the corresponding correlation function (blue) for $PDEM_{60}$-*co*-$PDOM_{40}$ nanoparticles.

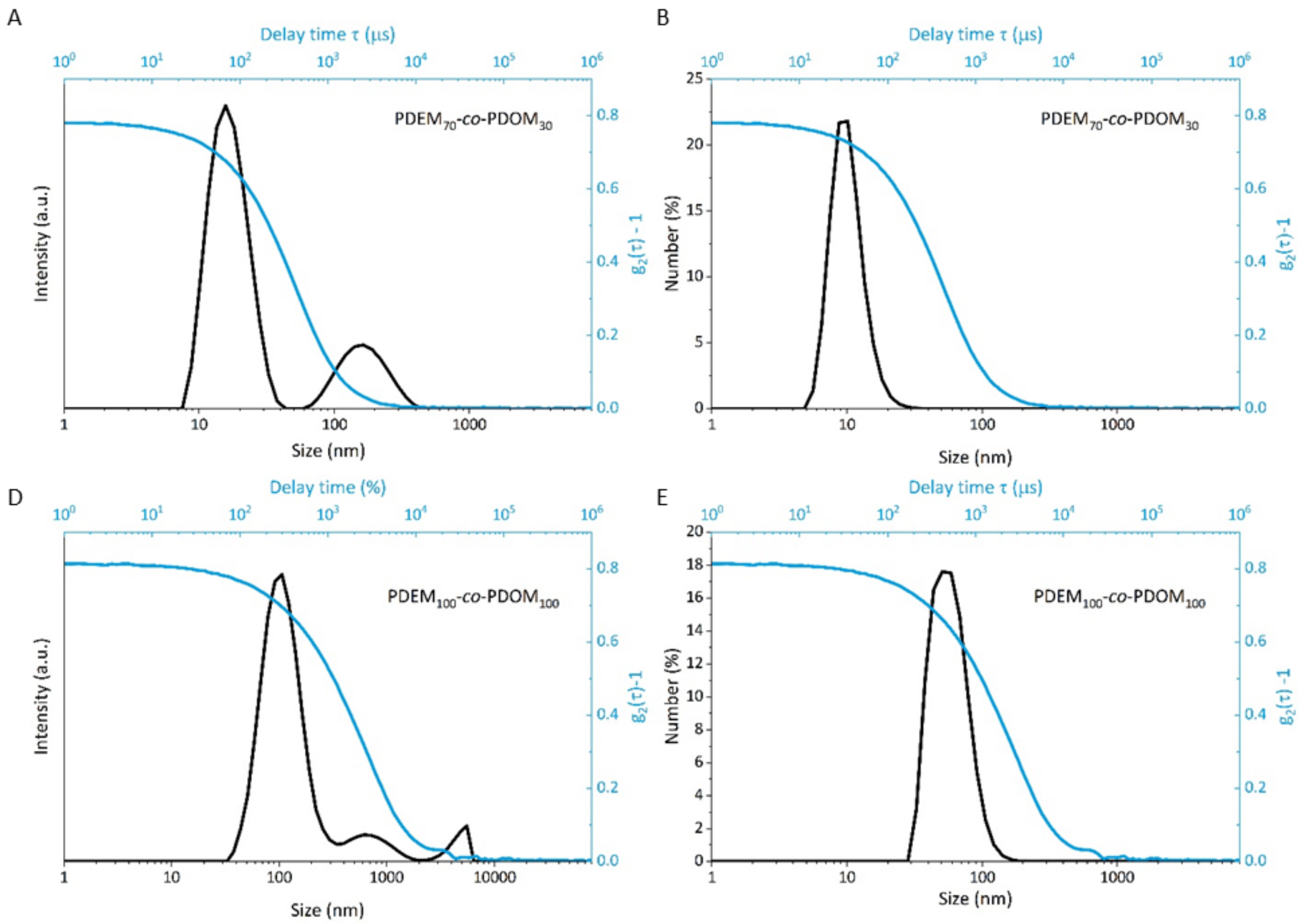

Figure S30. A. DLS size distribution showing the hydrodynamic diameter as a function of scattered intensity (black) and the corresponding correlation function (blue) for particles of PDEM$_{70}$-*co*-PDOM$_{30}$ **B.** DLS size distribution showing the hydrodynamic diameter as a function of particle number (black) and the corresponding correlation function (blue) for particles of PDEM$_{70}$-*co*-PDOM$_{30}$ **C.** DLS size distribution showing the hydrodynamic diameter as a function of scattered intensity (black) and the corresponding correlation function (blue) for particles of PDEM$_{100}$-*co*-PDOM$_{100}$ and **D.** DLS size distribution showing the hydrodynamic diameter as a function of particle number (black) and the corresponding correlation function (blue) for particles of PDEM$_{100}$-*co*-PDOM$_{100}$.

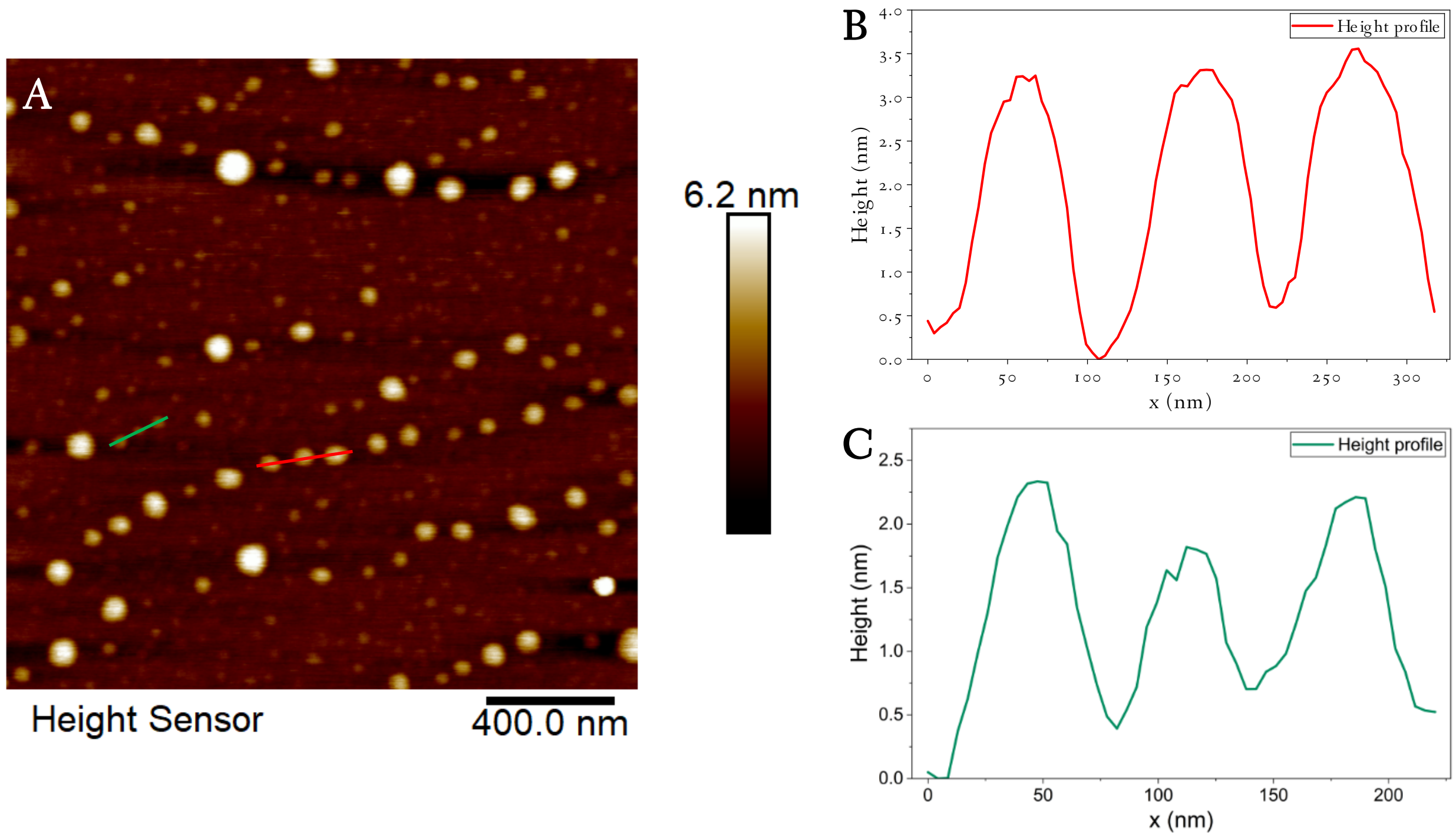

Figure S31 A. AFM height image of nanoparticles of PDEM$_{60}$-*co*-PDOM$_{40}$ deposited from isododecane and dried on a mica substrate. **B.** Height profile extracted along the red line shown in the AFM image. **C.** Height profile extracted along the green line shown in the AFM image.

Thermal depolymerization of PDEM-co-PDOM:

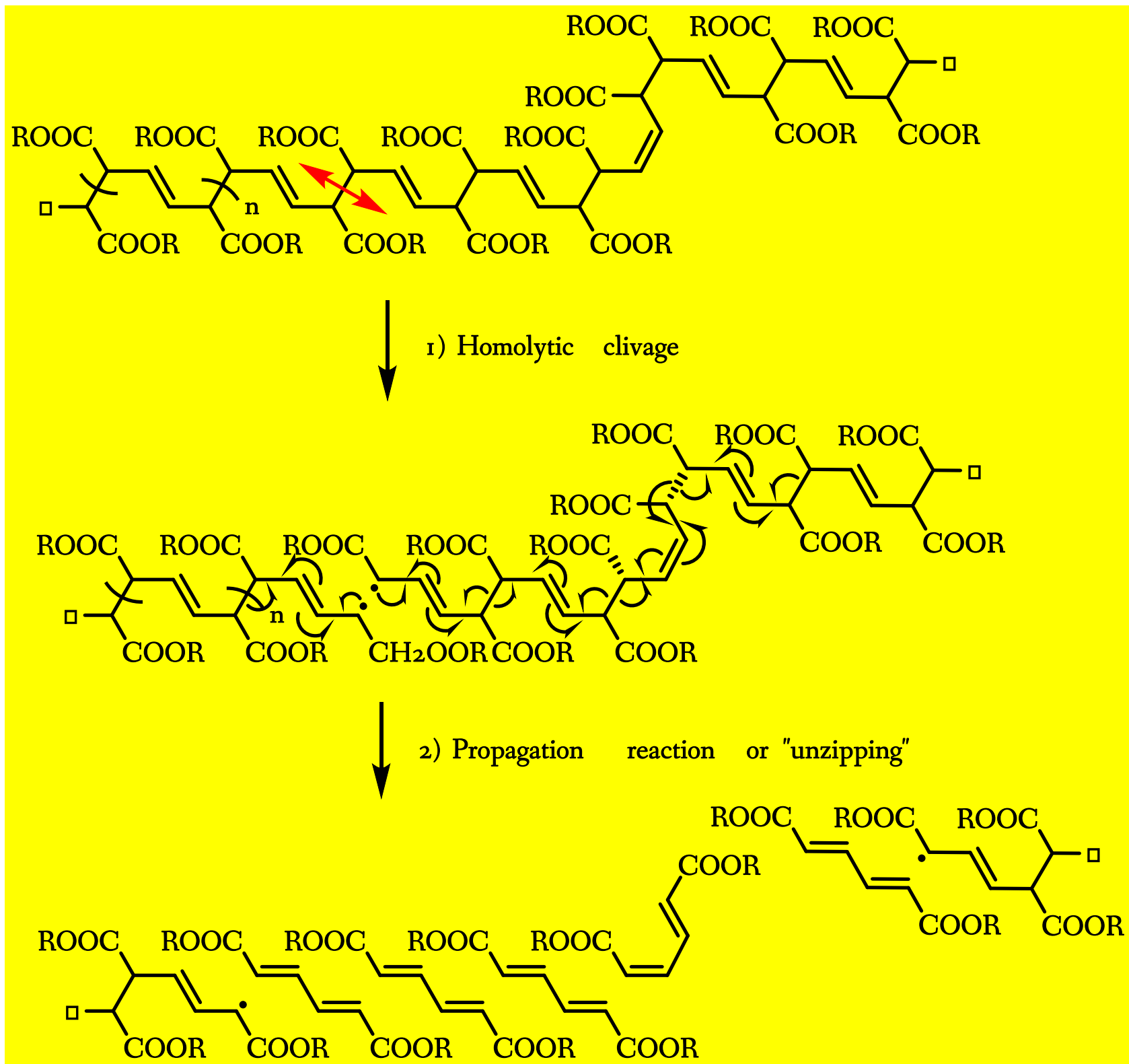

Scheme S4. Proposed mechanism for the thermal depolymerization of dialkyl muconate polymers. Thermal activation can induce homolytic cleavage of C-C bonds along the copolymer chains, forming radical species, which is followed by chain unzipping; this results in depolymerization and regeneration of the corresponding dialkyl muconate monomers.

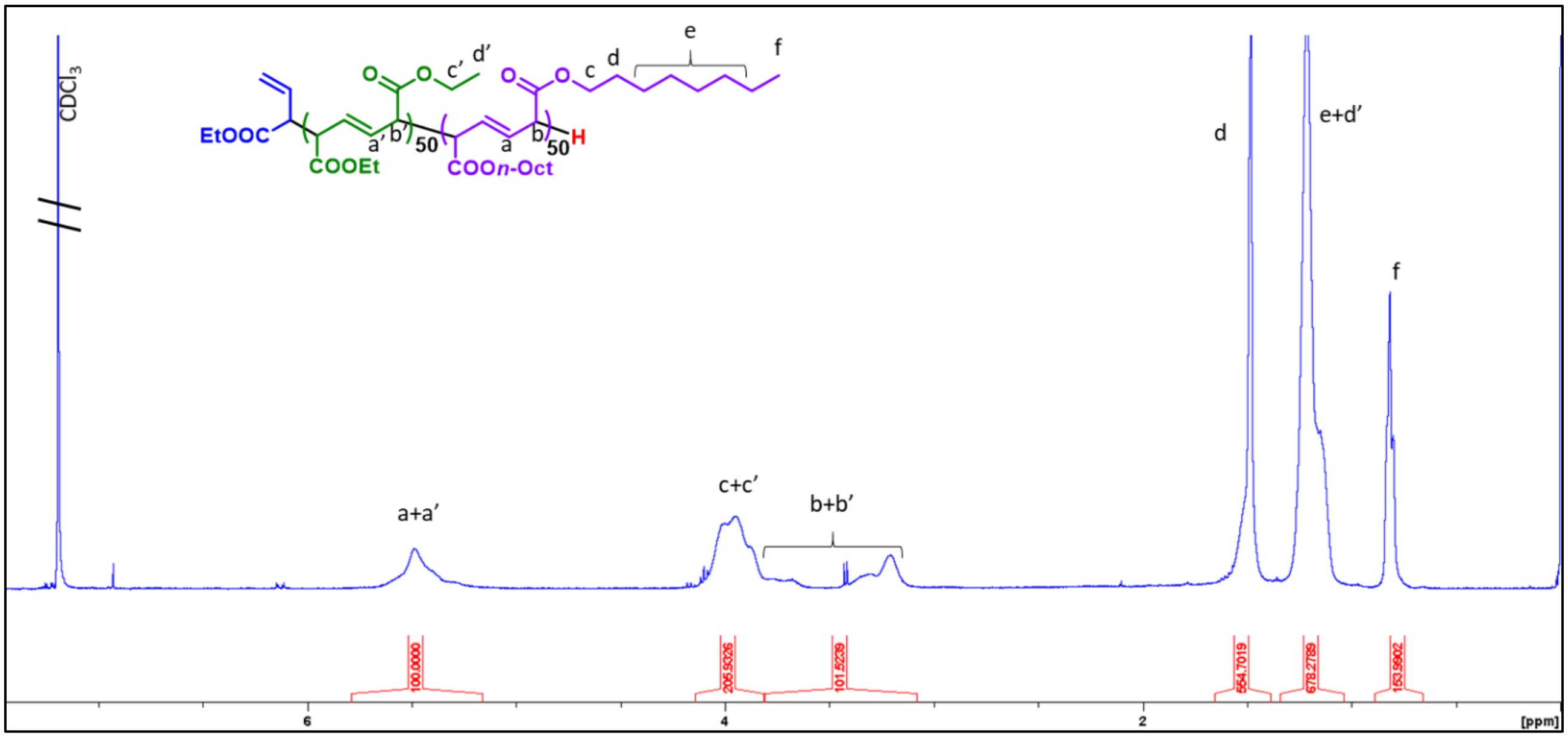

Figure S32. ^{1}H NMR of $PDEM_{50}$-*co*-$PDOM_{50}$ ($CDCl_3$, 400 MHz): δ 5.17-5.85 (m, 2H, RO_2-C-CH-C**H**=), 3.83-4.29 (m, 4H, R-C-O_2-C$\mathbf{H_2}$-); 3.13-3.83 (m, 2H, R-C-O_2-C**H**-CH=), 1.45-1.64 (m, 2H, R-C-O_2-CH_2-C$\mathbf{H_2}$-), 1.12-1.36 (m, (10H ; 3H), (-(C$\mathbf{H_2}$)$_5$- ; R-C-O_2-CH_2-C$\mathbf{H_3}$)), 0.82-0.92 (m, 3H, -$(CH_2)_7$-C$\mathbf{H_3}$).

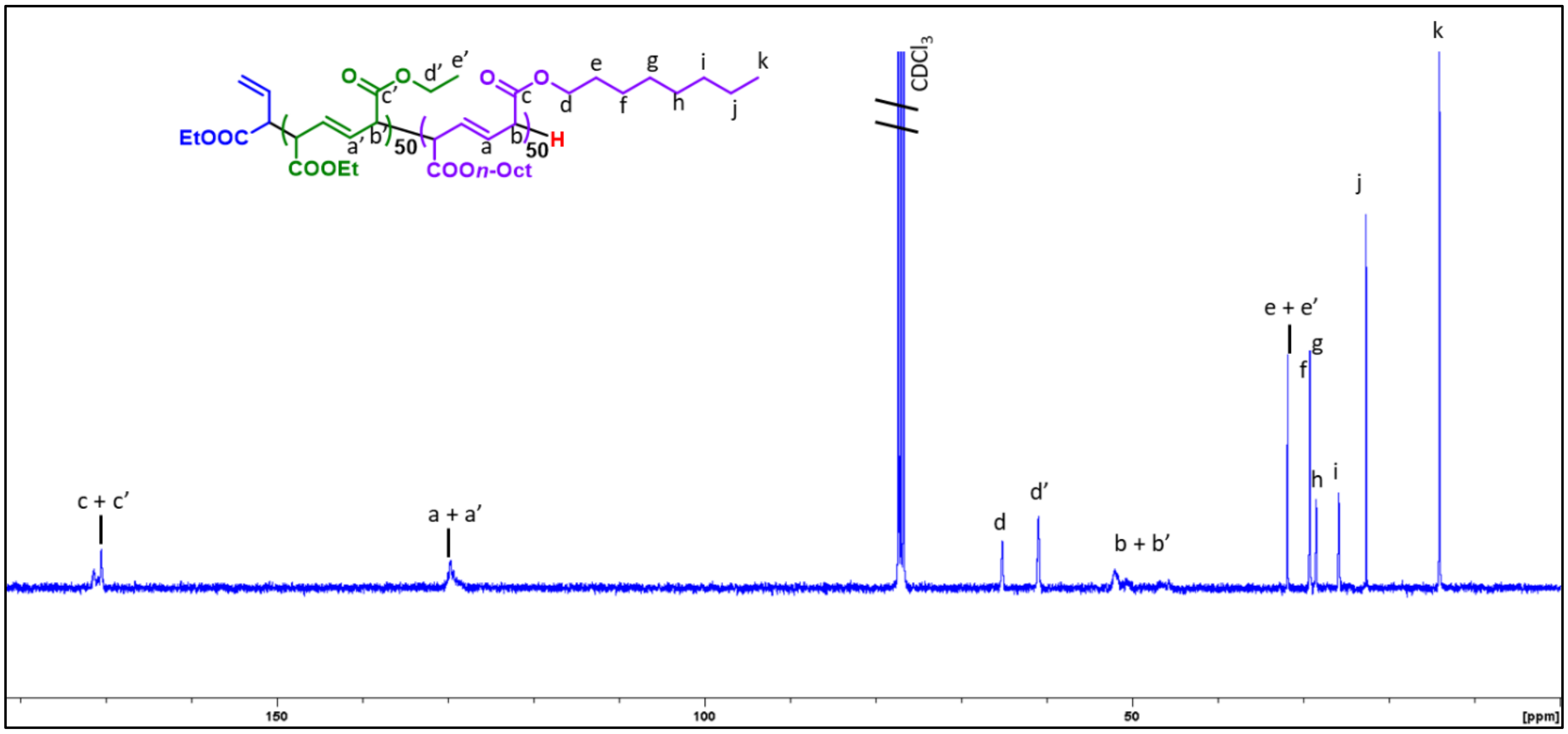

Figure S33. ^{13}C NMR of PDEM$_{50}$-*co*-PDOM$_{50}$ ($CDCl_3$, 400 MHz): PDEM: δ 170.20-171.90 (R-O_2-**C**-R), 127.81-130.45 (R-O_2-C-CH=**C**H), 60.62-61.32 (-**C**H_2-CH_3), 49.80-52.55 (R-O_2-C-**C**H=CH, trans), 45.00-47.60 (RO_2-C-**C**H=CH, cis), 14.04 (-CH_2-**C**H_3). PDOM: 128.00-131.60 (R-O_2-C-**C**H=CH), 65.23 (R-CO_2-**C**H_2-$(CH_2)_6$-CH_3), 49.99-53.15 (R-O_2-C-**C**H=CH, trans), 45.01-49.05 (R-O_2-C-**C**H=CH, cis), 31.86 (R-C-O_2-CH_2-**C**H_2-$(CH_2)_5$-CH_3), 29.30 (R-C-O_2-$(CH_2)_2$-**C**H_2-$(CH_2)_4$-CH_3), 29.26 (R-C-O_2- $(CH_2)_3$-**C**H_2-$(CH_2)_3$-CH_3), 28.52 (R-C-O_2-(CH2)$_4$-CH_2-$(CH_2)_2$-CH_3), 25.93 (R-C-O_2-$(CH_2)_5$-**C**H_2- CH_2-CH_3), 25.25 (R-C-O_2-$(CH_2)_6$-**C**H_2-CH_3), 22.66 (R-C-O_2-$(CH_2)_7$-**C**H_3).

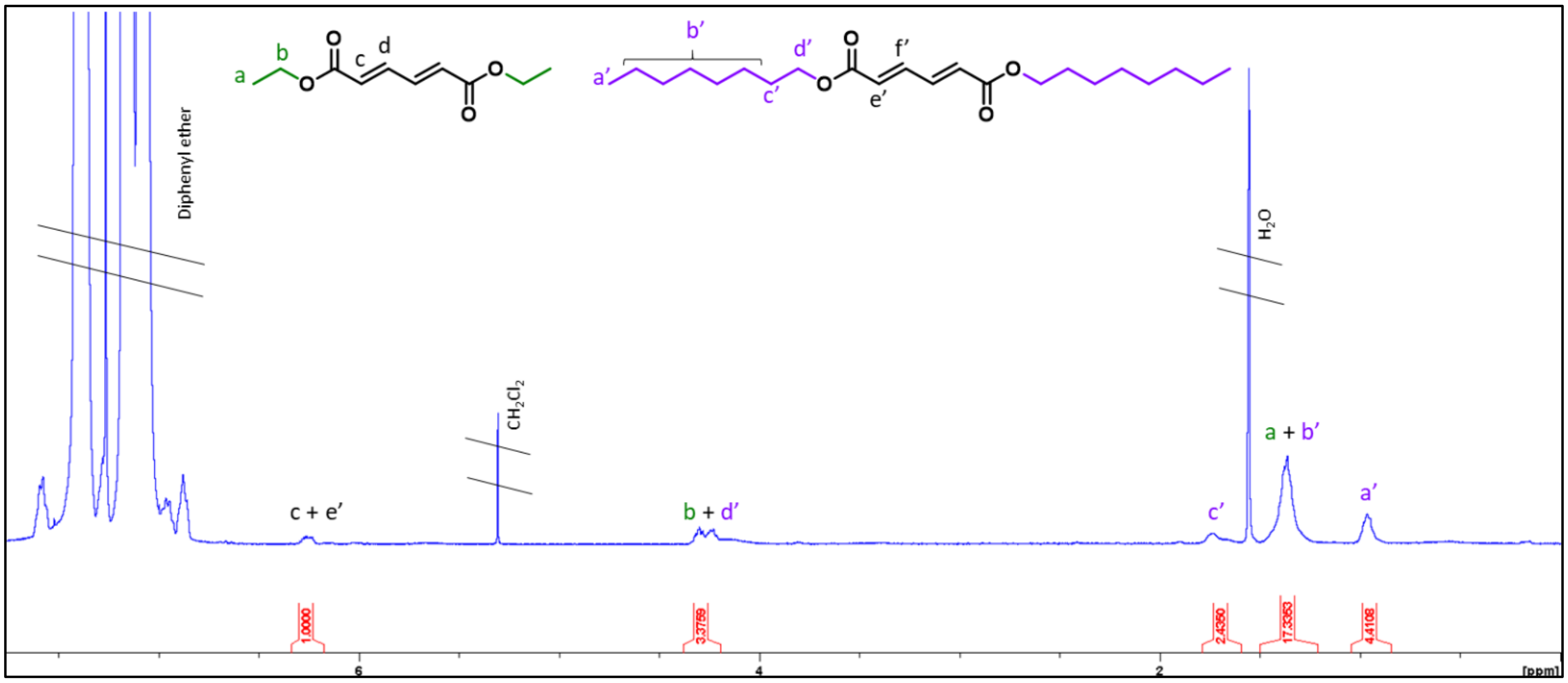

Figure S34. ^{1}H NMR of crude product (mixture of DEM and DOM monomers) obtained after depolymerization treatment of $PDEM_{50}$-*co*-$PDOM_{50}$ in diphenyl ether. ($CDCl_3$, 400 MHz): δ 5.17-5.85 (m, 2H, RO_2-C-CH-CH=), 3.83-4.29 (m, 4H, R-C-O_2-$\mathbf{CH_2}$-); 3.13-3.83 (m, 2H, R-C-O_2-**CH**-CH=), 1.45-1.64 (m, 2H, R-C-O_2-CH_2-$\mathbf{CH_2}$-), 1.12-1.36 (m, (10H ; 3H), (-$(\mathbf{CH_2})_5$- ; R-C-O_2-CH_2-$\mathbf{CH_3}$)), 0.82-0.92 (m, 3H, -$(CH_2)_7$-$\mathbf{CH_3}$).

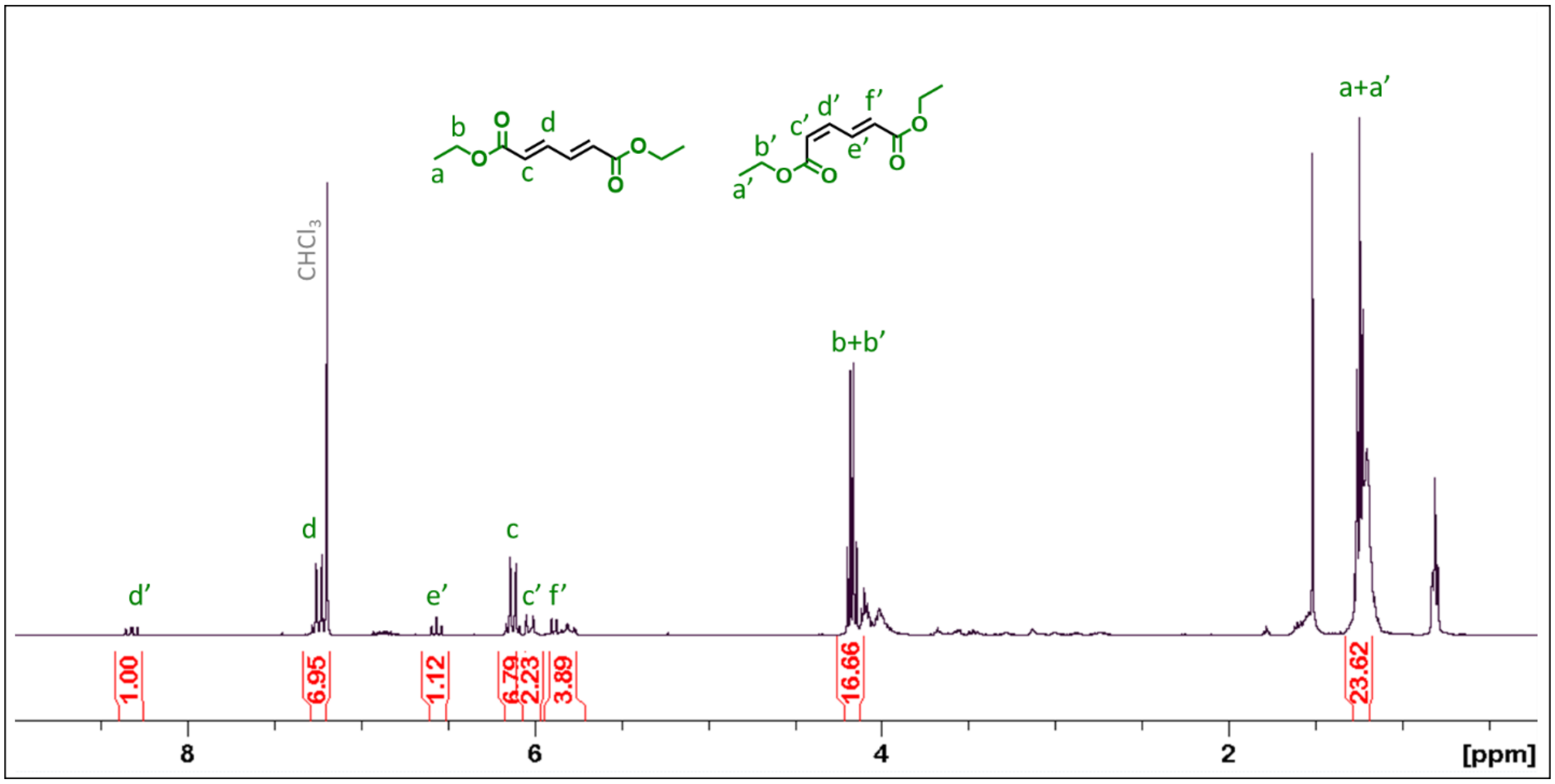

Figure S35. ^{1}H NMR spectrum of recycled E,E and Z,E-DEM monomer mixture (75% and 25%, respectively) obtained after thermal depolymerization (Yield: 75%).

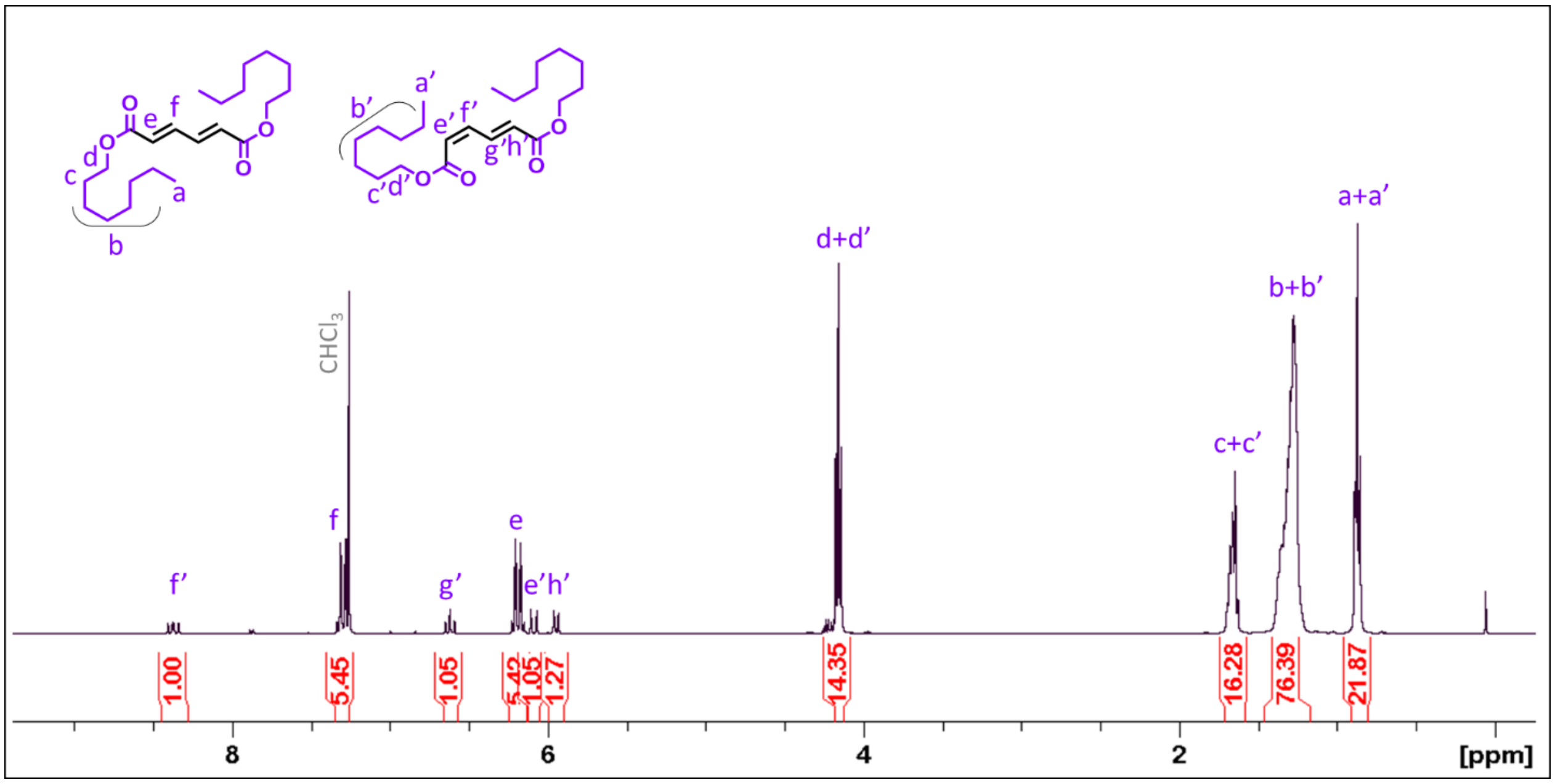

Figure S36. ^{1}H NMR spectrum of recycled E,E and Z,E-DOM monomer mixture (73% and 27%, respectively) obtained after thermal depolymerization.

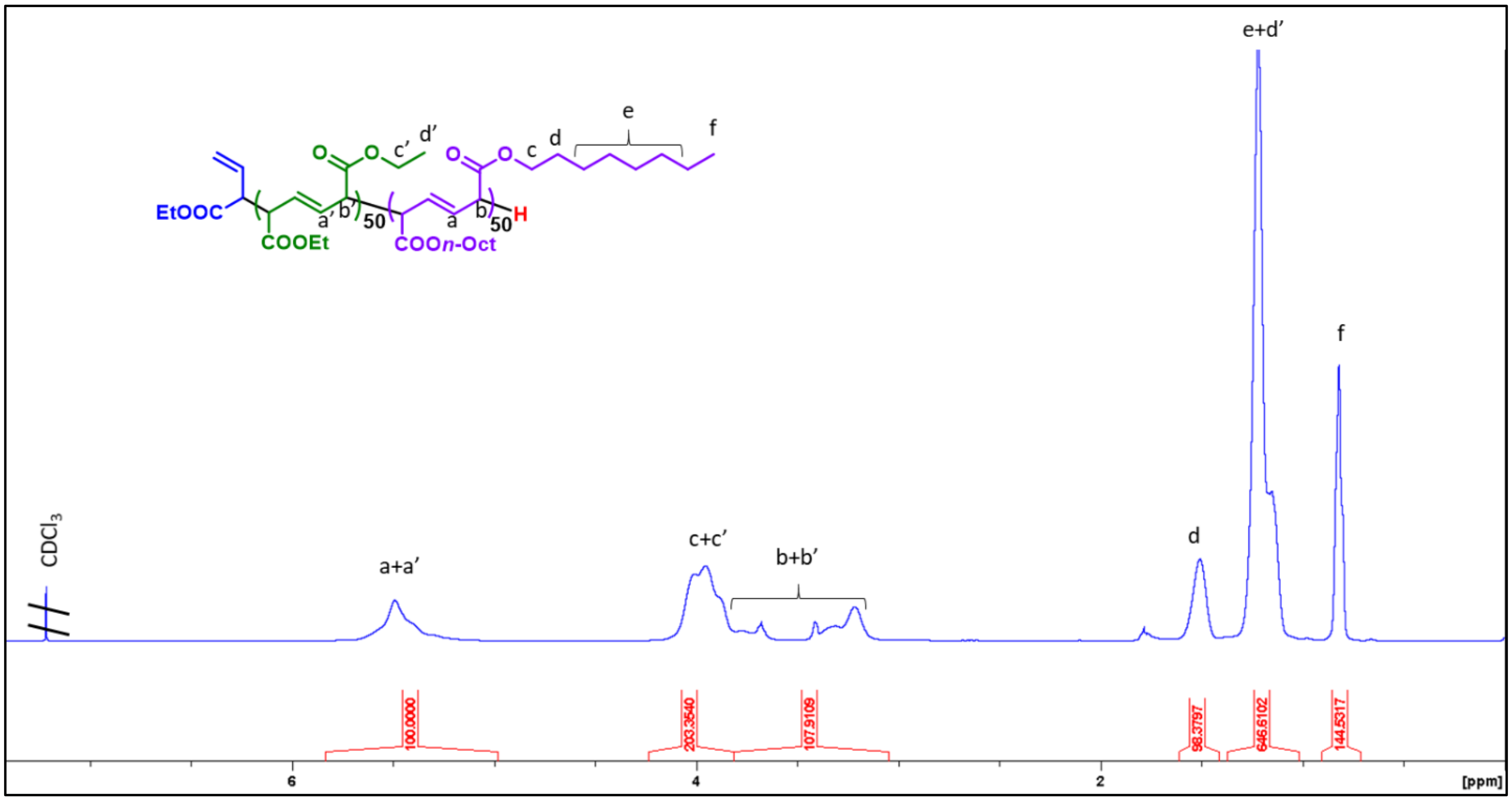

Figure S37. ^{1}H NMR of recycled PDEM$_{50}$-*co*-PDOM$_{50}$ ($CDCl_3$, 400 MHz): δ 5.17-5.85 (m, 2H, RO_2-C-CH-C**H**=), 3.83-4.29 (m, 4H, R-C-O_2-C$\mathbf{H_2}$-); 3.13-3.83 (m, 2H, R-C-O_2-C**H**-CH=), 1.45-1.64 (m, 2H, R-C-O_2-CH_2-C$\mathbf{H_2}$-), 1.12-1.36 (m, (10H ; 3H), (-(C$\mathbf{H_2}$)$_5$- ; R-C-O_2-CH_2-C$\mathbf{H_3}$)), 0.82-0.92 (m, 3H, -$(CH_2)_7$-C$\mathbf{H_3}$).

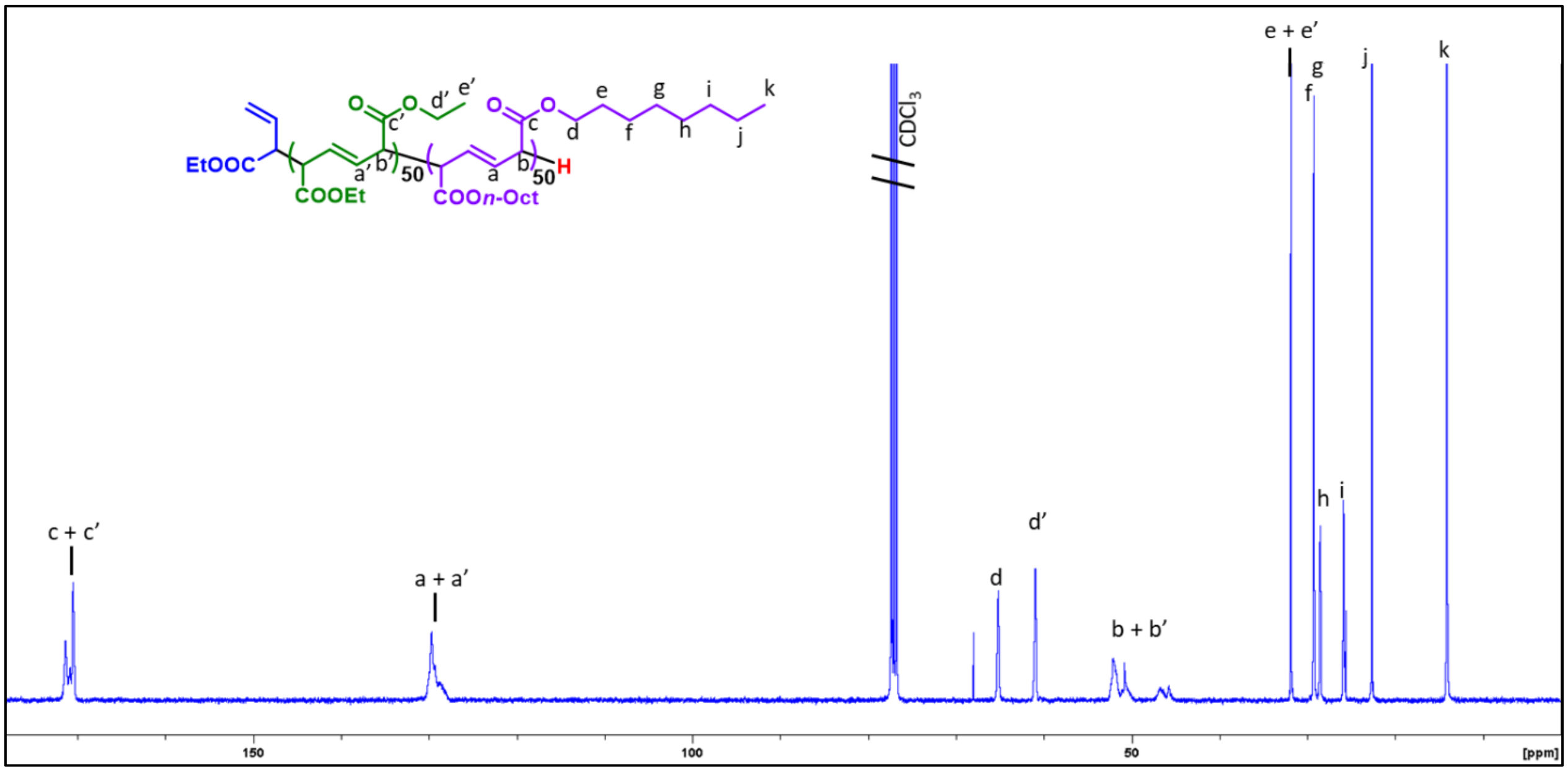

Figure S38. ^{13}C NMR of recycled $PDEM_{50}$-*co*-$PDOM_{50}$ ($CDCl_3$, 400 MHz): PDEM: δ 170.20-171.90 (R-O_2-**C**-R), 127.81-130.45 (R-O_2-C-CH=**C**H), 60.62-61.32 (-**C**H_2-CH_3), 49.80-52.55 (R-O_2-C-**C**H=CH, trans), 45.00-47.60 (RO_2-C-**C**H=CH, cis), 14.04 (-CH_2-**C**H_3). PDOM: 128.00-131.60 (R-O_2-C-**C**H=CH), 65.23 (R-CO_2-**C**H_2-$(CH_2)_6$-CH_3), 49.99-53.15 (R-O_2-C-**C**H=CH, trans), 45.01-49.05 (R-O_2-C-**C**H=CH, cis), 31.86 (R-C-O_2-CH_2-**C**H_2-$(CH_2)_5$-CH_3), 29.30 (R-C-O_2-$(CH_2)_2$-**C**H_2-$(CH_2)_4$-CH_3), 29.26 (R-C-O_2- $(CH_2)_3$-**C**H_2-$(CH_2)_3$-CH_3), 28.52 (R-C-O_2-(CH2)$_4$-CH_2-$(CH_2)_2$-CH_3), 25.93 (R-C-O_2-$(CH_2)_5$-**C**H_2- CH_2-CH_3), 25.25 (R-C-O_2-$(CH_2)_6$-**C**H_2-CH_3), 22.66 (R-C-O_2-$(CH_2)_7$-**C**H_3).

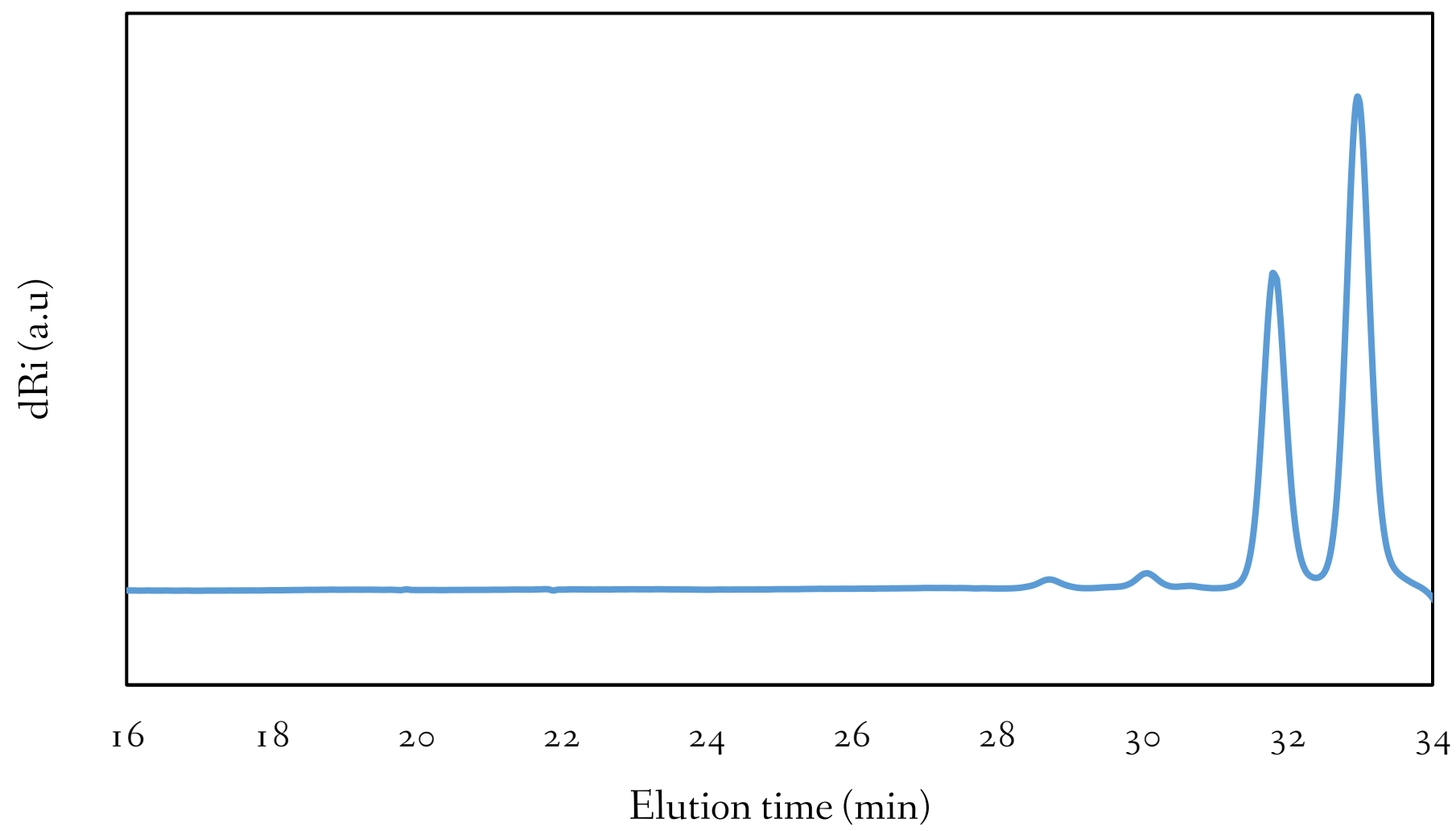

Figure S39. SEC trace of depolymerization crude mixture after thermal treatment, only low molecular weight compounds remain (time > 28 min). The peak at 33 min corresponds to the mix of DEM and DPE and the peak at 31.5 min corresponds to the DOM monomer.

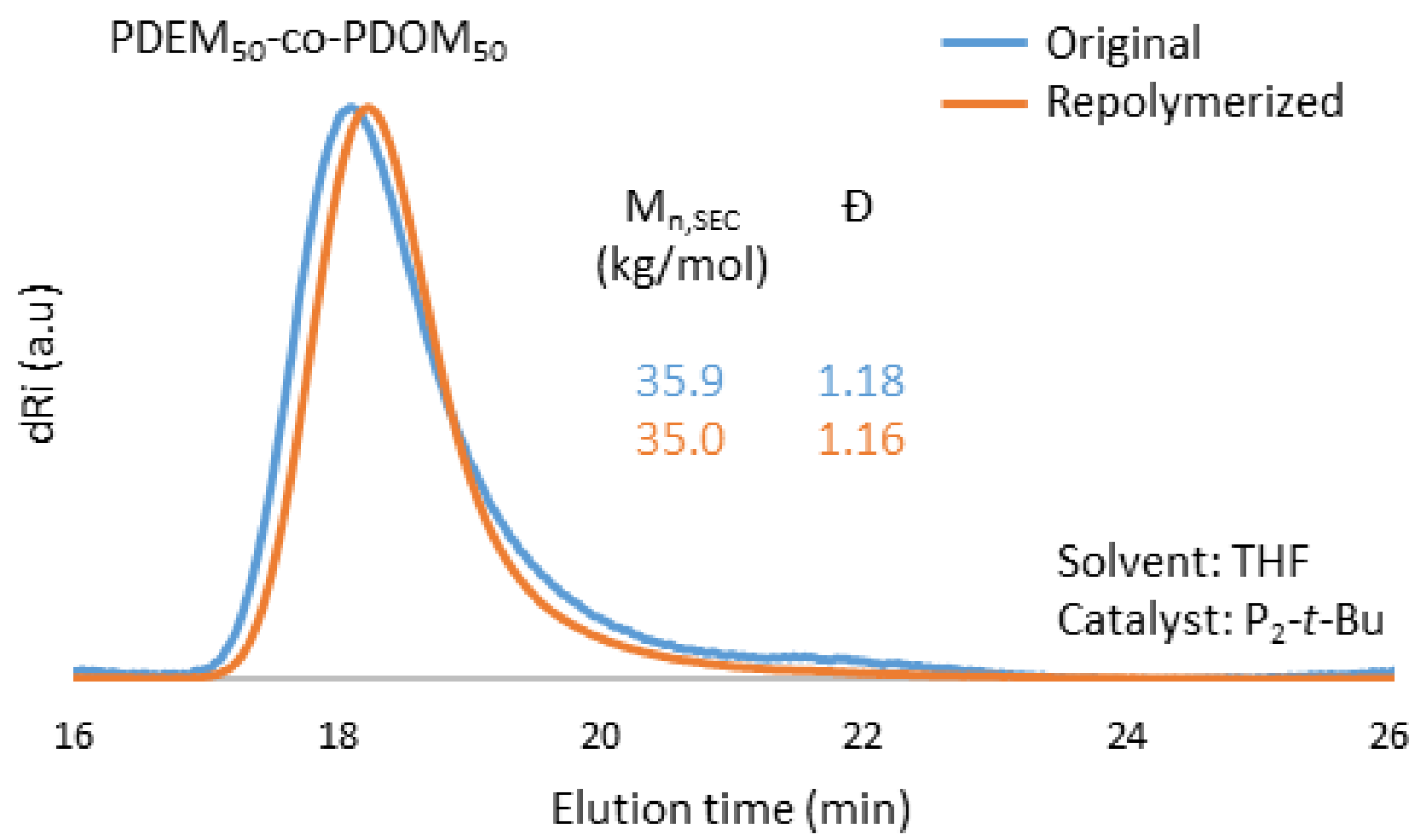

Figure S40. SEC traces of copolymers of DEM and DOM before (blue) and after depolymerization and repolymerization (orange) in THF using P_2-*t*-Bu as catalyst, showing the retention of low dispersity and similar molar masses.

^{1}H NMR of dialkyl muconates:

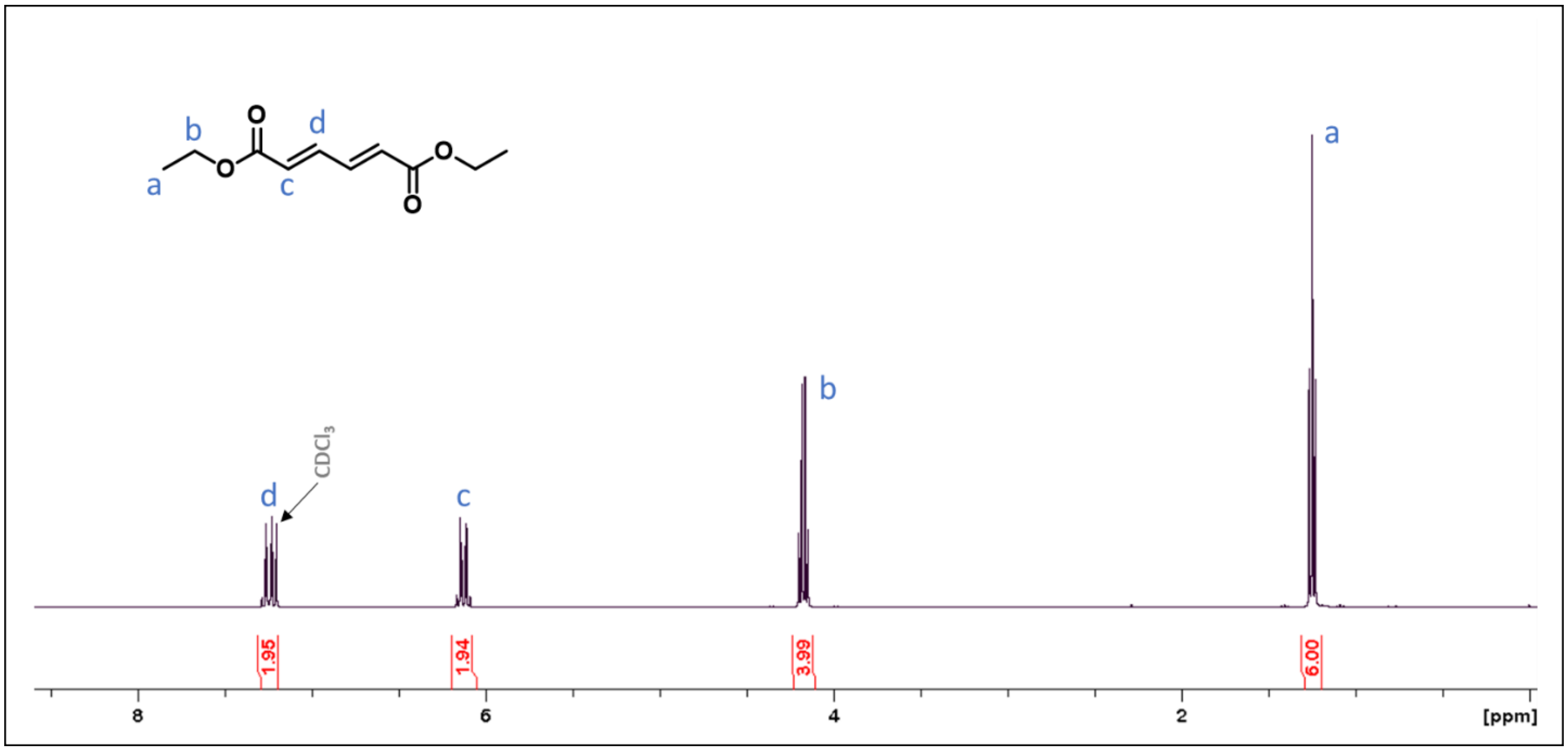

Figure S41. ^{1}H NMR of E,E-DEM ($CDCl_3$, 400 MHz): δ 7.30 (q, 2H, R-O_2-C-CH=CH-), 6.19 (m, 2H, R-O_2-C-CH=CH-), 4.23 (q, 4H, -CH_2-), 1.31 (t, 6H, -CH_2-CH_3).

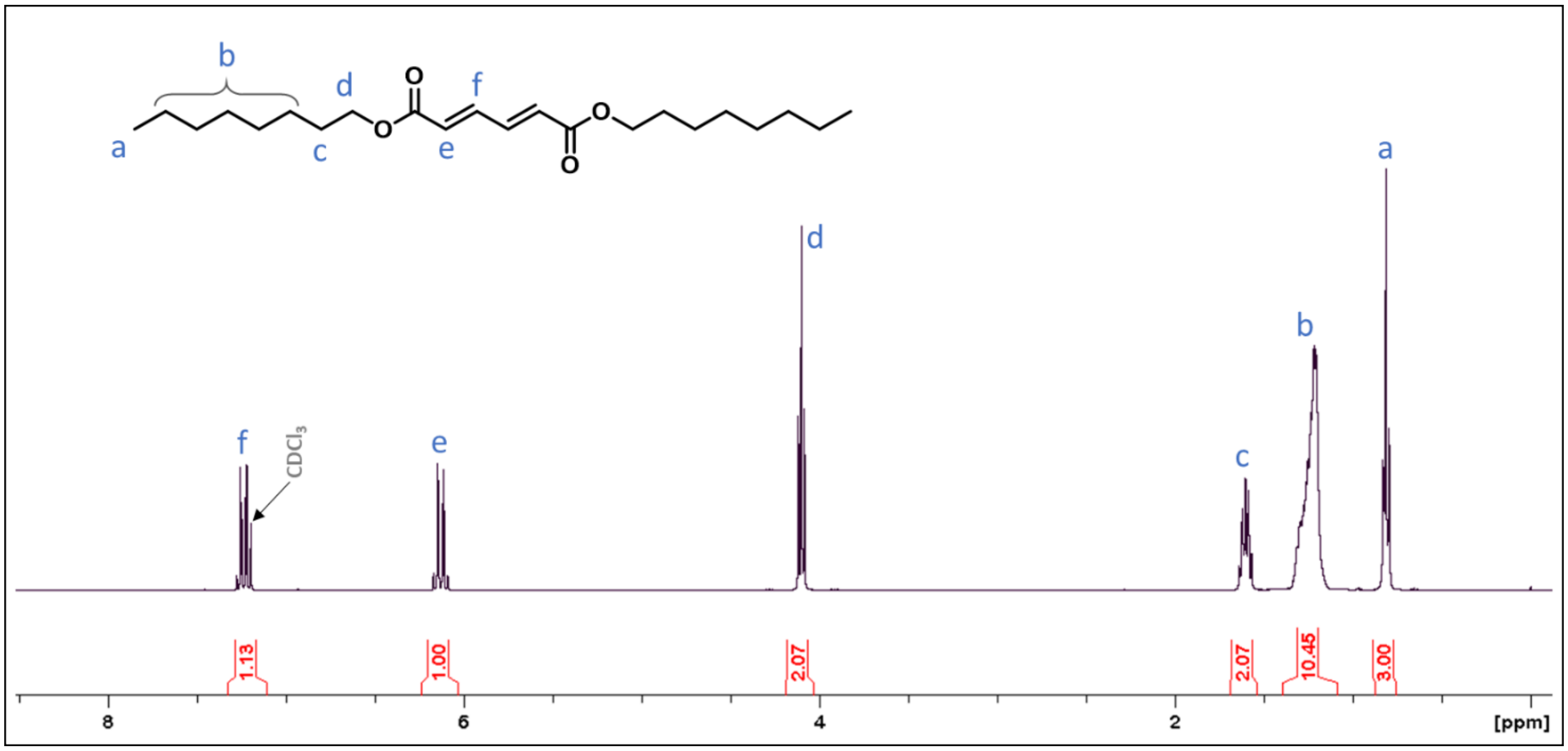

Figure S42. ^{1}H NMR of E,E-DOM ($CDCl_3$, 400 MHz): δ 7.29 (m, 2H, R-O_2-C-CH=C**H**-), 6.19 (m, 2H, R-O_2-C-C**H**=CH-), 4.16 (q, 4H, -R-C-O_2-C**H$_2$**-), 1.60-1.72 (m, 4H, - R-C-O_2-CH_2-C**H$_2$**-), 1.19-1.41 (m, 20H, -(C**H$_2$**)$_5$-), 0.88 (t, 6H, -CH_2-C**H$_3$**)

^{13}C NMR of dialkyl muconates:

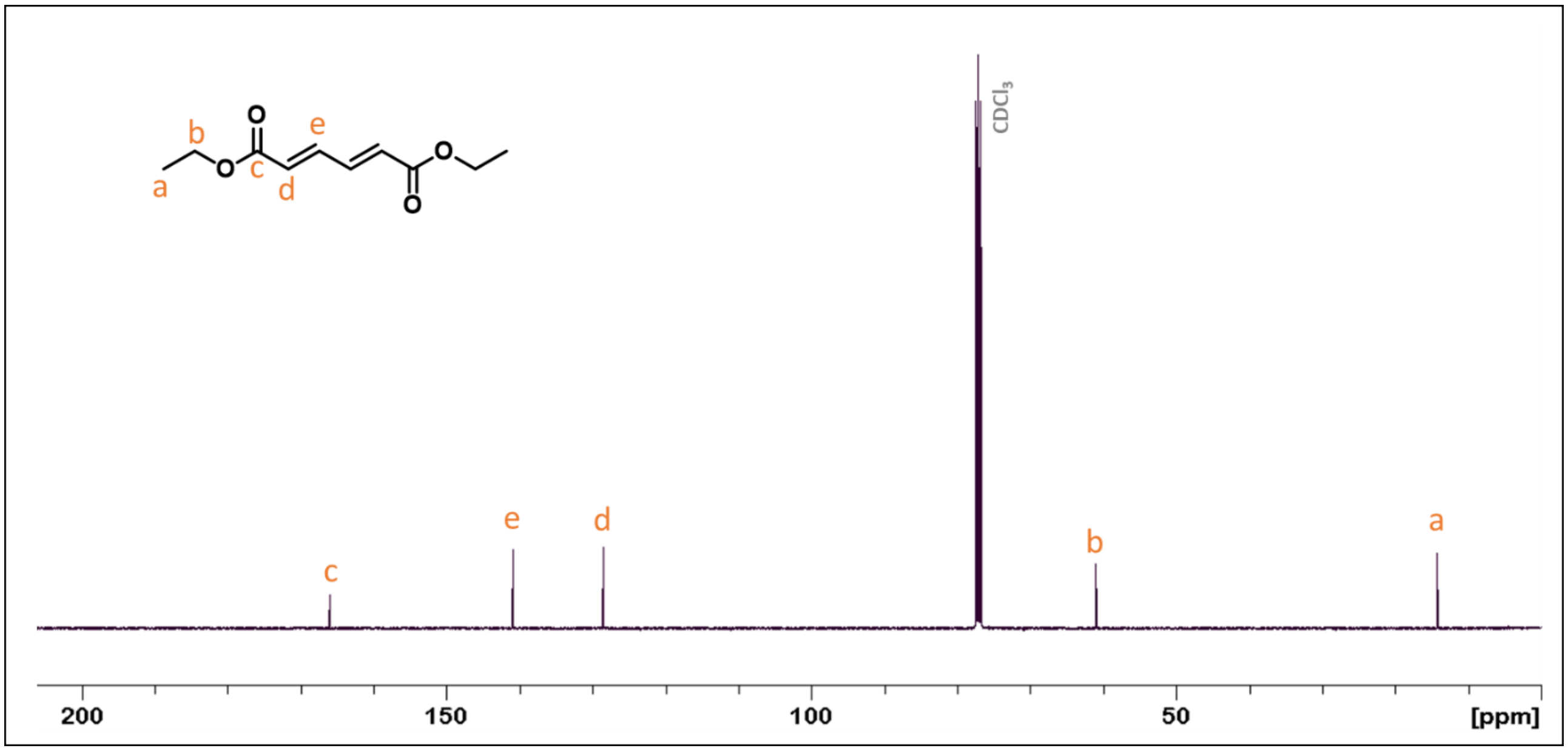

Figure S43. ^{13}C NMR of E,E-DEM ($CDCl_3$, 400 MHz): δ 165.93 (RO_2-**C**-R), 140.79 (R-O_2-C-**C**H=CH), 128.40 (R-O_2-C-CH=**C**H), 60.87 (-**C**H_2-CH_3), 14.21(-CH_2-**C**H_3).

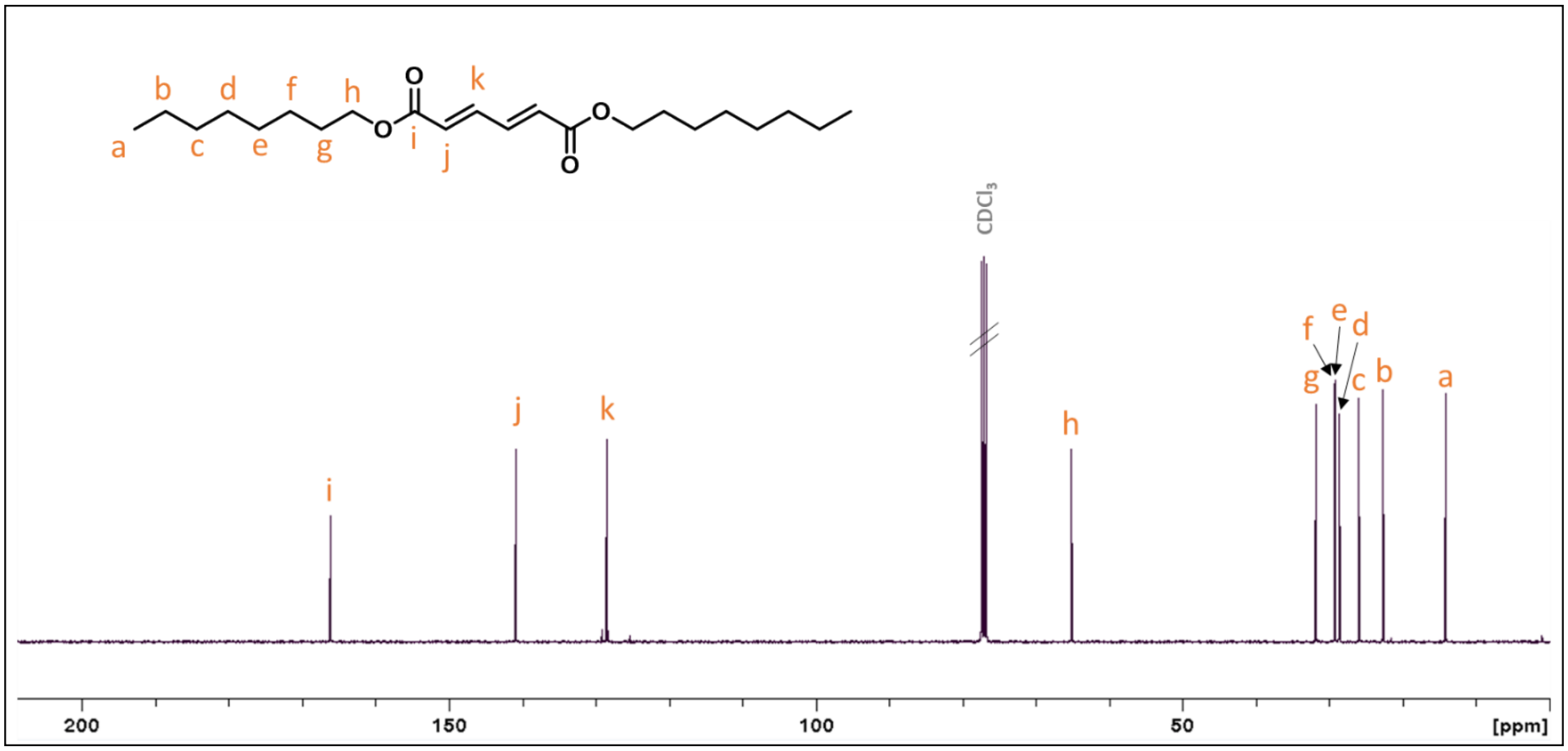

Figure S44. ^{13}C NMR of E,E-DOM ($CDCl_3$, 400 MHz): δ 165.99 (R-O_2-C-R), 140.75 (R-O_2-C-CH=CH), 128.40 (R-O_2-C-CH=CH), 65.07 (R-C-O_2-CH_2-$(CH_2)_6$-CH_3), 31.77 (R-C-O_2-CH_2-CH_2-$(CH_2)_5$-CH_3), 29.20 (R-C-O_2-$(CH_2)_2$-CH_2-$(CH_2)_4$-CH_3), 29.16 (R-C-O_2- $(CH_2)_3$-CH_2-$(CH_2)_3$-CH_3), 28.60 (R-C-O_2-$(CH_2)_4$-CH_2-$(CH_2)_2$-CH_3), 25.91 (R-C-O_2-$(CH_2)_5$-CH_2-CH_2- CH_3), 22.62 (R-C-O_2-$(CH_2)_6$-CH_2-CH_3), 14.07 (R-C-O_2-$(CH_2)_7$-CH_3).

MALS analysis and dn/dc determination:

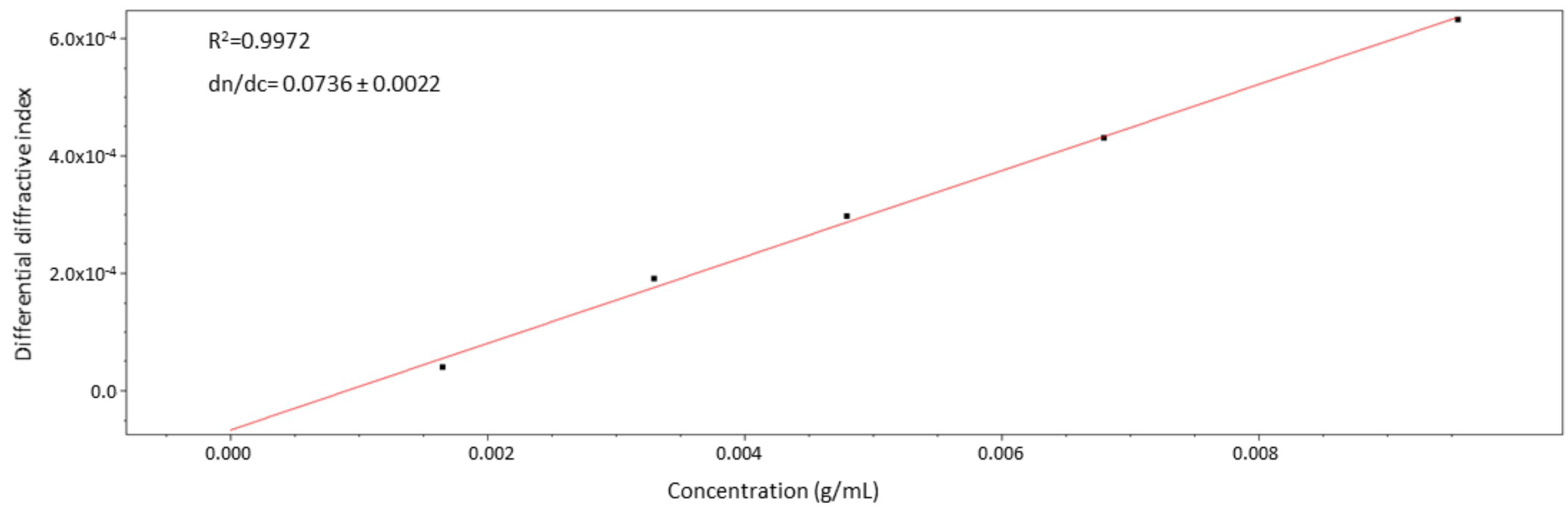

Figure S45. Refractive index curve *vs* $PDEM_{20}$-co-$PDOM_{80}$ concentration in THF at 40 °C.

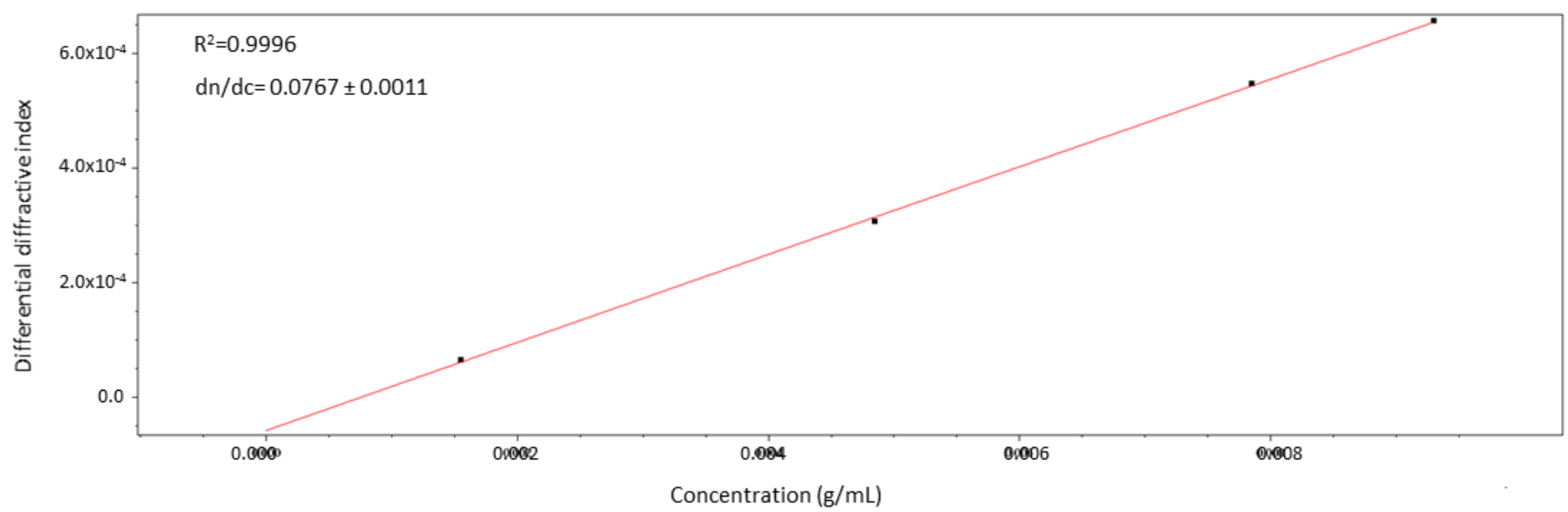

Figure S46. Refractive index curves $PDEM_{40}$-co-$PDOM_{60}$ concentration in THF at 40 °C.

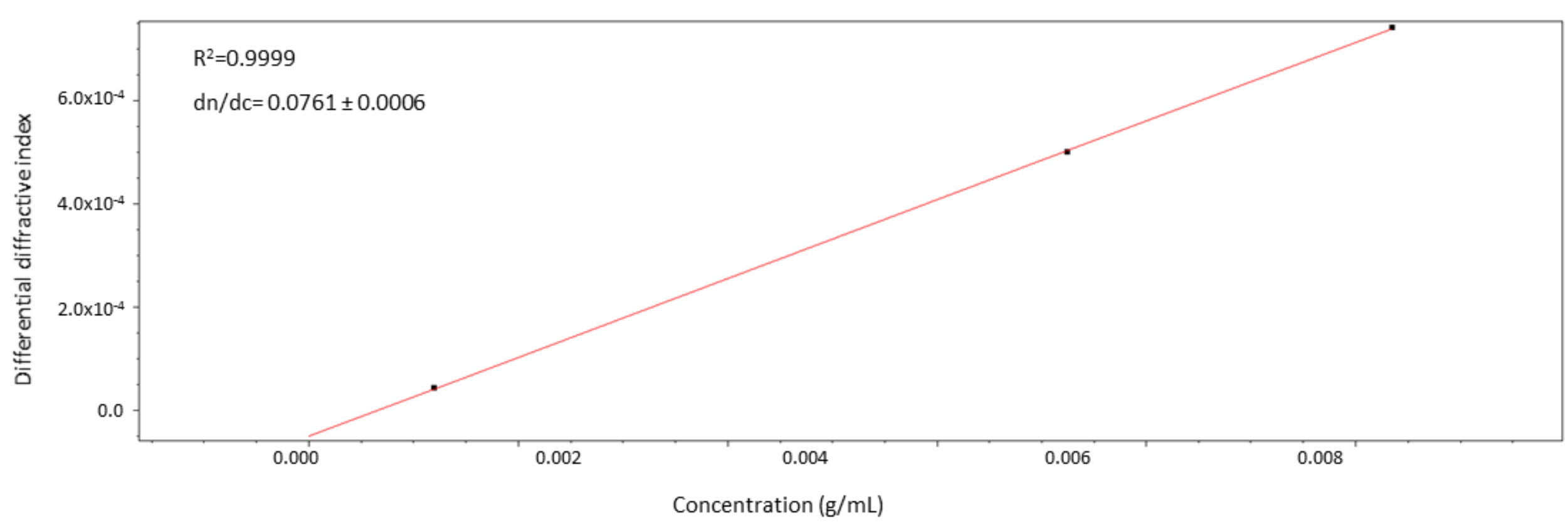

Figure S47. Refractive index curve *vs* $PDEM_{60}$-co-$PDOM_{40}$ concentration in THF at 40 °C.

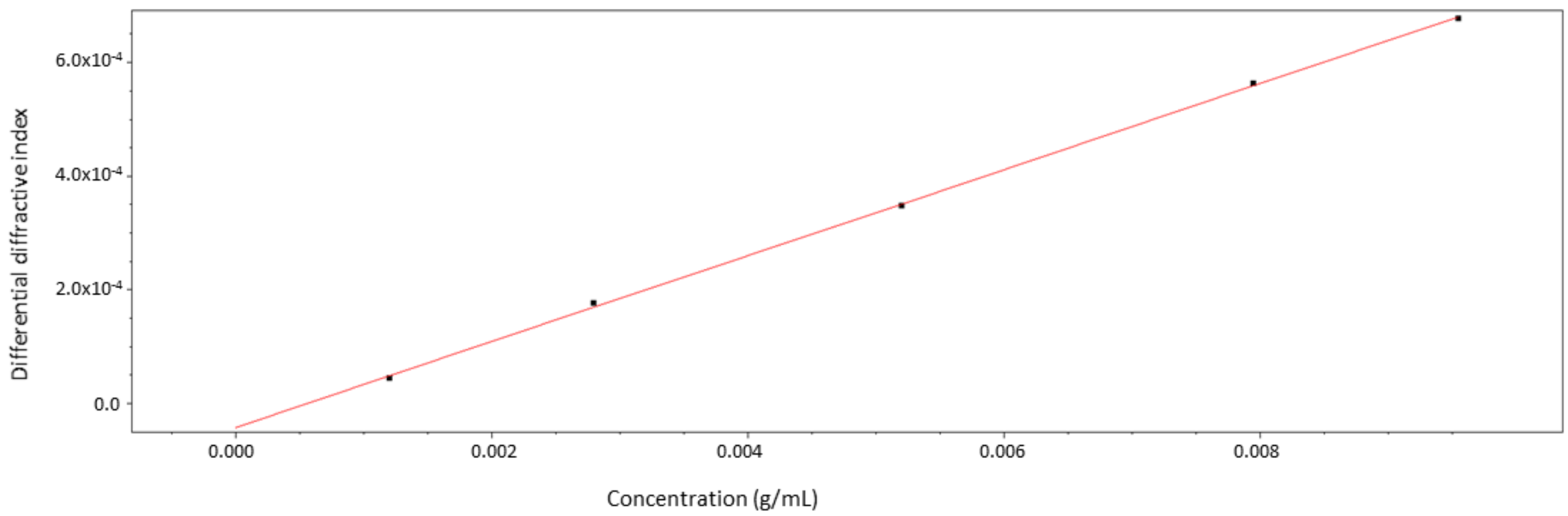

Figure S48. Refractive index curve *vs* $PDEM_{80}$-co-$PDOM_{20}$ concentration in THF at 40 °C.

Table S2. dn/dc values for the copolymers of DEM and DOM at different compositions, in THF at 40 °C.

Copolymer	dn/dc (mL/g)
$PDEM_{20}$-co-$PDOM_{80}$	0.0746 ± 0.0021
$PDEM_{40}$-co-$PDOM_{60}$	0.0767 ± 0.0011
$PDEM_{60}$-co-$PDOM_{40}$	0.0761 ± 0.0006
$PDEM_{80}$-co-$PDOM_{20}$	0.0757 ± 0.0008

^{1}H NMR of copoly(dialkyl muconate)s:

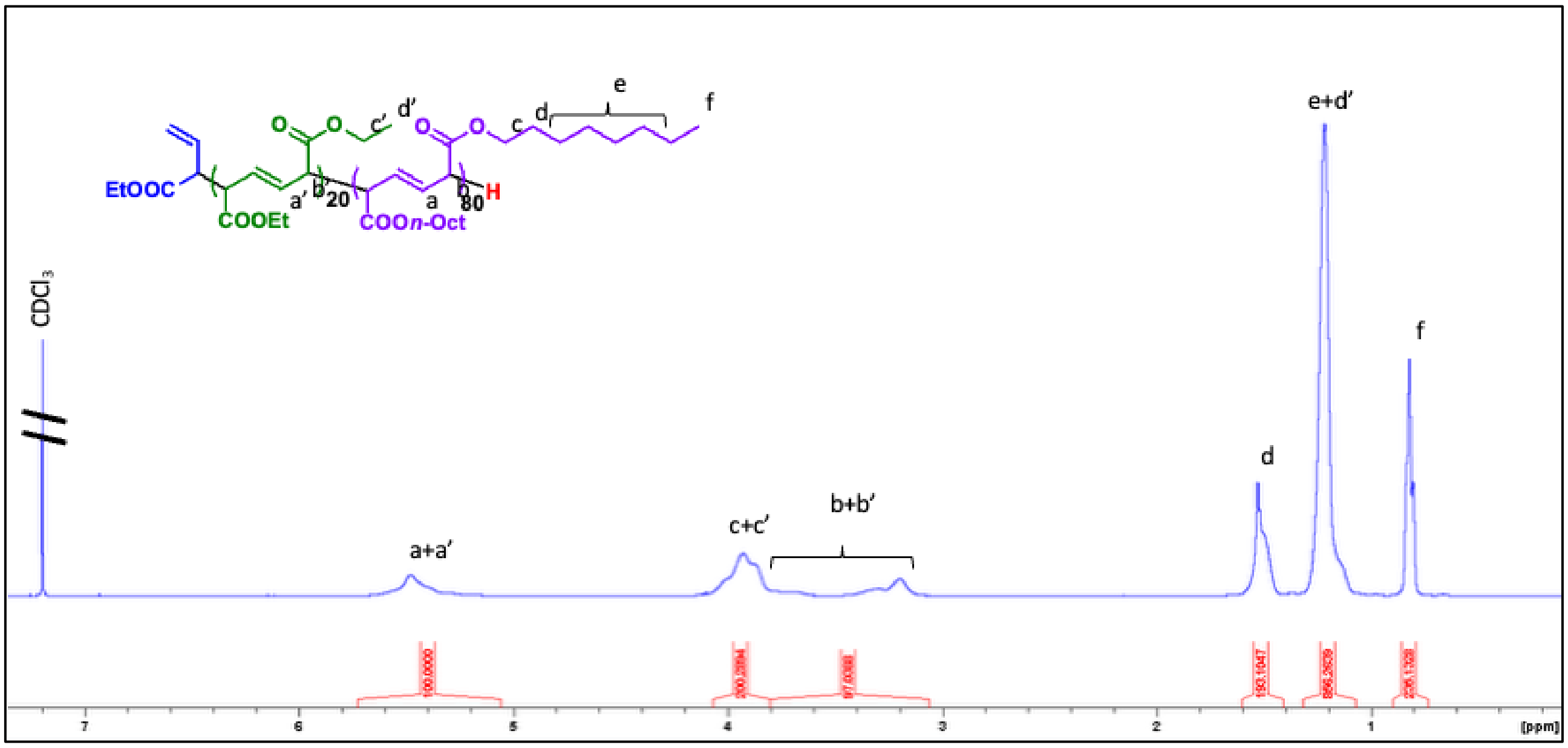

Figure S49. ^{1}H NMR of PDEM$_{20}$-*co*-PDOM$_{80}$ ($CDCl_3$, 400 MHz): δ 5.17-5.85 (m, 2H, RO_2-C-CH-C**H**=), 3.83-4.29 (m, 4H, R-C-O_2-C**H$_2$**-); 3.13-3.83 (m, 2H, R-C-O_2-C**H**-CH=), 1.45-1.64 (m, 2H, R-C-O_2-CH_2-C**H$_2$**-), 1.12-1.36 (m, (10H ; 3H), (-(C**H$_2$**)$_5$- ; R-C-O_2-CH_2-C**H$_3$**)), 0.82-0.92 (m, 3H, -(CH_2)$_7$-C**H$_3$**).

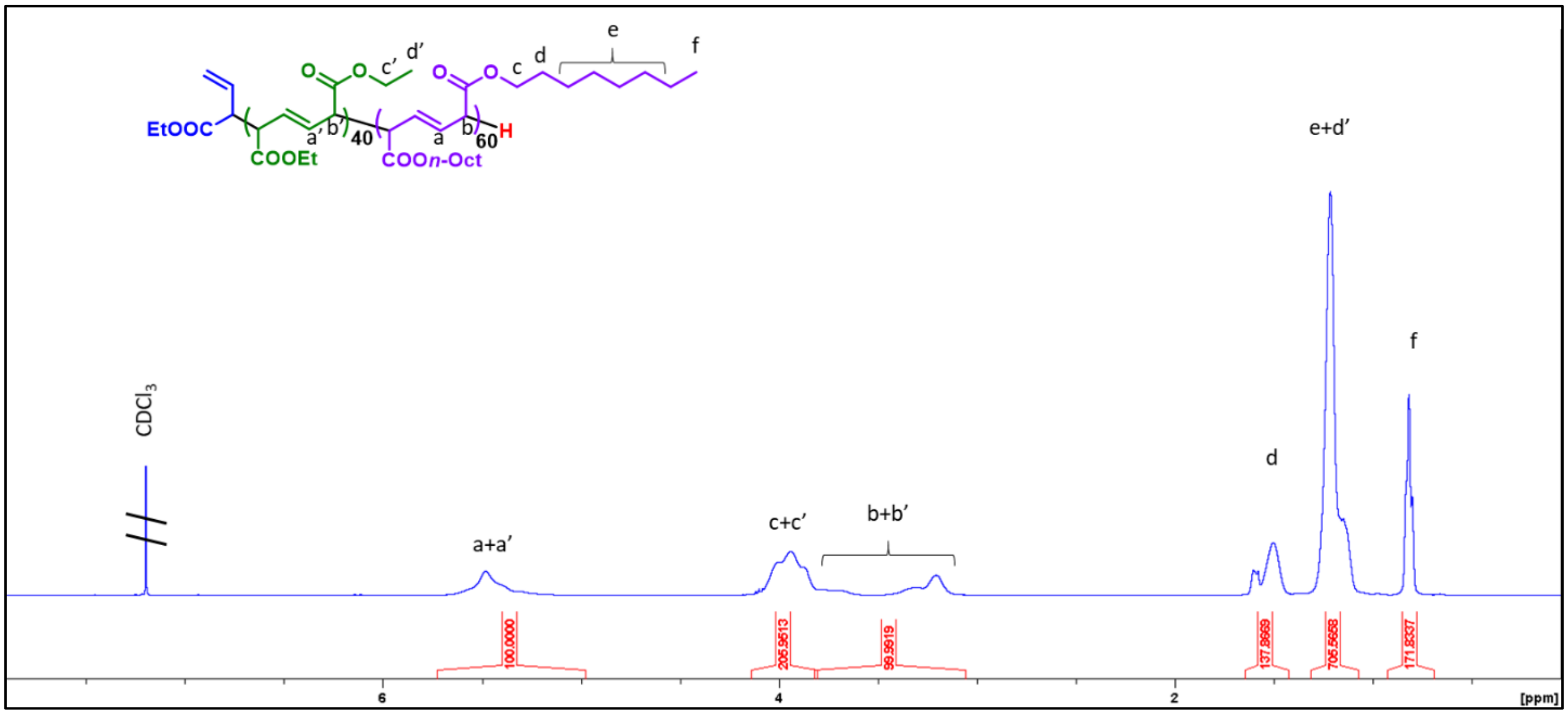

Figure S50. ^{1}H NMR of PDEM$_{40}$-*co*-PDOM$_{60}$ ($CDCl_3$, 400 MHz): δ 5.17-5.85 (m, 2H, RO_2-C-CH-C**H**=), 3.83-4.29 (m, 4H, R-C-O_2-C**H_2**-); 3.13-3.83 (m, 2H, R-C-O_2-C**H**-CH=), 1.45-1.64 (m, 2H, R-C-O_2-CH_2-C**H_2**-), 1.12-1.36 (m, (10H ; 3H), (-(C**H_2**)$_5$- ; R-C-O_2-CH_2-C**H_3**)), 0.82-0.92 (m, 3H, -$(CH_2)_7$-C**H_3**).

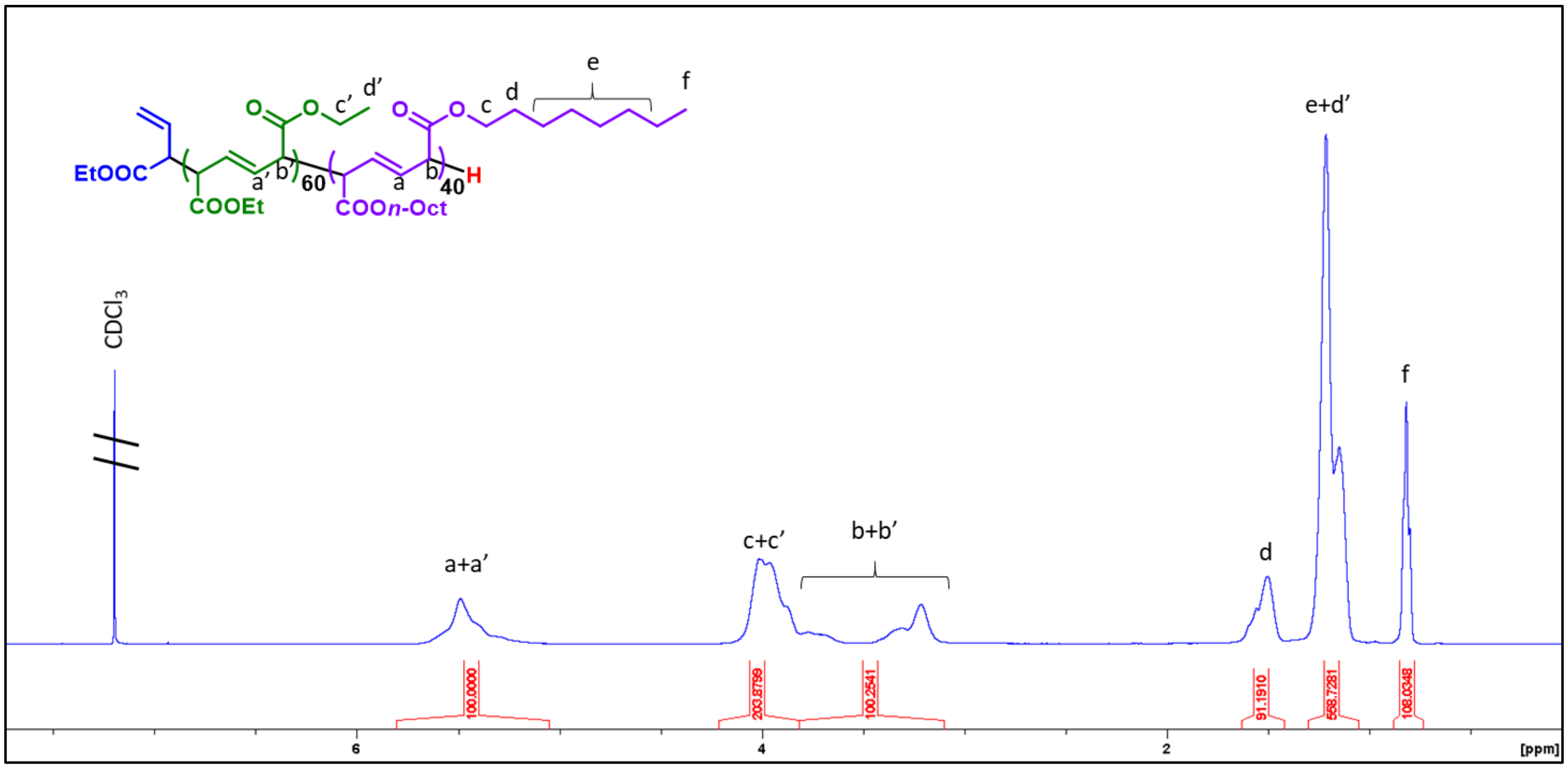

Figure S51. ^{1}H NMR of PDEM$_{60}$-*co*-PDOM$_{40}$ (CDCl$_3$, 400 MHz): δ 5.17-5.85 (m, 2H, RO$_2$-C-CH-C**H**=), 3.83-4.29 (m, 4H, R-C-O$_2$-C**H$_2$**-); 3.13-3.83 (m, 2H, R-C-O$_2$-C**H**-CH=), 1.45-1.64 (m, 2H, R-C-O$_2$-CH$_2$-C**H$_2$**-), 1.12-1.36 (m, (10H ; 3H), (-(C**H$_2$**)$_5$- ; R-C-O$_2$-CH$_2$-C**H$_3$**)), 0.82-0.92 (m, 3H, -(CH$_2$)$_7$-C**H$_3$**).

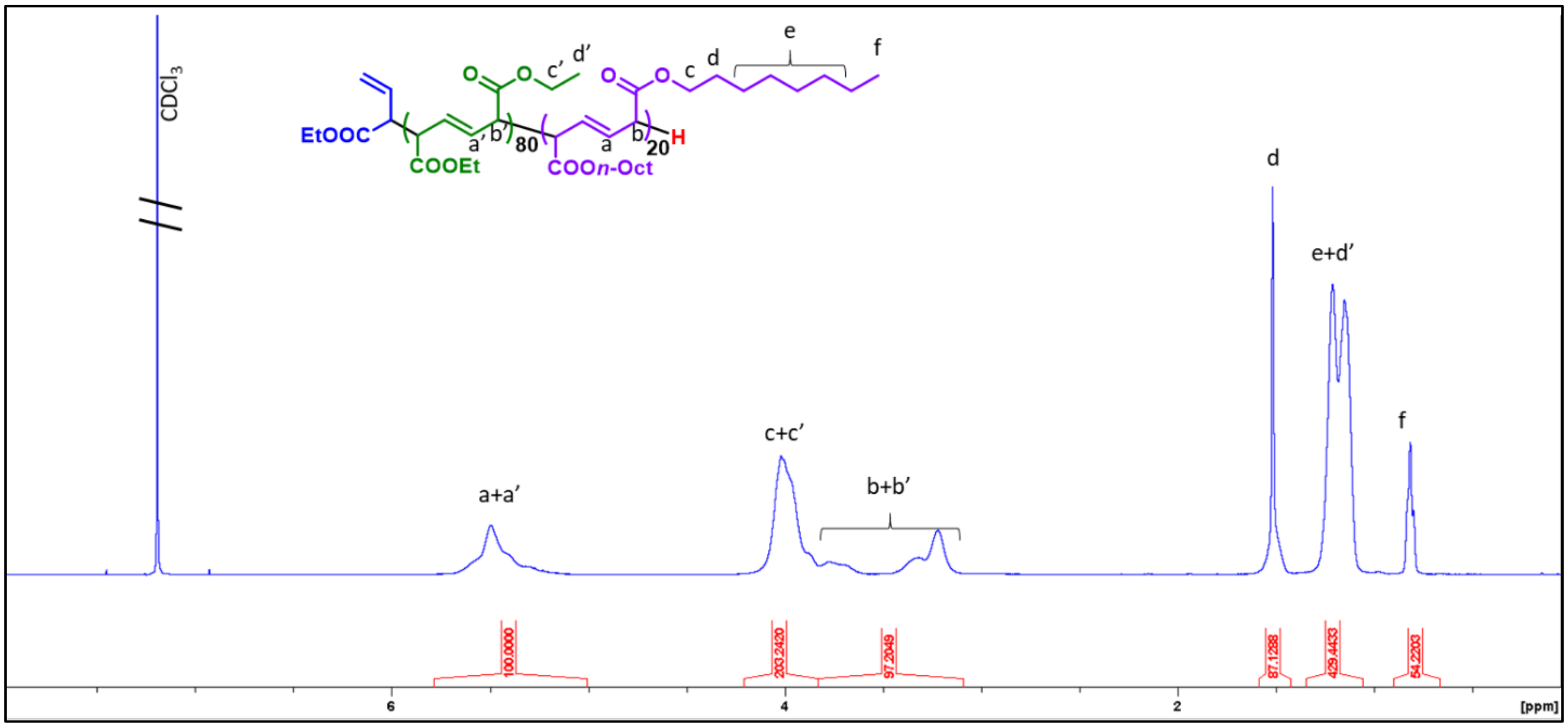

Figure S52. ^{1}H NMR of PDEM$_{80}$-*co*-PDOM$_{20}$ ($CDCl_3$, 400 MHz): δ 5.17-5.85 (m, 2H, RO_2-C-CH-C**H**=), 3.83-4.29 (m, 4H, R-C-O_2-C$\mathbf{H_2}$-); 3.13-3.83 (m, 2H, R-C-O_2-C**H**-CH=), 1.45-1.64 (m, 2H, R-C-O_2-CH_2-C$\mathbf{H_2}$-), 1.12-1.36 (m, (10H ; 3H), (-(C$\mathbf{H_2}$)$_5$- ; R-C-O_2-CH_2-C$\mathbf{H_3}$)), 0.82-0.92 (m, 3H, -$(CH_2)_7$-C$\mathbf{H_3}$).

^{13}C NMR of copoly(dialkyl muconate)s:

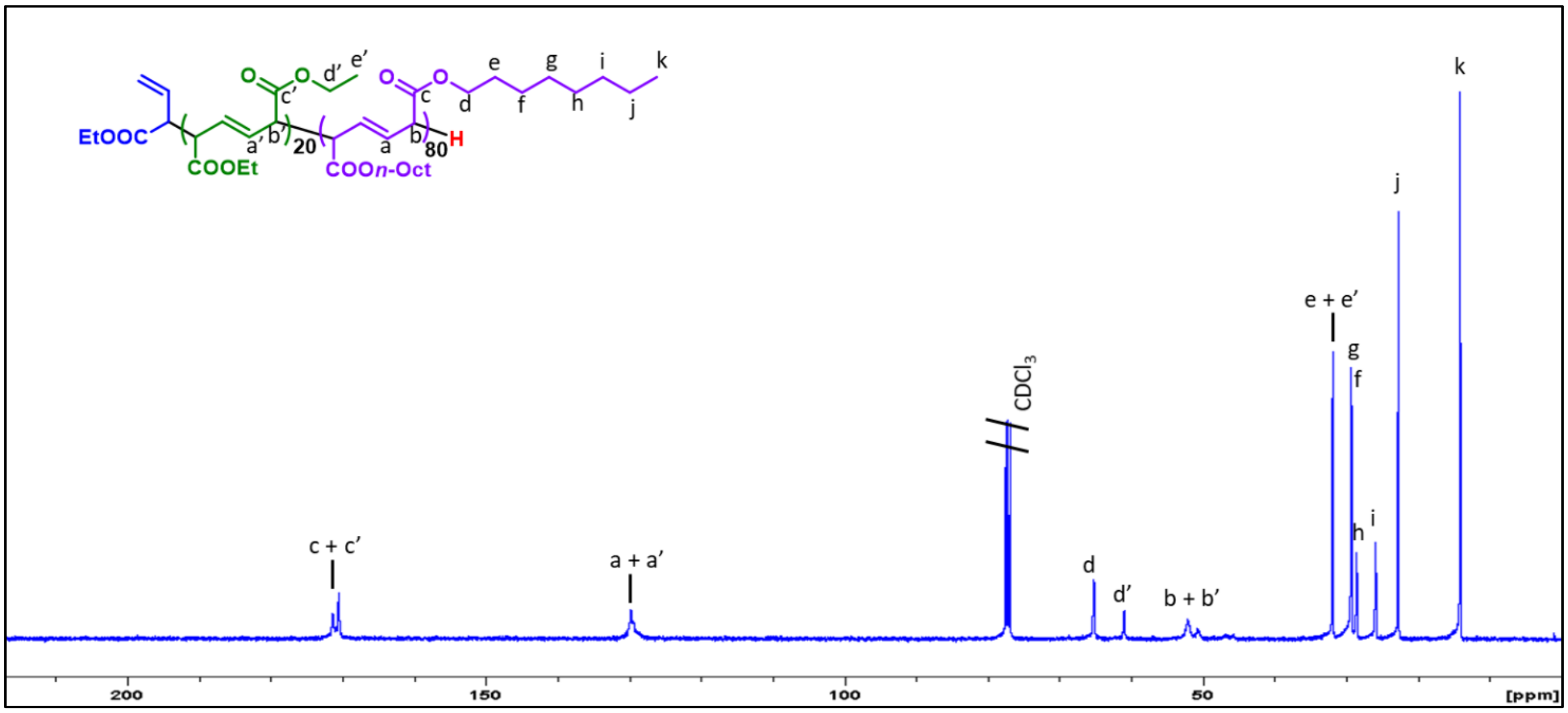

Figure S53. ^{13}C NMR of PDEM$_{20}$-*co*-PDOM$_{80}$ ($CDCl_3$, 400 MHz): PDEM: δ 170.20-171.90 (R-O_2-**C**-R), 127.81-130.45 (R-O_2-C-CH=**C**H), 60.62-61.32 (-**C**H_2-CH_3), 49.80-52.55 (R-O_2-C-**C**H=CH, trans), 45.00-47.60 (RO_2-C-**C**H=CH, cis), 14.04 (-CH_2-**C**H_3). PDOM: 128.00-131.60 (R-O_2-C-**C**H=CH), 65.23 (R-CO_2-**C**H_2-$(CH_2)_6$-CH_3), 49.99-53.15 (R-O_2-C-**C**H=CH, trans), 45.01-49.05 (R-O_2-C-**C**H=CH, cis), 31.86 (R-C-O_2-CH_2-**C**H_2-$(CH_2)_5$-CH_3), 29.30 (R-C-O_2-$(CH_2)_2$-**C**H_2-$(CH_2)_4$-CH_3), 29.26 (R-C-O_2- $(CH_2)_3$-**C**H_2-$(CH_2)_3$-CH_3), 28.52 (R-C-O_2-$(CH2)_4$-CH_2-$(CH_2)_2$-CH_3), 25.93 (R-C-O_2-$(CH_2)_5$-**C**H_2- CH_2-CH_3), 25.25 (R-C-O_2-$(CH_2)_6$-**C**H_2-CH_3), 22.66 (R-C-O_2-$(CH_2)_7$-**C**H_3).

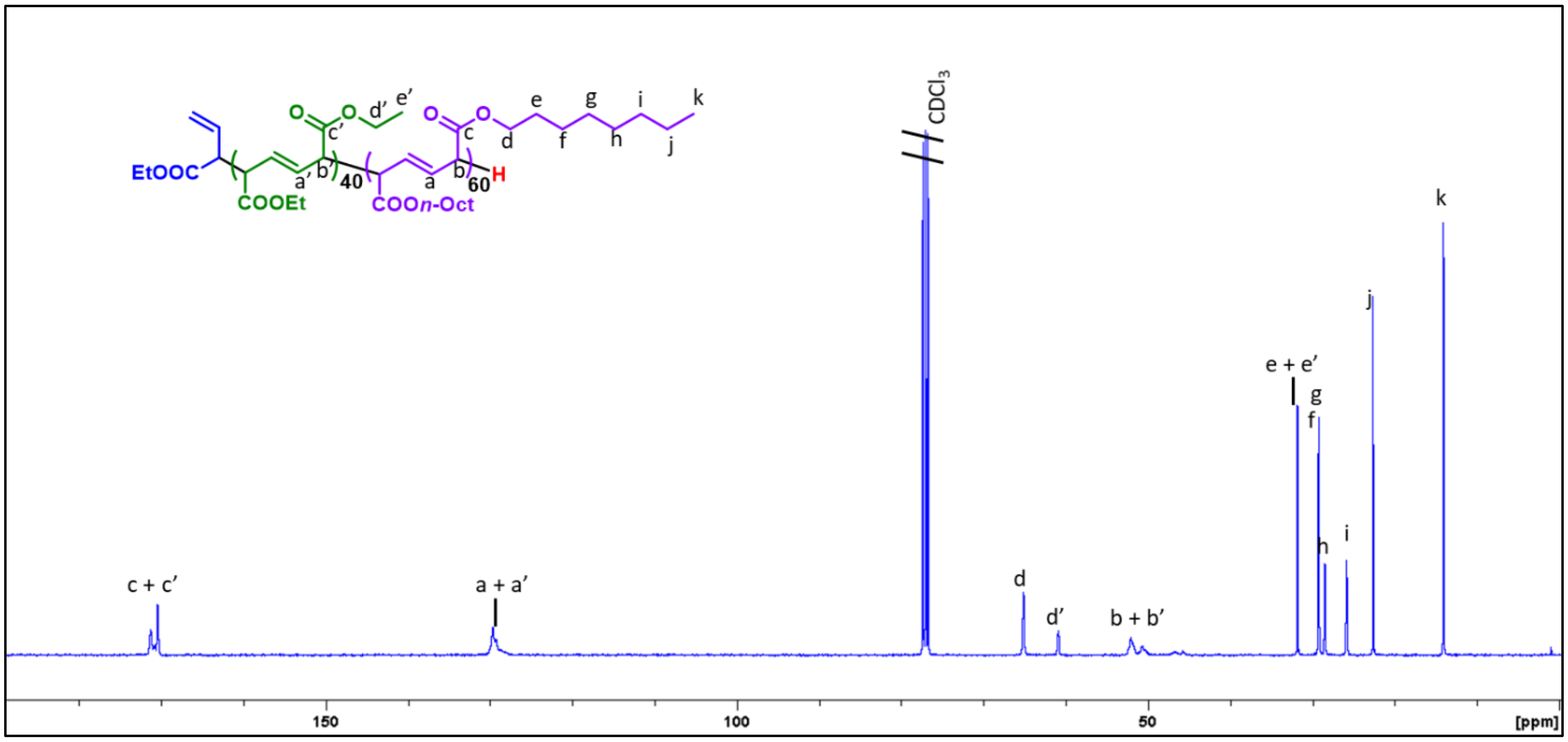

Figure S54. ^{13}C NMR of $PDEM_{40}$-*co*-$PDOM_{60}$ ($CDCl_3$, 400 MHz): PDEM: PDEM: δ 170.20-171.90 (R-O_2-C-R), 127.81-130.45 (R-O_2-C-CH=CH), 60.62-61.32 (-CH_2-CH_3), 49.80-52.55 (R-O_2-C-CH=CH, trans), 45.00-47.60 (RO_2-C-CH=CH, cis), 14.04 (-CH_2-CH_3). PDOM: 128.00-131.60 (R-O_2-C-CH=CH), 65.23 (R-CO_2-CH_2-$(CH_2)_6$-CH_3), 49.99-53.15 (R-O_2-C-CH=CH, trans), 45.01-49.05 (R-O_2-C-CH=CH, cis), 31.86 (R-C-O_2-CH_2-CH_2-$(CH_2)_5$-CH_3), 29.30 (R-C-O_2-$(CH_2)_2$-CH_2-$(CH_2)_4$-CH_3), 29.26 (R-C-O_2- $(CH_2)_3$-CH_2-$(CH_2)_3$-CH_3), 28.52 (R-C-O_2-(CH2)$_4$-CH_2-$(CH_2)_2$-CH_3), 25.93 (R-C-O_2-$(CH_2)_5$-CH_2- CH_2-CH_3), 25.25 (R-C-O_2-$(CH_2)_6$-CH_2-CH_3), 22.66 (R-C-O_2-$(CH_2)_7$-CH_3).

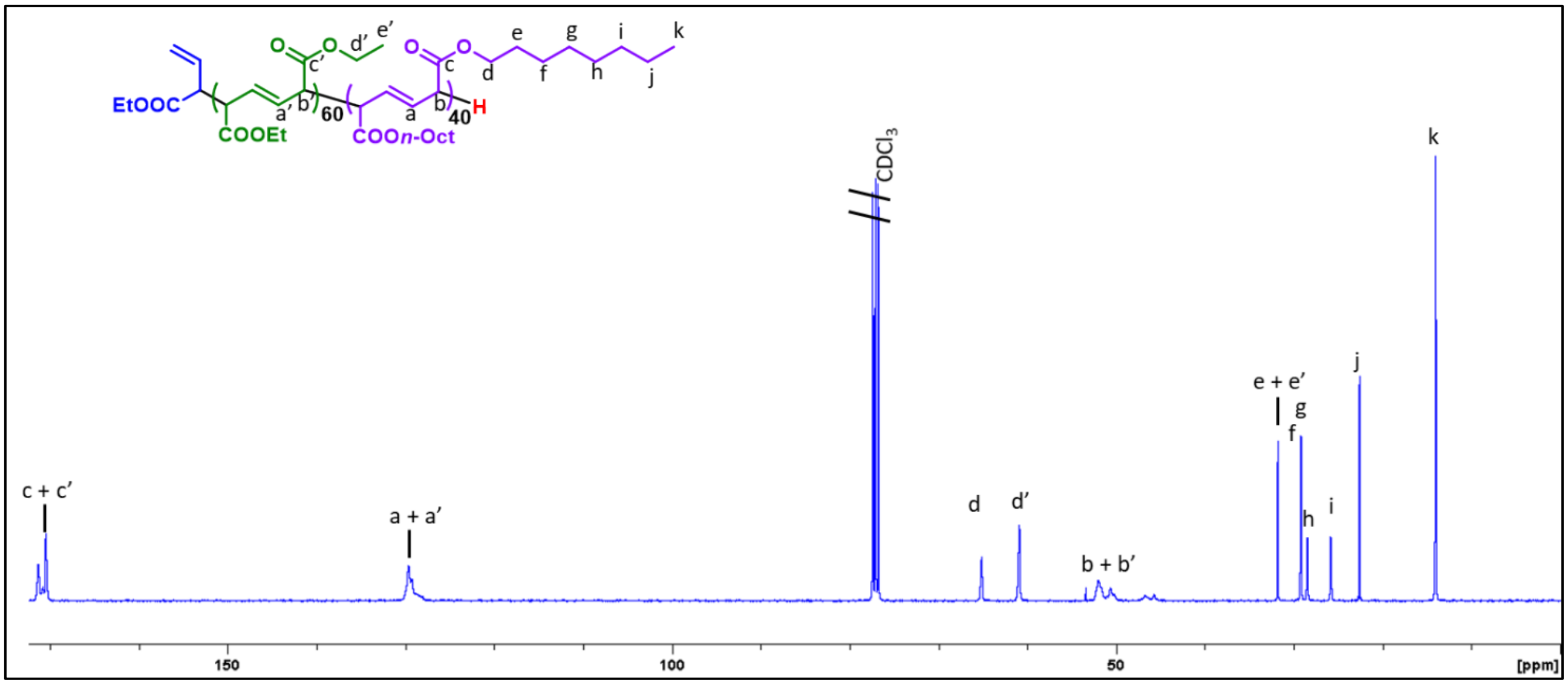

Figure S55. ^{13}C NMR of PDEM$_{60}$-*co*-PDOM$_{40}$ ($CDCl_3$, 400 MHz): PDEM: δ 170.20-171.90 (R-O_2-**C**-R), 127.81-130.45 (R-O_2-C-CH=**C**H), 60.62-61.32 (-**C**H_2-CH_3), 49.80-52.55 (R-O_2-C-**C**H=CH, trans), 45.00-47.60 (RO_2-C-**C**H=CH, cis), 14.04 (-CH_2-**C**H_3). PDOM: 128.00-131.60 (R-O_2-C-**C**H=CH), 65.23 (R-CO_2-**C**H_2-$(CH_2)_6$-CH_3), 49.99-53.15 (R-O_2-C-**C**H=CH, trans), 45.01-49.05 (R-O_2-C-**C**H=CH, cis), 31.86 (R-C-O_2-CH_2-**C**H_2-$(CH_2)_5$-CH_3), 29.30 (R-C-O_2-$(CH_2)_2$-**C**H_2-$(CH_2)_4$-CH_3), 29.26 (R-C-O_2- $(CH_2)_3$-**C**H_2-$(CH_2)_3$-CH_3), 28.52 (R-C-O_2-$(CH2)_4$-CH_2-$(CH_2)_2$-CH_3), 25.93 (R-C-O_2-$(CH_2)_5$-**C**H_2- CH_2-CH_3), 25.25 (R-C-O_2-$(CH_2)_6$-**C**H_2-CH_3), 22.66 (R-C-O_2-$(CH_2)_7$-**C**H_3).

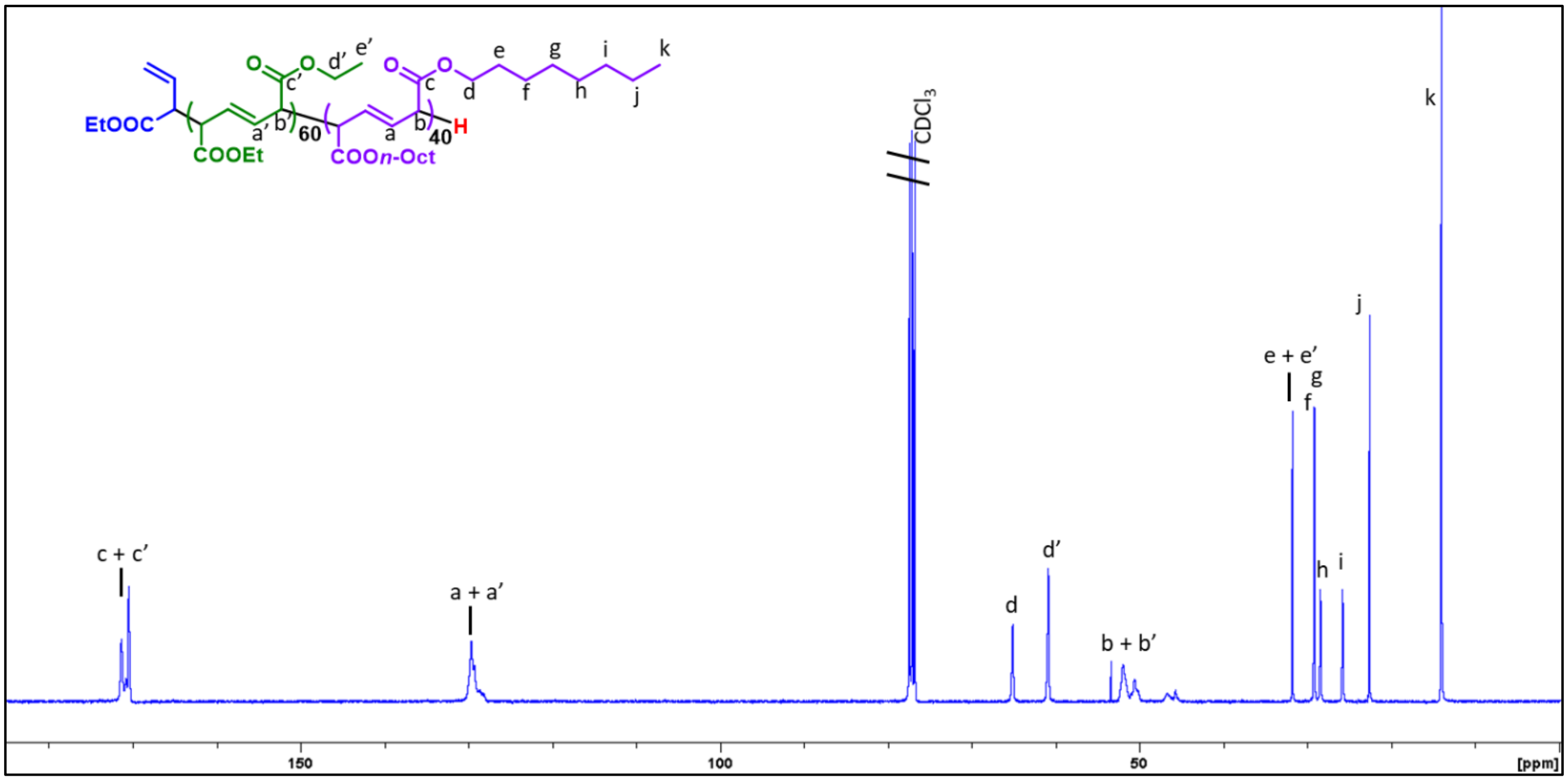

Figure S56. ^{13}C NMR of PDEM$_{80}$-*co*-PDOM$_{20}$ ($CDCl_3$, 400 MHz): PDEM: δ 170.20-171.90 (R-O_2-**C**-R), 127.81-130.45 (R-O_2-C-CH=**C**H), 60.62-61.32 (-**C**H_2-CH_3), 49.80-52.55 (R-O_2-C-**C**H=CH, trans), 45.00-47.60 (RO_2-C-**C**H=CH, cis), 14.04 (-CH_2-**C**H_3). PDOM: 128.00-131.60 (R-O_2-C-**C**H=CH), 65.23 (R-CO_2-**C**H_2-$(CH_2)_6$-CH_3), 49.99-53.15 (R-O_2-C-**C**H=CH, trans), 45.01-49.05 (R-O_2-C-**C**H=CH, cis), 31.86 (R-C-O_2-CH_2-**C**H_2-$(CH_2)_5$-CH_3), 29.30 (R-C-O_2-$(CH_2)_2$-**C**H_2-$(CH_2)_4$-CH_3), 29.26 (R-C-O_2- $(CH_2)_3$-**C**H_2-$(CH_2)_3$-CH_3), 28.52 (R-C-O_2-(CH2)$_4$-CH_2-$(CH_2)_2$-CH_3), 25.93 (R-C-O_2-$(CH_2)_5$-**C**H_2- CH_2-CH_3), 25.25 (R-C-O_2-$(CH_2)_6$-**C**H_2-CH_3), 22.66 (R-C-O_2-$(CH_2)_7$-**C**H_3).

References:

1. T. Dardé, É. Diomar, X. Schultze and D. Taton, *Angew Chem Int Ed*, 2024, **63**, e202411249.

2 . T. Kelen and F. Tüdos, *Journal of Macromolecular Science, Part A: Chemistry*, 1975, **9**, 1–27.

3. V. E. Meyer and G. G. Lowry, *Journal of Polymer Science*, 1965, **3**, 2843–2851.

4. T. G. Fox, *Bulletin of the American Physical Society*, 1956, **1**, 123.

5. *M. Van Den Brink, A. M. Van Herk, A.L. German, J. Polym. Sci. Part Polym. Chem.* 1999, **37** (20), 3793–3803.
